# Discovery and prioritization of variants and genes for kidney function in >1.2 million individuals

**DOI:** 10.1038/s41467-021-24491-0

**Published:** 2021-07-16

**Authors:** Kira J. Stanzick, Yong Li, Pascal Schlosser, Mathias Gorski, Matthias Wuttke, Laurent F. Thomas, Humaira Rasheed, Bryce X. Rowan, Sarah E. Graham, Brett R. Vanderweff, Snehal B. Patil, VA Million Veteran Program, Cassiane Robinson-Cohen, John M. Gaziano, Christopher J. O’Donnell, Cristen J. Willer, Stein Hallan, Bjørn Olav Åsvold, Andre Gessner, Adriana M. Hung, Cristian Pattaro, Anna Köttgen, Klaus J. Stark, Iris M. Heid, Thomas W. Winkler

**Affiliations:** 1 Department of Genetic Epidemiology, University of Regensburg, Regensburg, Germany; 2 Institute of Genetic Epidemiology, Department of Biometry, Epidemiology and Medical Bioinformatics, Faculty of Medicine and Medical Center–University of Freiburg, Freiburg, Germany; 3 K. G. Jebsen Center for Genetic Epidemiology, Department of Public Health and Nursing, Faculty of Medicine and Health, NTNU, Norwegian University of Science and Technology, Trondheim, Norway; 4 Department of Clinical and Molecular Medicine, NTNU, Norwegian University of Science and Technology, Trondheim, Norway; 5 BioCore - Bioinformatics Core Facility, Norwegian University of Science and Technology, Trondheim, Norway; 6 MRC Integrative Epidemiology Unit, Population Health Sciences, Bristol Medical School, University of Bristol, Bristol, UK; 7 Department of Biostatistics, Vanderbilt University Medical Center, Nashville, TN USA; 8 Department of Veteran’s Affairs, Tennessee Valley Healthcare System (626)/Vanderbilt University, Nashville, TN USA; 9 Department of Internal Medicine, Division of Cardiology, University of Michigan, Ann Arbor, MI USA; 10 Department of Biostatistics, University of Michigan School of Public Health, Ann Arbor, MI USA; 11 Center for Statistical Genetics, University of Michigan School of Public Health, Ann Arbor, MI USA; 12 Department of Computational Medicine and Bioinformatics, University of Michigan, Ann Arbor, MI USA; 13 Vanderbilt University Medical Center, Division of Nephrology and Hypertension, Vanderbilt Center for Kidney Disease and Integrated Program for Acute Kidney Injury Research, and Vanderbilt Precision Nephrology Program Nashville, Nashville, TN USA; 14 Massachusetts Area Veterans Epidemiology Research and Information Center (MAVERIC), VA Cooperative Studies Program, VA Boston Healthcare System, Boston, MA USA; 15 Department of Internal Medicine, Harvard Medical School, Boston, MA USA; 16 VA Cooperative Studies Program, VA Boston Healthcare System, Boston, MA USA; 17 Department of Human Genetics, University of Michigan, Ann Arbor, MI USA; 18 Department of Nephrology, St. Olavs Hospital, Trondheim University Hospital, Trondheim, Norway; 19 Department of Endocrinology, Clinic of Medicine, St. Olavs Hospital, Trondheim University Hospital, Trondheim, Norway; 20 Institute of Clinical Microbiology and Hygiene, University Hospital Regensburg, Regensburg, Germany; 21 Eurac Research, Institute for Biomedicine (affiliated with the University of Lübeck), Bolzano, Italy; 22 Department of Epidemiology, Johns Hopkins Bloomberg School of Public Health, Baltimore, MD USA

**Keywords:** Genome-wide association studies, Chronic kidney disease, Genetics research

## Abstract

Genes underneath signals from genome-wide association studies (GWAS) for kidney function are promising targets for functional studies, but prioritizing variants and genes is challenging. By GWAS meta-analysis for creatinine-based estimated glomerular filtration rate (eGFR) from the Chronic Kidney Disease Genetics Consortium and UK Biobank (n = 1,201,909), we expand the number of eGFRcrea loci (424 loci, 201 novel; 9.8% eGFRcrea variance explained by 634 independent signal variants). Our increased sample size in fine-mapping (n = 1,004,040, European) more than doubles the number of signals with resolved fine-mapping (99% credible sets down to 1 variant for 44 signals, ≤5 variants for 138 signals). Cystatin-based eGFR and/or blood urea nitrogen association support 348 loci (n = 460,826 and 852,678, respectively). Our customizable tool for Gene PrioritiSation reveals 23 compelling genes including mechanistic insights and enables navigation through genes and variants likely relevant for kidney function in human to help select targets for experimental follow-up.

## Abstract (web summary)

Identifying causal variants and genes in genome-wide association studies remains a challenge, an issue that is ameliorated with larger sample sizes. Here the authors meta-analyze kidney function genome-wide association studies to identify new loci and fine-map loci to home in on variants and genes involved in kidney function.

## Introduction

Chronic kidney disease (CKD) is a leading cause of morbidity and mortality worldwide, and a major public health problem with the prevalence of >10% in the adult population in developed countries^1,2^. Although many underlying causes of CKD such as diabetes, hypertension, vascular disease or glomerulonephritis are known, CKD aetiology remains in most cases unclear. Moreover, knowledge about the underlying molecular mechanisms causing progressive loss of renal function is so far insufficient, resulting in a lack of therapeutic targets for drug development^3^.

A defining parameter of CKD is decreased glomerular filtration rate, which can be estimated from the serum creatinine level^4^. Estimated creatinine-based GFR (eGFRcrea) has a strong heritable component^5^. Twin studies estimated a broad-sense heritability for eGFRcrea of 54%^5^. Recently, a GWAS meta-analysis of eGFRcrea conducted by the CKD Genetics (CKDGen) Consortium identified 264 associated genetic loci^6,7^. The lead variants at identified loci explained nearly 20% of eGFRcrea’s genetic heritability^7^. A substantial fraction of the missing heritability is expected to be attributed to low-frequency and rare variants^8,9^, which require even larger GWAS sample sizes to be identified. While eGFRcrea is a useful marker of kidney function in clinical practice, the underlying serum creatinine is a metabolite from muscle metabolism^10,11^ and thus may not only reflect kidney function. It is a major challenge in eGFRcrea GWAS to dissect mechanisms of biomarker metabolism from modulators of kidney function. Alternative kidney function biomarkers include blood urea nitrogen (BUN), which had supported 147 of the 264 eGFRcrea GWAS associations previously^7^. GFR estimated by serum cystatin C (eGFRcys) may be a better marker of GFR, but can also be affected by factors other than GFR (e.g., inflammation, obesity, diabetes^12^) and had a limited role in kidney function GWAS^13^ due to high costs and small data, so far.

Another challenge of GWAS is the large number of genes and variants underneath association signals. Numerous approaches for bioinformatic characterisation of identified loci yield an abundance of potentially relevant information^14–16^. Experimental follow-up is pivotal to generate mechanistic insights as a stepping stone to clinical applications, but these experiments can usually only be performed for a limited number of variants and genes. Fine-mapping of GWAS association signals aims at narrowing down to the few variants driving signals and fine-mapping resolution has been shown to benefit most from increased GWAS sample size^17^. Focusing the bioinformatic characterisation on refined association signals can help prioritise genes and variants for experimental follow-up.

We here improve the interpretability of associated eGFRcrea loci by high-resolution fine-mapping of eGFRcrea loci via doubling the sample size for fine-mapping compared to the previous work^7^ and by introducing genetic eGFRcys data in a large sample size. For this, we integrate GWAS data from the CKDGen Consortium^7^ and UK Biobank (UKB)^18^ on eGFRcrea in >1.2 million individuals of predominantly European ancestry, on eGFRcys in >400,000, on BUN in >800,000 individuals, and fine-mapping in >1,000,000 individuals. We construct a tool for Gene PriortiSation (GPS) summarising results from systematic bioinformatic follow-up, in order to guide the selection of relevant targets for experimental follow-up (Supplementary Fig. 1).

## Results

### GWAS meta-analysis identified 201 novel non-overlapping loci for eGFRcrea

To identify genetic variants associated with eGFRcrea, we conducted a linear mixed model-based GWAS^19^ of eGFRcrea in UKB (European ancestry, *n* = 436,581, Supplementary Data 1, imputed to Haplotype Reference Consortium^20^ and UK10K panels^21^) and meta-analysed results with the CKDGen Consortium data (mostly European ancestry, *n* = 765,348, imputed to Haplotype Reference Consortium^20^ or 1000 Genomes^22^)^7^, for a total sample size of 1,201,909 individuals (Supplementary Fig. 1, “Methods”). From the 13,633,840 analysable variants with a minor allele frequency (MAF) of >=0.1%, we selected genome-wide significant (GWS, *P* < 5 × 10^−8^) variants and derived non-overlapping loci using a stepwise approach (locus region defined by the first and last genome-wide significant variant +/−250 kb, “Methods”).

**Fig. 1:**
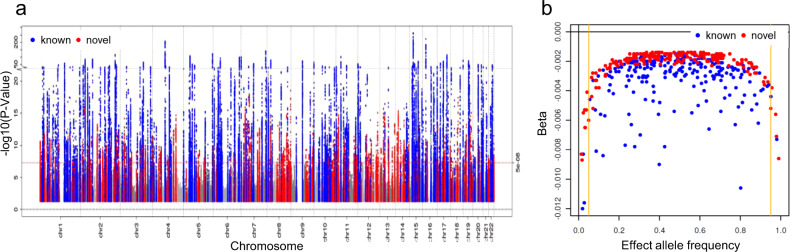
Primary meta-analysis for eGFRcrea identified 424 loci, including 201 novel loci. Shown are results from our primary meta-analysis for eGFRcrea (*n* = 1,201,929). We identified 424 loci with genome-wide significance (*P* < 5 × 10^−8^), including 223 known (previous GWAS^7^) and 201 novel (marked in blue and red, respectively). **a** Manhattan plot shows –log_10_ association *P* value for the genetic effect on eGFRcrea by chromosomal base position (GRCh37). The red dashed line marks genome-wide significance (5 × 10^−8^). *P* values are two-sided and were derived using a Wald test. **b** Scatterplot comparing eGFRcrea effect sizes versus allele frequencies for the 424 identified locus lead variants (orange lines at 5% and 95% allele frequency). Effect sizes and allele frequencies were aligned to the eGFRcrea-decreasing alleles.

We identified 424 non-overlapping loci: 201 were novel and 223 were known (Fig. 1a, Supplementary Data 2 and Supplementary Fig. 2; all well-imputed in UKB, info >0.9). We considered a locus as known if at least one GWS variant resided within one of the 264 loci previously identified^7^ (“Methods”). Only three of the 264 loci from Wuttke et al.^7^ barely missed genome-wide significance in our meta-analysis, which can be attributed to chance (*P* < 7.5 × 10^−7^, Supplementary Data 3). We observed 19 loci led by low-frequency variants (MAF < 5%) compared to seven such loci in the previous GWAS^7^ (Fig. 1b). Sensitivity meta-analyses restricting to individuals of European ancestry (*n* = 1,004,040) demonstrated similar association results for the 424 identified lead variants in European ancestry alone compared to the primary meta-analysis (Supplementary Data 2 and Supplementary Fig. 3).

All 424 lead variants showed directionally consistent nominal significance (*P* < 0.05, same effect direction) in UK Band in CKDGen, when evaluated separately (Supplementary Data 2). We were also interested in independent evidence for the association of the 424 lead variants with eGFRcrea. We gathered independent data from three studies for the second meta-analysis in 417,288 individuals (Million Veterans Program, MVP, *n* = 300,680; Michigan Genomics Initiative, MGI, *n* = 47,219; HUNT, *n* = 69,389; “Methods”, Supplementary Data 1). Power calculation showed that, despite the large sample size, power was not sufficient for a formal replication of novel loci at a Bonferroni-corrected significance level of *α* = 0.05/424 (Supplementary Note 1 and Supplementary Fig. 4). This was due to the larger phenotypic variance in two of these studies that were hospital-based as compared to population-based studies. When judged at one-sided*P* < 0.05, we found 361 of the 424 identified lead variants supported in the second meta-analysis (145/201 novel, 216/233 known) and 377 with *P* < 5 × 10^−8^ in the combined primary plus second meta-analysis (*n* = 1,619,217; Supplementary Data 4).

Taken together, two meta-analyses were undertaken for eGFRcrea; the primary meta-analysis (*n* = 1,201,930) showed 424 non-overlapping loci (201 novel and 223 known) at the significance level of *P* < 5 × 10^−8^; the second meta-analysis (*n* = 417,288) independently supported eGFRcrea association of 361 (of 424) lead variants (145/201 novel and 216/223 known) at one-sided*P* < 0.05. Given that there is a risk for excessive exclusion of false negatives, when the primary meta-analysis is very large (>1 M) and data for formal replication limited^23^, all the list of loci identified by the primary meta-analysis were subjected to downstream analyses for the purpose of prioritising candidate genes as comprehensive as possible.

### Association of identified variants with alternative kidney function biomarkers

**Fig. 2:**
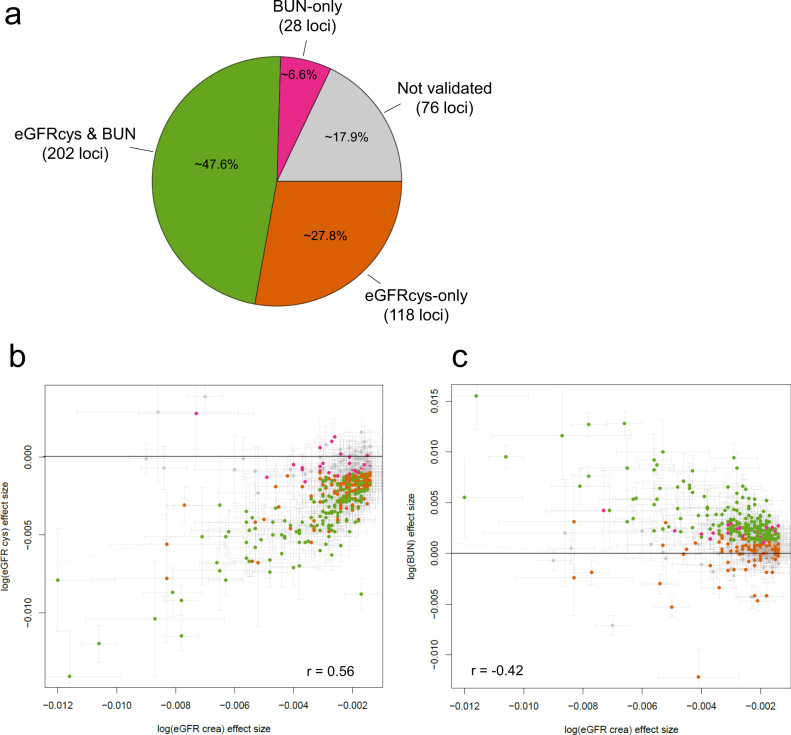
Supporting alternative biomarker association for 348 loci. Shown are results from our evaluation of alternative kidney function biomarker association for the 424 locus lead variants to establish loci with likely kidney function relevance. We classified each of the 424 variants as “validated” by BUN and/or eGFRcys based on a nominal significant association (*P* < 0.05) with consistent effect direction for BUN (*n* = 852,678, i.e. opposite effect to eGFRcrea) and/or eGFRcys (*n* = 460,826, i.e. same effect direction as eGFRcrea). We validated 348 of the 424 loci and thus more than doubled the number of loci with additional biomarker evidence compared to previous work (147 loci previously based on BUN-only^7^). **a** Pie chart showing the classification of the 424 lead variants as “validated” by eGFRcys and/or BUN effects. **b** Scatterplot comparing effect sizes for eGFRcrea and eGFRcys with 95% confidence intervals (green: eGFRcys and BUN validated, brown: only eGFRcys-validated, magenta: only BUN validated, grey: not validated). **c** Scatterplot comparing effect sizes for eGFRcrea and BUN (colouring analogous to **b**). The correlation coefficients between effect sizes shown are Spearman correlation coefficients and were based on the 348 validated loci lead variants. Genetic effect sizes are presented with error bars +/− 1.96* standard error of the genetic effect size estimate.

A genetic association with eGFRcrea can be related to kidney function or to creatinine metabolism. We thus sought the support of the 424 lead variants’ association with eGFRcrea by association with alternative biomarkers to substantiate the detected locus as likely related to kidney function. We analysed the 424 variants for association with eGFRcys and BUN in UKB (*n* = 436,765 and 436,500, respectively) and meta-analysed results with existing CKDGen summary statistics for these biomarkers^7,13^ (*n* = 24,061 and 416,178, respectively; combined *n* = 460,826 and 852,678, respectively; “Methods”). We defined a variant’s eGFRcrea association as validated by eGFRcys/BUN when we observed a directionally consistent, nominally significant association with eGFRcys and/or BUN (*P* < 0.05, same effect direction for eGFRcys and/or opposite effect direction for BUN). Of the 424 lead variants, 348 were eGFRcys/BUN-validated (118 only by eGFRcys, 28 only by BUN, 202 by both, Fig. 2a, Supplementary Data 5). When compared to previous work^7^ having validated 147 loci with BUN association in 416,178 individuals, we more than doubled the number of eGFRcrea loci supported by as likely kidney function related. While the proportion of BUN-validated loci among the 424 was 54%, similar to Wuttke et al.^7^, we found 75% as eGFRcys-validated with a much lower sample size for eGFRcys compared to BUN. Effect sizes of eGFRcrea showed higher correlation with eGFRcys than BUN (*r* = 0.56 and −0.42, respectively, Fig. 2b, c).

In summary, ~82% (348 loci) of the 424 identified eGFRcrea loci were validated by association with at least one alternative biomarker and thus classified as likely relevant for kidney function. Our results underscore the value of eGFRcys to kidney function GWAS, the integration of which at this scale of sample size was done here for the first time—to our knowledge.

### Secondary signals and fine-mapping in European ancestry

We were interested in narrowing down the association signals across the 424 identified loci and thus evaluated each locus for multiple independent signals followed by determining the variants in each signal that were most likely driving the respective association. Our GWAS included individuals predominantly from European ancestry (~84%) and an appropriate trans-ethnic linkage disequilibrium (LD) reference panel was lacking. Our sensitivity analyses had shown that the identified locus associations were not driven by these other ancestries and associations were rather stable when restricting to European ancestry individuals (see above). For these reasons, we restricted the following fine-mapping analyses to individuals of European ancestry (*n* = 1,004,040) and used a random subset of 20,000 unrelated individuals of European ancestry from UKB as LD reference panel (“Methods”).

**Fig. 3:**
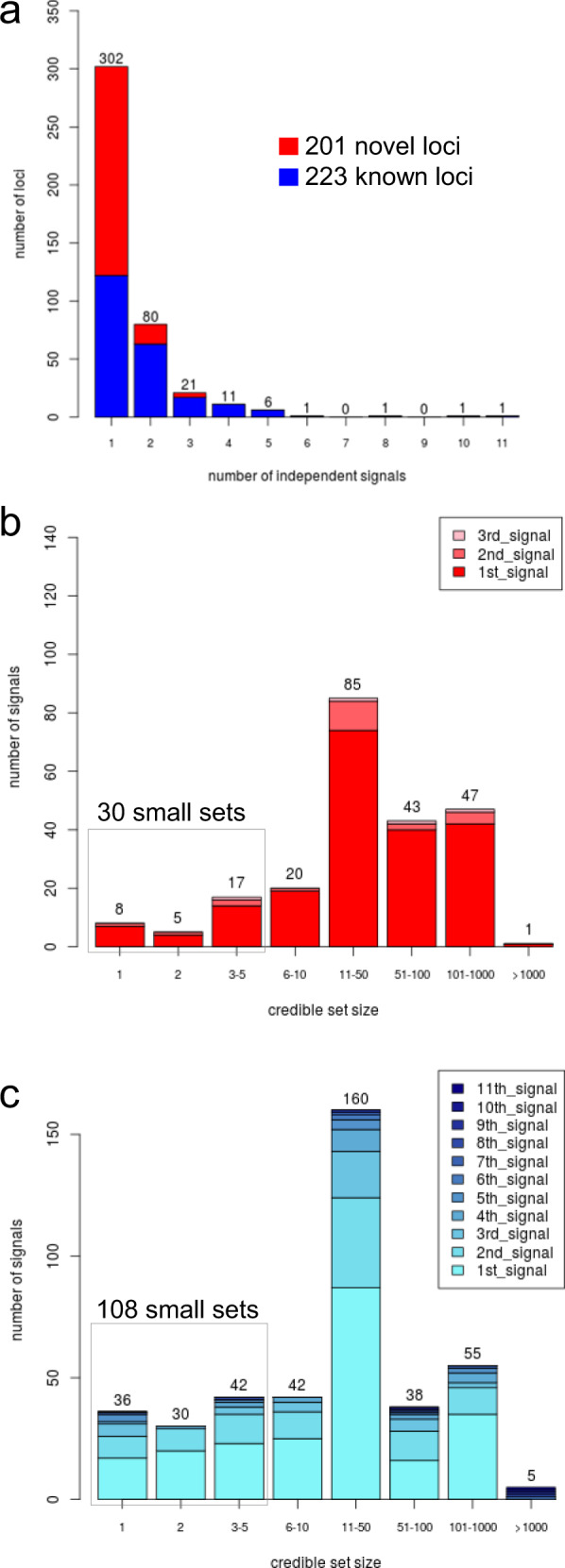
Fine-mapping of 634 independent signals by credible set variants including 138 with small credible set size. For the 424 identified eGFRcrea loci, we derived 634 independent signals by approximate conditional analyses with GCTA^24^ and, for each signal, 99% credible sets of variants using the method by Wakefield^25^ based on the European-only meta-analysis results (*n* = 1,004,040). **a** Distribution of the number of signals per 424 loci. **b** Distribution of credible set sizes for the 226 signals at novel loci. **c** Distribution of credible set sizes for the 408 signals at known loci. Colour in panels **b** and **c** denotes the order in which the signal appeared in the stepwise conditional analysis. Of the 634 signals, 138 were successfully fine-mapped down to a small credible set (i.e. <=5 variants) including 44 that contained exactly one variant.

To identify distinct association signals arising from multiple causal variants in the same locus, we conducted conditional analyses using GCTA^24^ at each of the 424 non-overlapping loci (“Methods”). We identified 634 independent signals across the 424 loci (*P* value conditioned on other signal-index variants <5 × 10^−8^, Fig. 3a, Supplementary Data 6). These included three rare variants (MAF <1%), all of which were well-imputed in UKB (info >0.9). At least two independent signals were observed at 21 novel (Supplementary Fig. 5) and at 101 known loci. When more signals are identified for a known locus than observed previously, this provides new insights on additional causal variants for known loci. For example in the known *UMOD/PDILT* locus, we observed four independent signals, two novel and two previously described^7^ (Supplementary Fig. 6). This suggests two new causal variants in this locus well-known for kidney function. Also, the locus near *PKHD1* showed four independent signals compared to one signal previously suggesting three further causal entities.

To narrow down association signals, we calculated the posterior probability of association (PPA)^25^ for each variant and constructed 99% credible sets of variants at each of the 634 signals (Supplementary Data 7, “Methods”). Among the 424 primary lead variants, 373 were precisely the variant with the highest PPA or were contained in the credible set (215 or 158 variants, respectively; Supplementary Fig. 7). The median size of credible sets was 23 (total of 38,306 credible variants at the 634 signals). Credible sets for known loci were on average smaller compared to the previous GWAS^7^ (median of 17 compared to 26 variants previously, Supplementary Data 8).

A hallmark of effective fine-mapping of association signals are small 99% credible sets (i.e. ≤5 variants) as these enable the focus on a limited number of variants containing the causal variant with 99% probability (given there is one causal variant and that this variant is among those analysed). We observed 138 signals with small 99% credible sets, of which 30 mapped to a novel locus, 88 mapped to a novel signal or a previously larger set in a known locus (i.e. “newly small”), and 20 mapped to a previously reported small set^7^ (i.e. “known-small”, Table 1, Fig. 3b, Supplementary Data 8). The 138 include 44 single-variant sets, which are particularly interesting because these variants have more than 99% probability of being the causal variant given the association data by definition. Among the 44 single sets, 8 mapped to novel loci, 22 were “newly single” (at known loci) and 14 were “known-single”^7^.

**Table 1:** Summary of annotation of the 138 single or small 99% credible variant sets.

	44 (37) single sets (1 variant)			94 (83) sets with 2–5 variants		
	8 (8) at novel loci	36 (29) at known loci		22 (19) at novel loci	72 (64) at known loci	
Among 99% credible set variants		22 (17) newly single	14 (12) known-single		47 (43) newly small	25 (21) known-small
Any protein-relevant variant	3 (3)	5 (3)	6 (5)	7 (5)	6 (6)	3 (1)
• Stop-gained/ stop-lost/non-synonymous	2 (2)	2 (2)	5 (4)	4 (4)	3 (3)	2 (1)
• Canonical-splice/noncoding-change/synonymous/splice-site	0	0	0	0	0	0
• Other consequence	1 (1)	3 (1)	1 (1)	3 (1)	3 (3)	2 (0)
Any kidney-tissue regulatory variant	0	0	0	2 (1)	7 (7)	0
• eQTL in glomerulus (NEPTUNE)	0	0	0	1 (1)	1 (1)	0
• eQTL in tubulo-interstitium (NEPTUNE)	0	0	0	1 (0)	6 (6)	0
• eQTL in kidney tissue (GTEx)	0	0	0	0	1 (1)	0
• sQTL in kidney tissue (GTEx)	0	0	0	0	1 (1)	0
Any protein-relevant or kidney-tissue regulatory variant	3 (3)	5 (3)	6 (5)	8 (6)	12 (12)	3 (1)
Any other tissue regulatory variant	5 (5)	13 (11)	11 (10)	15 (14)	34 (31)	18 (14)
• eQTL in other tissue (GTEx)	5 (5)	12 (10)	11 (10)	15 (14)	34 (31)	18 (14)
• sQTL in other tissue (GTEx)	1 (1)	10 (8)	7 (6)	11 (10)	20 (17)	7 (5)

For the 138 identified eGFRcrea signals mapping to single or small (2–5 variants) 99% credible variant sets (fine-mapping in *n* = 1,004,040 individuals), we applied bioinformatic follow-up to the credible variants. Shown are the number of signals containing a credible variant targeting a gene in the locus by being (i) relevant for the protein (i.e., CADD score ≥15, variant within gene, Supplementary Data 9), (ii) relevant for regulatory function in kidney tissue (i.e., eQTL in NEPTUNE glomerular or tubule-interstitial tissue, Supplementary Data 10; or eQTL/sQTL in GTEX kidney tissue, Supplementary Data 11), or (iii) relevant for regulatory function in other non-kidney tissue (i.e. eQTL/sQTL in GTEx non-kidney tissues, Supplementary Data 12). Shown in brackets is the number of signals mapping to eGFRcys/BUN-validated loci.

We annotated all 38,306 credible set variants for being relevant for (i) functional consequence on the protein for variants within the gene (CADD score^26^ >= 15 Supplementary Data 9, “Methods”) or (ii) regulatory function as expression or splicing on gene within the same locus, in kidney tissue or any non-kidney tissue (FDR < 5%; Supplementary Data 10–12, “Methods”). Among the 138 signals with small credible sets, 36 contained at least one protein-relevant or kidney-tissue-regulatory variant (Table 1, Supplementary Data 13). These included 27 signals with variants mapping to novel loci, newly single or newly small sets, which provide new ideas on causal variants or increased certainty in variant causality.

Overall, decreased median credible set size and a substantially larger number of small credible sets compared to previously (138 versus 58^7^) document the increased fine-mapping ability of the larger sample size (here *n* = 1,004,040 versus 567,460 previously).

### Gene PrioritiSation (GPS)

The credible set variants that are relevant to the protein or regulatory function suggest the respective mapping gene as causal: mapping via the protein-relevant variant in the gene or via the variant modulating gene expression or splicing as *cis*-eQLT/sQTL for genes within the same locus. We selected the genes overlapping the 424 identified locus regions (i.e., interval between first and last GWS variant of a locus +/−250 kb) yielding 5906 genes (average 8 genes per locus; Supplementary Data 8). For these genes, we generated a sortable and searchable table for Gene PrioritiSation (GPS, Supplementary Data 14), by indicating genes that (i) mapped to a relevant variant (defined above, Supplementary Data 9–12) and/or (ii) had a kidney-related phenotype in mice or human (Mouse Genome Informatics^27^, MGI, Online Mendelian Inheritance in Man^28^, OMIM®, Mendelian kidney disease^29^, Supplementary Data 15–16, “Methods”). Among the 5906 genes, we found 2777 with at least one GPS feature (Supplementary Data 14a). This illustrates limited dimension-reduction when considering genes with any relevant feature and the further need for prioritisation.

**Fig. 4:**
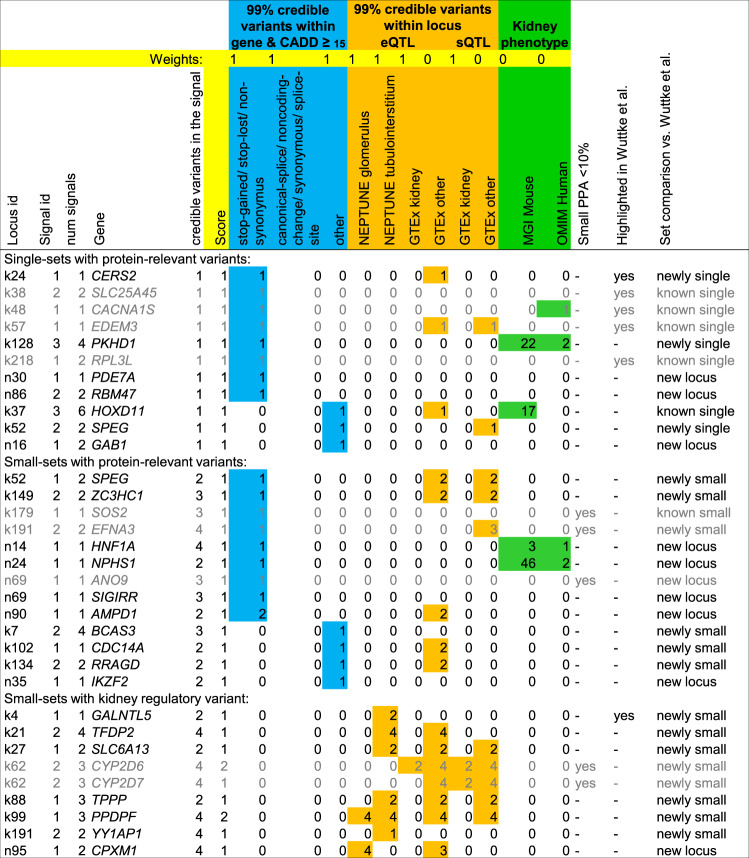
Results from Gene PrioritiSation (GPS) yields 32 genes. By querying our GPS (Supplementary Data 14), we identified 32 genes that are mapping to eGFRcys/BUN-validated loci and to a small credible set (≤5 variants) that contains a protein-relevant variant within the gene (CADD ≥15) or a kidney-tissue regulatory variant (eQTL in NEPTUNE glomerulus or tubule-interstitial tissue; eQTL or sQTL in GTEx kidney tissue). Shown is the locus information (locus id, signal id, number of signals in the locus and the number of credible variants in the signal), variant information for credible variants within the gene (functional annotation, blue), for regulatory credible variants (regulatory annotation, orange) and gene information for kidney-related phenotypes (in mouse or human, green). Genes are grey if the PPA of the relevant variant is <10% or if the gene was previously highlighted by Wuttke et al. without additional evidence^7^. An alternative result limited to variants that are available in the mostly European CKDGen consortium meta-analysis is shown in Supplementary Fig. 8.

To search for genes mapping to protein-relevant or kidney-tissue-regulatory variants from small credible sets in eGFRcys/BUN-validated loci, we utilised our GPS (“eGFRcys/BUN = yes”, “cred set size ≤5”, weights for protein-relevant variants or kidney-tissue regulatory variants, “score>=1”). We found 32 such genes, 11 genes mapping to a single-variant 99% credible set, 21 additional genes to a set of size 2–5 (Fig. 4 and Supplementary Fig. 8).

All 11 single-set genes mapped to protein-relevant variants (CADD≥15), none to a kidney-tissue regulatory variant. These 11 variants have each a 99% probability of being the causal variant by definition and thus provide immediate mechanistic insights and implicate the respective gene as likely causal: (i) 8 variants were protein-altering (known-single in *EDEM3, RPL3L, SLC25A45* and *CACNA1S*; newly single in *CERS2* and *PKHD1*; novel locus single in *PDE7A* and *RBM47*). While the *CERS2* variant rs267738 was implicated before in a previous credible set of size 5, our single-variant credible set zoomed onto precisely this protein-altering variant. This provides now substantial certainty into this variant being causal (now PPA = 99%, previous PPA = 46%). The locus near *PKHD1* showed four independent signals and was not fine-mapped previously, which fostered the identification of a new single-variant credible set pointing to the protein-altering variant rs76572975, *PKHD1* is known for Mendelian kidney disease^29^. While this missense variant is declared “benign” in ClinVar^30^ for monogenic kidney disease, its impact on kidney function in the general population is not yet explored. The protein-altering variants in *PDE7A* and *RBM47* implicate two genes that have not yet been reported for kidney function. (ii) 3 variants had “other” CADD≥15 consequences (known-single in *HOXD11*; newly single in *SPEG*; novel locus single in *GAB1*). *HOXD11* is a reported kidney-developmental gene in mice^31^, but without human evidence so far. The *SPEG* gene has two signals, one single-variant credible set pointing to an intronic variant (rs112068790) and the other credible set of size 2 containing a protein-altering variant (rs55760516). For *SPEG*, there is no knowledge about kidney involvement so far.

For the 21 additional genes mapping to credible sets of size 2–5, we had multiple variants with interesting predicted function (Fig. 4 and Supplementary Note 2). These include eight genes mapping to protein-altering variants (known-small: *SOS2*, newly small: *EFNA3* and *ZC3HC1*; novel locus: *AMPD1, ANO9, HNF1A, NPHS1* and *SIGIRR*), four genes mapping to “other” CADD≥15 variants (newly small: *BCAS3*, *CDC14A* and *RRAGD*; novel locus: *IKZF2*) and nine genes mapping to eQTL/sQTL variants in kidney tissue (newly small: *CYP2D6*, *CYP2D7, GALNTL5*, *PPDPF, SLC6A13*, *TFDP2*, *TPPP*, *YY1AP1*; novel locus: *CPXM1*). Particularly compelling is the novel locus with the highly likely causal protein-altering variant in *NPHS1* (rs3814995), a gene known for the rare Mendelian disorder Nephrotic syndrome type 1^32^. The protein-altering variant points to a common variant associated with a kidney phenotype in the general population similar to the *PKHD1* variant described above. Also very interesting is the *HNF1A*, which is known for mutations to cause diabetes MODY type 3^33^. The highlighted less-frequent protein-altering variant (rs1800574, MAF = 3.0%, PPA = 48%) is not directly connected to the rare Mendelian disease but associated with a higher risk for diabetes type 2^34^ and serum urate levels^35^. *HNF1A* knockout mice show kidney dysfunction^36^. Interestingly, two credible sets consisted of a pair of eQTL-variants in tubolo-interstitium with shared high PPA due to the high correlation (*GALNTL5*, 2 variants, PPA = 49.98% each, *r*² = 1.00; *SLC6A13*, 2 variants, PPA = 51.9% and 47.5%, *r*² = 0.99). These variants would have been missed when restricting to PPA≥80% or mostly missed with PPA ≥50%. The eQTL-variants for *GALNTL5* resided underneath the previously reported colocalization signal for the same tissue^7^, which effectively pinpoints a likely causal variant for this colocalization, and the eQTLs for *SLC6A13* were novel.

Overall, among the 32 highlighted genes, 23 genes showed novel evidence compared to previous work^7^ and adequate fine-mapping resolution (PPA ≥10%) for the protein-relevant or kidney-tissue regulatory variant (Table 2). These 23 genes implicate new evidence as human association validated targets or improved certainty, which provide now starting points for experimental studies.

**Table 2:** Highlighted genes with novel evidence for kidney function.

Gene	Variant (EAF, PPA), consequence (CADD PHRED)	Novelty
*Single sets*		
Known single		
* HOXD11*	rs863678 (0.64, 99.9%), 3’ UTR (18.4)	Not further described previously as “other CADD>=15” previously
Newly single		
* CERS2*	rs267738 (0.46, 99.1%), p.Glu115Ala (32.0)	Previous cred set size=5 (previous PPA = 46%)^7^; rs267738 reported in previous GWAS^7^ and for rate of albuminuria^67^
* PKHD1*	rs76572975 (0.024, 99.7%), p.Arg3842Leu (23.8)	Not fine-mapped previously^7^; rs76572975 as less-frequent variant in rare Mendelian disorder gene
* SPEG*	rs112068790 (0.97, 99.2%), intron (18.3); rs55760516 (0.67, 39.8%), p.Gly2790Arg (22.3)	1st signal newly single, 2nd signal newly small (cred set size = 2), previously one signal with cred set >5^7^; experimental link to kidney function unknown
Novel locus		
* GAB1*	rs139323761 (0.027, 99.9%), Intron (21.9)	Experimental link to kidney function unknown
* PDE7A*	rs11557049 (0.065, 99.9%), p.Gly76Glu (24.0)	Experimental link to kidney function unknown
* RBM47*	rs35529250 (0.006, 99.8%), p.Gly538Arg (28.5)	Experimental link to kidney function unknown
*Sets 2–5*		
Newly small		
* BCAS3*	rs9905761 (0.81*, 36.9%), Intron (15.2)	Experimental link to kidney function unknown
* CDC14A*	rs17420882 (0.72,93.2%), Intron (16.2)	Experimental link to kidney function unknown
* GALNTL5*	rs6464165 (0.71,49.9%), eQTL tubulo-interstitial; rs10224210 (0.71, 49.9%), eQTL tubulo-interstitial	Previous coloc tubulo-interstitial (PPH4 = 98%)^7^, coloc confirmed (PPH4 = 98.7%), experimental link to kidney function unknown
* PPDPF*	rs72629024 (0.85, 85.1%), eQTL tubulo-interstitial/glomerular	New coloc tubulo-interstitial/ glomerular (PPH4 = 99.5% / 99.8%); experimental link to kidney function unknown
* RRAGD*	rs854922 (0.092, 90.5%), 5’ UTR (18.0)	Experimental link to kidney function unknown.
* SLC6A13*	rs10774020 (0.34,51.9%), eQTL tubulo-interstitial; rs11062102 (0.34, 47.5%), eQTL tubulo-interstitial	New coloc tubulo-interstitial (PPH4 = 99.5%), cell-type specific expression in proximal tubulus in both datasets; link to kidney function unclear
* TFDP2*	rs143710547 (0.08, 58.7%), eQTL tubulo-interstitial	No coloc; experimental link to kidney function unknown
* TPPP*	rs434215 (0.28, 93.2%), eQTL tubulo-interstitial	New coloc tubulo-interstitial (PPH4 = 99.6%); experimental link to kidney function unknown
* YY1AP1*	rs4971092 (0.88, 83.1%), eQTL tubulo-interstitial	New coloc tubulo-interstitial (PPH4 = 99.3%); link to rare Mendelian disease with potential kidney involvement
* ZC3HC1*	rs11556924 (0.38, 84.1%), p.Arg363His (27.5)	Experimental link to kidney function unknown
Novel locus		
* AMPD1*	rs17602729 (0.13, 96.0%), p.Gln45Ter (36.0)	Experimental link to kidney function unknown
* CPXM1*	rs6084180 (0.80, 82.4%), eQTL glomerulus	Experimental link to kidney function unknown
* HNF1A*	rs1800574 (0.03, 48.4%) p.Ala98Val (22.7)	Two variants with identical PPA; less-frequent variant in Mendelian disorder gene with kidney phenotype, previously associated with urate^35^
* IKZF2*	rs112905092 (0.017, 81.2%) Intron (18.8)	Experimental link to kidney function unknown
* NPHS1*	rs3814995 (0.31, 91.5%) p.Glu117Lys, (25.0)	rs3814995 as common variant in rare Mendelian kidney disorder gene not reported before
* SIGIRR*	rs117739035 (0.037, 65.9%), p.Ser80Tyr (23.5)	Experimental link to kidney function unknown

Here we present details on the 23 genes (among the 32 identified by the GPS approach on eGFRcys/BUN-validated, small set relevant variants, Fig. 4) that showed adequate fine-mapping resolution for the respective protein-relevant or kidney-tissue regulatory-relevant variant (PPA > = 10%) and novel evidence compared to the previous work^7^. A detailed description of the genes can be found in Supplementary Note 3.

There might be different preferences as to the weighting of gene evidence. For example, one may want to search for Mendelian kidney disease genes (OMIM^28^ and/or Groopman et al.^29^) mapping to common or less-frequent variants of any relevance (Supplementary Fig. 9). Optionally, researchers with a special focus on one gene may inquire about the GPS for kidney function association evidence. Our GPS is provided as a gene-by-signal or gene-by-locus view (Supplementary Data 14a, b, respectively) and can be customised by using the sorting, filtering, and weighting options to reflect the specific interests.

### Cell-type and tissue-specific gene expression

**Fig. 5:**
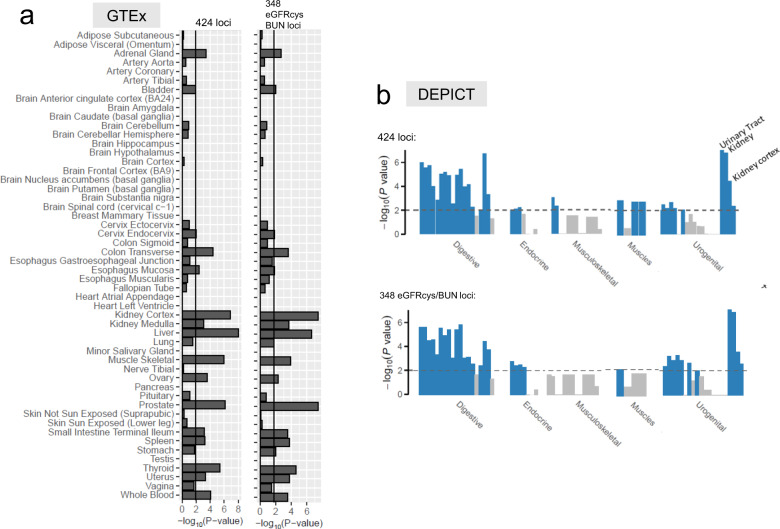
Specific expression in GTEx and DEPICT tissues. Shown are tissue-specific enrichment *P* values from gene expression enrichment analyses. **a** Enrichment analyses in GTEx tissues and cell types (FDR <5%). **b** Tissue- and cell-type-specific enrichment analysis by DEPICT (FDR <5%). Both analyses were conducted twice: based on all 5906 genes located at the 424 identified eGFRcrea loci and based on the subset of 4941 genes located at the 348 eGFRcys/BUN-validated loci. The enrichment in muscle tissue is attenuated after focusing on eGFRcys/BUN-validated loci in both approaches. Significance lines approximately refer to a FDR of 5%. *P* values are derived from a one-sided resampling based enrichment test (“Methods”).

Next, we were interested in the target tissues and cell types of the 5906 candidate genes underneath the 424 identified loci. Using LDSC-SEG^37^, we evaluated whether each gene was specifically expressed (i.e. among the upper 10% of expressed genes) in relevant tissues from GTEx (“Methods”). We observed a significant enrichment of expression effects in 16 GTEx tissues including kidney and muscle (FDR <5%, Fig. 5a, Supplementary Data 17). When reducing the list of candidate genes to the 4941 genes located at the 348 eGFRcys/BUN-validated loci, the enrichment in kidney tissue improved, while the previously observed enrichment in muscle tissue was substantially attenuated (Fig. 5a). A consistent pattern was observed in tissue-specific enrichment analyses with independent expression data by DEPICT^38^ (Fig. 5b, Supplementary Note 4 and Supplementary Data 18, 19). This illustrates the effectiveness of the eGFRcys/BUN-validation to help dissect eGFRcrea loci into those with relevance to kidney function and those with an impact on muscle-based creatinine metabolisms.

**Fig. 6:**
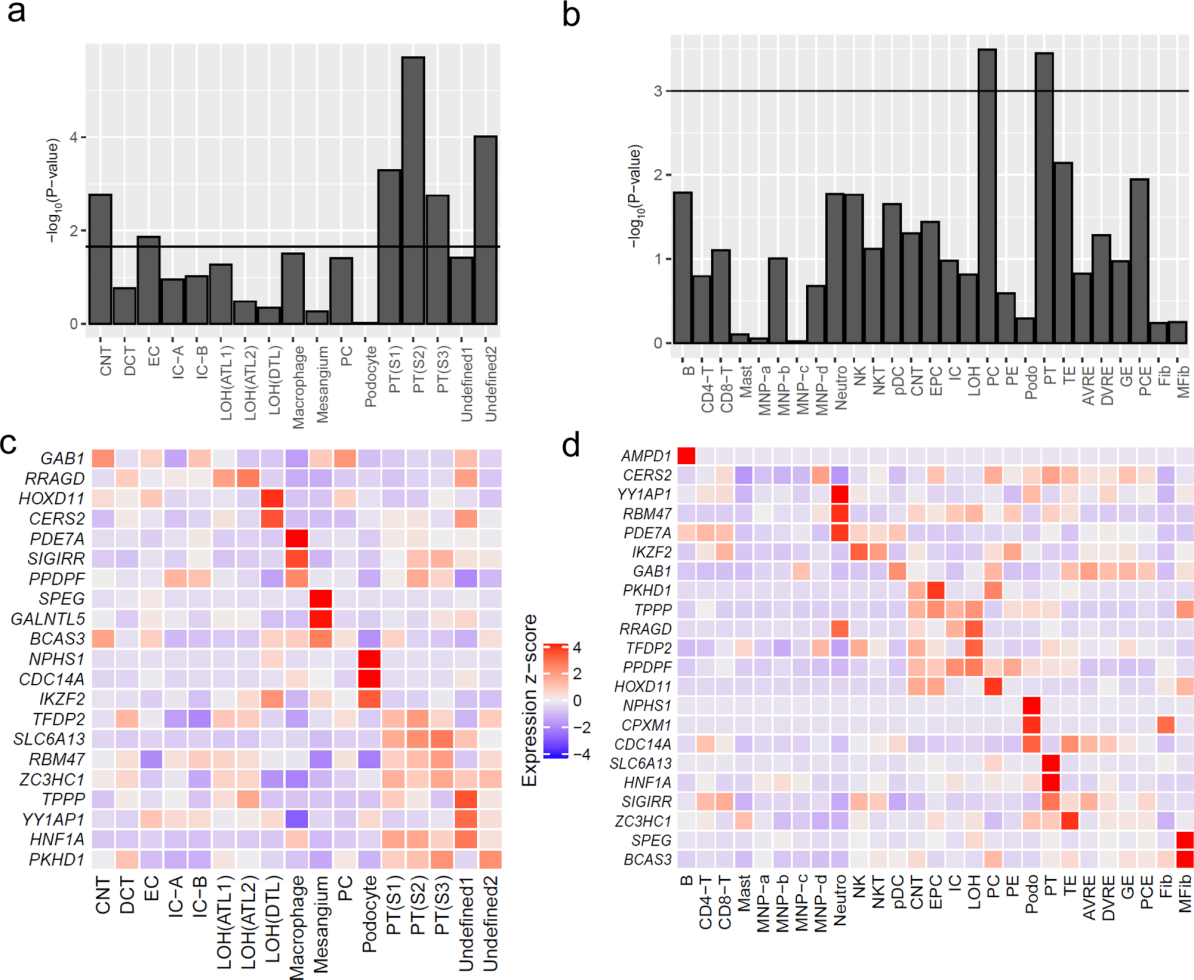
Specific expression in single-cell RNA-seq datasets of the human mature kidney. Shown are results from gene expression enrichment analyses (based on 4941 genes located at eGFRcys/BUN-validated loci) and heatmaps of expression *z* scores for the 23 genes highlighted by Table 2. **a** Enrichment in 17 cell types by Wu et al. **b** Enrichment in 27 cell types by Stewart et al. *P* values are derived from a one-sided resampling based enrichment test (“Methods”). **c** Expression heatmap for 21 of the 23 genes in cell types by Wu et al. (*AMPD1* and *CPXM1* not specifically expressed in any cell type by Wu et al., Supplementary Data 20). **d** Expression heatmap for 22 of the 23 genes in cell types by Stewart et al. (*GALNTL5* not specifically expressed in any cell type by Stewart et al., Supplementary Data 20). In A and B, shown are the enrichment *P* values and significance lines approximately refer to a FDR of 5%. AVRE ascending vasa recta endothelium, B B cell, CD4-T CD4 T cell, CNT connecting tubule, DVRE descending vasa recta endothelium, DCT distal convoluted tubule, EC endothelial cells, EPC epithelial progenitor cell, Fib fibroblast, GE glomerular endothelium, IC intercalated cells, LOH Loop of Henle (ATL ascending thin limbs, DTL descending thin limbs), Mast mast cell, MNP mononuclear phagocyte, MFib myofibroblast, NK natural killer cell, Neutro neutrophil, PCE peritubular capillary endothelium, Podo podocytes, PC principal cells, PT proximal tubule, TE transitional epithelium of ureter.

We then applied LDSC-SEG^37^ to evaluate whether the 4941 genes located at eGFRcys/BUN-validated loci were specifically expressed in two independent single-cell RNA-seq datasets of human mature kidney^39,40^ (Supplementary Data 20). We observed significant enrichment of expression effects (FDR <5%) for three proximal tubule clusters, connecting tubule and endothelial cells in data by Wu et al.^40^ and for proximal tubule and principal cells in data by Stewart et al.^39^ (Fig. 6a, b and Supplementary Data 17). Of particular interest were the 23 highlighted genes (from Table 2). We found all of these specifically expressed in at least one cell type (Fig. 6c, d). Particularly convincing observations made in both independent expression datasets include expression of *NPHS1* and *CDC14A* in podocytes and *HNF1A* and *SLC6A13* and in the proximal tubule. The latter is consistent with our observation of significant *SLC6A13* eQTLs in tubulo-interstitial tissue (Supplementary Data 10).

In summary, our cell-type and tissue-specific expression analyses provided further insights into potential target cells and illustrate the effectiveness of eGFRcys and BUN to help validate eGFRcrea loci with regard to kidney function.

### Locus-based colocalization and a comparison with variant-based eQTL analysis

In the GPS, we analysed each credible set variant for association with gene expression using an FDR approach. An alternative approach is colocalization analysis comparing the eGFRcrea signal with the expression signal^41^. To compare the results of these two related approaches, we conducted colocalization analyses of eGFRcrea association and gene expression, focusing on expression in tubulo-interstitial and glomerular tissue from NEPTUNE using “gtx” (“Methods“). For the 634 signals, we found 59 and 21 colocalizations of eGFRcrea association signals with gene expression in tubulo-interstitial or glomerulus, respectively (posterior probability of “positive” colocalization, PP_H4_ ≥80%, Supplementary Data 21).

The variant-based FDR approach and the signal-based colocalization mostly provided similar results, particularly for small credible sets (≤5 variants, Supplementary Fig. 10). However, we also found examples for discordant results. For example in the *UMOD/PDILT* locus, we observed a positive colocalization (PP_H4_ >0.80) of *UMOD* expression in tubule-interstitial = 0.81 for signal “k2.2”, lead variant rs34882080, fine-mapping PPA = 0.38, set size = 4, Supplementary Data 21). Yet, none of the four credible variants displayed a significant effect on UMOD expression in tubule-interstitial tissue (FDR >5%). We barely missed the 80% colocalization threshold for the primary eGFRcrea signal (PP_H4_ = 0.69 for locus “k2.1”, lead variant rs77924615 with fine-mapping PPA = 1), for which positive colocalization with gene expression was reported previously^7^.

Among the 23 highlighted genes, 7 mapped to small credible sets containing NEPTUNE kidney-tissue eQTLs (Table 2). Of these seven genes, five showed a positive colocalization (PP_H4_≥80%) in the respective NEPTUNE tissue (*GALNTL5, PPDPF, SLC6A13, TPPP* and *YY1AP1* in tubulo-interstitial*; PPDPF* in the glomerulus, Table 3).

**Table 3:** Colocalization analysis results for selected genes.

							Gene expression			eGFRcrea association		
Locus ID	Signal ID	Gene	PP_H4	rsid	EA	OA	BETA	*P*	FDR	BETA	*P*	PPA
Expression in tubule-interstitial tissue												
k4.1	1	*GALNTL5*	**0.987**	rs10224210	C	T	0.67	1.0E-05	**0.011**	−0.0078	2.9E-139	0.50
k21.2	2	*TFDP2*	0.001	rs58436159	T	C	−0.58	1.1E-05	**0.012**	−0.0051	1.6E-23	0.13
k27.1	1	*SLC6A13*	**0.995**	rs11062102	C	T	−0.29	3.4E-07	**6.6E-04**	−0.0041	2.3E-47	0.47
k88	1	*TPPP*	**0.996**	rs434215	A	G	0.57	7.4E-06	**0.0087**	−0.0039	6.4E-26	0.93
k99.1	1	*PPDPF*	**0.995**	rs2314639	T	C	−0.47	1.0E-10	**4.6E-07**	−0.0035	5.0E-17	0.07
k191.2	2	*YY1AP1*	**0.993**	rs4971092	T	C	−0.73	2.9E-05	**0.025**	−0.0027	1.0E-10	0.83
n95.1	1	*CPXM1*	0.158	rs6084184	A	G	−0.43	5.1E-04	0.19	−0.0019	2.0E-08	0.07
Expression in glomerular tissue												
k4.1	1	*GALNTL5*	0.033	rs10224210	C	T	0.15	0.44	0.99	−0.0078	2.9E-139	0.50
k21.2	2	*TFDP2*	0.035	rs2203002	T	C	0.22	0.0976	0.94	−0.0051	1.2E-23	0.14
k27.1	1	*SLC6A13*	0.039	rs11062102	C	T	−0.11	0.12	0.95	−0.0041	2.3E-47	0.47
k88	1	*TPPP*	0.043	rs434215	A	G	0.19	0.21	0.97	−0.0039	6.4E-26	0.93
k99.1	1	*PPDPF*	**0.998**	rs72629024	G	C	−0.46	4.9E-07	**0.0023**	−0.0036	3.5E-18	0.85
k191.2	2	*YY1AP1*	0.164	rs4971092	T	C	0.30	0.037	0.90	−0.0027	1.0E-10	0.83
n95.1	1	*CPXM1*	0.731	rs6084180	T	C	−0.87	1.9E-09	**1.8E-05**	−0.002	1.3E-08	0.82

For the seven genes with small 99% credible sets (<= 5 variants, among 23 highlighted genes from Table 2) that contain significant eQTLs in kidney tissue, we here show results from colocalization analysis between eGFRcrea association signals (*n* = 1,004,040) and gene expression signals for two types of kidney tissues from NEPTUNE (tubule-interstitial and glomerular tissue, *n* = 187). PP_H4 is the posterior probability of positive colocalization^41^. We also show the respective credible set variant with the smallest *P* value for gene expression and its association estimates for gene expression (NEPTUNE data) and eGFRcrea (GWAS data) (EA: effect allele, OA: other allele, BETA: genetic effect per EA, P: two-sided association *P* value based on Wald test, FDR: false-discovery-rate, PPA: posterior probability of association from variant-based fine-mapping). Locus/Signal ID: Identifier of identified locus/signal (“n” novel, “k” known; first integer indicating the locus, second integer the signal within the locus). Marked in bold are positive colocalizations (PP_H4 ≥80%) and significant eQTLs (FDR <5%).

In summary, colocalization analyses show supportive results for many eQTL-findings among credible set variants in precisely the same kidney tissue, but not for all.

### Aggregated genetic impact on eGFRcrea

To quantify the overall genetic impact on eGFRcrea, we applied different approaches (“Methods”). First, using LD-score regression (LDSC)^42^ in UKB data (unrelated individuals, European ancestry, *n* = 361,674), we estimated the additive contribution of all 1,167,355 variants with European reference LD scores (“Methods”), i.e., narrow-sense heritability, h^2^, at 13.4%. Second, LD-score regression analysis applied to cell-type-specific expressed genes (LDSC-SEG, “Methods”)^37^ showed that eGFRcrea genetic heritability was significantly enriched (FDR<5%) in three proximal tubule clusters, principal cells and connecting tubule in expression data by Wu et al.^40^ and Stewart et al.^39^ (up to twofold enrichment; 2 proximal tubule clusters reported previously based on Wu et al.^40,43^, Supplementary Data 22). Third, using summary statistics of the independent second meta-analysis on eGFRcrea (three studies, total *n* = 417,288, in case of multiple signals per locus conditioning via GCTA^24^), we estimated that 9.8% of the eGFRcrea variance was explained by the 634 independent signal-index variants with 8.1% by 408 signals at known loci and 1.7% by 226 signals at novel loci (assuming phenotype variance from the ARIC study, Table 4a, Supplementary Data 4, “Methods”). This compares to 7.1% estimated previously^7^ (also with ARIC study as reference). We also found that the explained variance was larger in population-based studies as compared to hospital-based studies (assuming phenotype variance from the respective study, Table 4a).

**Table 4:** Explained variance and genetic risk score analyses.

(a)					
Study	*N*	Study design	Number of variants	sd of age-/sex-adjusted log eGFRcrea in the respective study	*R*^2^
UKB	436,581	Population-based	634	0.15	9.3%
HUNT	69,389	Population-based	625	0.15	6.7%
MGI	47,219	Hospital-based	620	0.28	3.7%
MVP	300,680	Hospital-based	620	0.28	4.1%
Second meta	417,288	Meta-analysis	632	0.13^a^	9.8%
				0.28^b^	2.0%

(b)						
Study	*N*	GRS	b_GRS_ per sd_GRS_	Mean eGFRcrea difference for 95th vs 5th percentile of GRS	P_GRS_	*R*^2^
HUNT	26,254	Unweighted	−2.62 ml/min/1.73 m^2^	−8.6 ml/min/1.73 m^2^	1.5E-282	4.8%
		Weighted	−2.88 ml/min/1.73 m^2^	−9.5 ml/min/1.73 m^2^	6.3E-344	5.8%
AugUR	1105	Unweighted	−2.49 ml/min/1.73 m^2^	−8.2 ml/min/1.73 m^2^	1.2E-08	2.9%
		Weighted	−2.98 ml/min/1.73 m^2^	−9.8 ml/min/1.73 m^2^	8.8E-12	4.2%

^a^From population-based ARIC (as in Wuttke et al.^7^).

^b^From hospital-based MVP.

Shown are results from the explained variance and genetic risk score (GRS) analysis based on the 634 identified signal index variants. (a) Summary of explained variance analyses based on summary statistics from population- and hospital-based studies or from the second meta-analysis. UKB was part of the primary identifying meta-analysis. HUNT, MVP and MGI were independent studies, which were meta-analysed as second meta-analysis (*n* = 417,288). The variance explained by the 634 signal lead variants (*R*^2^) was computed based on genetic effects, genotype and phenotype variance from the respective study. Since phenotype variance was not available for the second meta-analysis, we here assumed phenotype variances taken from the population-based study ARIC or from the hospital-based study MVP. (b) Summary of GRS analyses. For the GRS, two example studies of different age range were analysed, each independent from the identifying GWAS meta-analysis: HUNT (*n* = 26,254, population-based, age 19–99 years, sd of age-/sex-adjusted eGFRcrea = 11.9 ml/min/1.73 m^2^) and AugUR (*n* = 1105, population-based of mobile elderly, age 70–95 years, sd of age-/sex-adjusted eGFRcrea = 14.6 ml/min/1.73 m^2^). The unweighted and weighted GRS (i.e., using effect sizes from eGFRcrea GWAS) were computed and the association of the GRS on eGFRcrea (not log-transformed) and the variance explained (*R*^2^) were derived via linear regression with GRS as covariate and eGFRcrea as outcome (adjusted for age, sex and principal components, “Methods“; GRS and eGFRcrea descriptives in Supplementary Table 1).

We also estimated the explained variance via a genetic risk score (GRS) across the 634 variants in two population-based studies that captured different age ranges and were independent of the identifying GWAS meta-analysis (general adults from HUNT^44^: age 19–99y, *n* = 26,254; elder adults from AugUR^45^: age 70–95 y, *n* = 1105; unrelated, European ancestry; “Methods”). The weighted GRS explained more phenotypic variance in HUNT than in AugUR (6% versus 4%), which can be explained by the smaller phenotypic variance in HUNT than AugUR (Table 4b and Supplementary Table 1). The explained variance in HUNT was still smaller than the estimate from the GCTA-based use of summary statistics where the ARIC study was used as a reference to be able to compare the respective estimate with previous work. In part, this might be explained by the smaller phenotype variance in ARIC versus HUNT. While the estimates of explained variance differed between studies and approaches, the association estimates of the GRS on eGFRcrea were very stable between the two studies: we observed significant and similar GRS effects per standard deviation and an average difference in eGFRcrea between the 95th and 5th percentile of −8.6 to −9.8 ml/min/1.73 m^2^ (Table 4b).

In summary, we found increased genetically explained eGFRcrea variance compared to previous work^7^, enriched heritability in specific kidney cell types, and a difference of 9 to 10 ml/min/1.73 m^2^ when comparing an unfavourable with a favourable genetic profile in two independent population-based studies.

## Discussion

Our study demonstrates the impact of increased sample size not only on GWAS findings for eGFRcrea, but also on substantially improved fine-mapping and alternative biomarker support. We conducted GWAS on eGFRcrea >1.2 million and fine-mapping in >1.0 million individuals of European ancestry and we here introduced large genetic analyses on eGFRcys in >450,000 to help derive alternative biomarker support additionally to >850,000 with BUN assessment. By this, we nearly doubled the number of significantly associated eGFRcrea loci to 424 and more than doubled the number of loci with eGFRcys/BUN-support to 348 compared to the previous work^7^. Among the 634 independent signals, we successfully resolved the fine-mapping resolution to one variant for 44 and to a small credible set for 138 signals, which compares to 20 and 58 such signals previously^7^. We found almost 10% of eGFRcrea variance explained by the 634 signal-lead variants, compared to 7.1% previously^7^, and average eGFRcrea was lowered by 9 to 10 ml/min/1.73 m^2^ when comparing individuals with an unfavourable versus a favourable genetic risk profile. We aggregated comprehensive and systematic in silico follow-up results for the more than 5000 genes and 38,306 credible set variants underneath the identified loci into a GPS tool to navigate through the abundance of evidence.

One challenge for kidney function genetics is the dissection of eGFRcrea loci likely related to kidney function from those related to creatinine metabolism. For this, we used genetic data on eGFRcys and BUN to assess the consistency of effects. Kidney function assessment by eGFRcys is superior to eGFRcrea in predicting morbidity and mortality^46^, but cystatin C measurement is expensive and less available in large epidemiological studies. The previously largest eGFRcrea GWAS by Wuttke and colleagues^7^ had not used eGFRcys for this reason but focused on BUN to seek support for eGFRcrea associations despite known limitations^47^. Utilising the recent release of cystatin C measurements in UKB enabled an eGFRcys sample sizes of >450,000 and the support of 75% of the 424 eGFRcrea loci. Importantly, our tissue enrichment analyses restricted to the 348 loci with eGFRcys- and/or BUN-support sharpened the finding on kidney tissue and substantially reduced enrichment in muscle tissue. Our results suggest that future work on kidney function genetics may benefit from even larger eGFRcys data, possibly integrated with more advanced decomposition or clustering algorithms^48,49^, to help resolve the classification of eGFRcrea loci as kidney function relevant.

A hallmark of effective fine-mapping are signals with small credible sets of variants, as these credible sets contain the statistically most likely causal variant^17^ (assuming there is one causal variant and this is among those analysed). Therefore, small credible sets provide a practical starting point for variant prioritisation. We obtained a fine-mapping resolution down to five variants for 138 of the 634 independent signals including 118 narrowed down signals in novel loci or newly small in known loci. Particularly certain evidence derives from the 44 signals resolved down to one variant, which immediately suggest the causal variant with 99% probability, and 30 of these 44 single-variant sets were identified here for the first time, i.e. resided in a novel locus or have not been resolved down to one variant previously^7^.

Selecting relevant genes for functional follow-up is a challenging task in the interpretation of GWAS results. The mapping of a gene to a protein- or regulatory-relevant variant that is likely causal for the association with kidney function renders this gene a likely causal gene with the suggested mechanism implied by the respective variant. Our novel loci and signals newly narrowed down to a small credible set suggest 23 such genes (Table 2). These genes provide new ideas or certainty for human association validated targets and thus compelling starting points for experimental studies. Some of these genes are known for rare Mendelian kidney disease where now a new common or less-frequent variant is implicated for affecting general kidney function (*PKDH1* with new certainty, *NPHS1* and *HNF1A* in novel loci), a phenomenon observed also in other contexts, like *MC4R* for obesity^50^. Beyond this specific search for relevant genes as conducted here, our GPS is a comprehensive and customisable tool that can be queried for different research questions and personal preference. In our GPS, we focus on variants with high relevance for the protein (CADD score ≥15) or *cis*-regulatory variants mapping to genes in the same locus region (defined as +/−250 kb beyond the genome-wide significant association signal). We also provide summary statistics genome-wide for association with eGFRcrea, eGFRcys, and BUN as an important resource for even larger future GWAS. These summary statistics, as our GWAS, focus on single-nucleotide polymorphisms and disregard structural variation, insertions, and deletions as well as pleiotropic effects.

The necessity of replicating GWAS findings has recently been revisited in light of the general lack of suitable and appropriately powered replication samples^23^. Still, all 424 lead variants showed directionally consistent, nominally significant associations when analysing UKB and CKDGen separately. Furthermore, we gathered independent data on 400,000 individuals for a second meta-analysis on eGFRcrea. Despite this large sample size, our power computations showed that this was not sufficient for a formal replication. Still, 361 of the 424 locus lead variants’ associations were supported by this second meta-analysis. Together, this supports our confidence in these associations being genuine and the GPS provides an option to focus on loci with independent second evidence when limiting false positives is a primary concern. Our GWAS is limited in its number of individuals from ancestries other than European, which had us limit our fine-mapping to European ancestry. Future studies augmenting on non-European ancestry individuals are warranted to provide equally powered association analysis and fine-mapping for all ethnicities^51,52^.

In summary, our results help guide functional follow-up studies on various ends: (i) the novel identified loci generate new biological hypotheses, (ii) the improved fine-mapping resolution in known loci increases the certainty in the relevant target, (iii) the support by eGFRcys/BUN association enhances the certainty that the identified eGFRcrea association is related to kidney function, (iv) 23 genes with compelling evidence provide human association validated targets and immediate starting points, and (v) our searchable and customisable GPS table provides a powerful tool to support the cross-talk between GWAS researchers and molecular biology scientists.

## Methods

### Data analyses overview

Our data analyses had three major steps: (1) GWAS for eGFRcrea, (2) support for identified eGFRcrea loci by alternative biomarkers via BUN and eGFRcys, (3) fine-mapping of identified eGFRcrea loci and bioinformatic follow-up. For the GWAS on eGFRcrea, we included two sources of data in our primary meta-analysis (*n* = 1,201,909): (i) GWAS summary statistics for eGFRcrea from the CKDGen consortium (*n* = 765,348, predominantly European ancestry)^7^ and (ii) GWAS results generated in this work for eGFRcrea in UKB (application number 20272, *n* = 436,561, European ancestry)^18^. We focused on European ancestry in UKB, because this was the by-far largest ethnicity subset of UKB with other non-European ethnicities being clearly underrepresented and diverse^18^. We also conducted eGFRcrea meta-analyses focusing on European ancestry individuals for CKDGen and UKB (total *n* = 1,004,040). Summary statistics for CKDGen for ancestries other than European had not been made available, since these groups had been considered too small for interpretation. For independent evidence on GWAS-identified eGFRcrea loci, we conducted a second meta-analysis comprising 417,288 individuals of European ancestry from MVP (*n* = 300,680, hospital-based), MGI (*n* = 47,219, hospital-based) and HUNT (*n* = 69,389, population-based). For the alternative biomarker support, we conducted analyses in UKB and meta-analysed these results with CKDGen association results for eGFRcys and BUN (*n* = 460,826 and 852,678, respectively). Details on the phenotypes, downloaded data, association analyses, quality control, meta-analyses and further follow-up analyses are described in detail in the following. Extended acknowledgements for MVP can be found in Supplementary Note 5.

### Phenotypes

The primary outcome of our GWAS meta-analysis is log-transformed eGFRcrea. This was used by the studies contributing to the CKDGen meta-analyses and for our UKB association analysis. In UKB, creatinine was measured in serum by enzymatic analysis on a Beckman Coulter AU5800 (UKB data field 30700, http://biobank.ctsu.ox.ac.uk/crystal/field.cgi?id=30700) and GFR was estimated using the Chronic Kidney Disease Epidemiology Collaboration (CKD-EPI) formula^53,54^. For all studies involved in the CKDGen analysis, creatinine concentrations were measured in serum and GFR was estimated based on the CKD-EPI (for individuals >18 years of age)^53,54^ or the Schwartz (for individuals <= 18 years of age)^55^ formula. Details on the study-specific measurements for the CKDGen studies were described previously^7^. For all studies, eGFRcrea was winsorized at 15 or 200 ml/min/1.73 m^2^ and winsorized eGFRcrea values were log-transformed using a natural logarithm. Secondary outcomes used for downstream analyses include log-transformed eGFRcys and log-transformed BUN. In UKB, cystatin C was measured based on latex enhanced immunoturbidimetric analysis on a Siemens ADVIA 1800 (UKB data field 30720, http://biobank.ctsu.ox.ac.uk/crystal/field.cgi?id=30720) and blood urea was measured by GLDH, kinetic analysis on a Beckman Coulter AU5800 (UKB data field 30670, http://biobank.ctsu.ox.ac.uk/crystal/field.cgi?id=30670). Details on the cystatin C and blood urea measurements in CKDGen studies can be found in the previous work^7,13^. In CKDGen and UKB, eGFRcys was obtained from cystatin C measurements using the formula by Stevens et al.^56^ or the CKD-EPI formula^53,54^, respectively. In all studies, eGFRcys was winsorized at 15 or 200 ml/min/1.73 m^2^ and winsorized eGFRcys values were log-transformed using a natural logarithm. Blood urea measurements in mg/dL were multiplied by 2.8 to obtain BUN values, which were then log-transformed using a natural logarithm.

### GWAS data from CKDGen

Each study in CKDGen had conducted GWAS for eGFRcrea adjusting for age, sex and other study-specific covariates. Summary statistics of each study were GC-corrected. Details on study-specific analysis are described elsewhere^7^. For our primary meta-analysis, we downloaded GWAS summary statistics for eGFRcrea from the CKDGen meta-analysis (https://CKDGen.imbi.uni-freiburg.de/files/Wuttke2019/20171016_MW_eGFR_overall_ALL_nstud61.dbgap.txt.gz, *n* = 765,348)^7^ including 121 study-specific GWAS results comprising 567,460 Europeans, 165,726 East Asians, 13,842 African-Americans, 13,359 South-Asians and 4,961 Hispanics. For our downstream analyses, we also downloaded GWAS summary statistics for eGFRcrea from the CKDGen European-ancestry meta-analysis (https://CKDGen.imbi.uni-freiburg.de/files/Wuttke2019/20171017_MW_eGFR_overall_EA_nstud42.dbgap.txt.gz, *n* = 567,460)^7^, for eGFRcys from a European meta-analysis (https://CKDGen.imbi.uni-freiburg.de/files/Gorski2017/CKDGen_1000Genomes_DiscoveryMeta_eGFRcys_overall.csv.gz, *n* = 24,061)^13^ and for BUN from a meta-analysis in predominantly European ancestry individuals as reported previously^7^ (https://CKDGen.imbi.uni-freiburg.de/files/Wuttke2019/BUN_overall_ALL_YL_20171017_METAL1_nstud_33.dbgap.txt.gz, *n* = 416,178). Most studies included in CKDGen meta-analyses were population-based. All studies used an additive genotype model and imputed the genotyped variant panel to the Haplotype Reference Consortium (HRC, v1.1)^20^ or the 1000 Genomes Project (ALL panel)^22^ reference panels. Details on the meta-analysis methods were described previously^7,13^.

### GWAS data from UK Biobank

We conducted linear mixed model GWAS for log(eGFRcrea), log(eGFRcys) and log(BUN) in UKB using the fastGWA tool^19^. We included age, age^2^, sex, age × sex, age^2^ × sex, and 20 principal components as covariates in the association analyses as recommended by the developers^19^. The UKB GWAS were based on additively modelled genotypes that were imputed to HRC^20^ and the UK10K haplotype reference panels^21^. Details on the UKB genotypic resource are described elsewhere^18^. We included individuals of European ancestry, i.e. self-reported their ethnic background as “White”, “British”, “Irish” or “Any other white background” (UKB data field 21000, http://biobank.ctsu.ox.ac.uk/crystal/field.cgi?id=21000). The sample sizes of the UKB GWAS were *n* = 436,581 for eGFRcrea, *n* = 436,765 for eGFRcys and *n* = 436,500 for BUN. Descriptive phenotype statistics for UKB are presented in Supplementary Data 1.

### Quality control

Prior to the meta-analysis, we applied a quality control (QC) procedure to the UKB and CKDGen GWAS results using EasyQC^57^. We utilised the “CREATECPAID” function to create unique variant identifiers that consisted of chromosomal, base position (hg19) and allele codes (i.e. “cpaid”, e.g. “3:12345:A_C”, allele codes in ASCII ascending order). For UKB, we excluded variants with a low-imputation quality (Info <0.6) as done in the previous CKDGen analyses^7^. For both datasets, UKB and CKDGen, we excluded very rare variants with MAF <0.1%; all variants, particularly rare variants, were specifically inspected with regard to imputation quality when they were selected lead variants. Finally, we excluded variants that were exclusively available in only one of the two datasets in order to limit analyses to variants that are available in UKB and CKDGen. This led to the exclusion of insertions, deletions and structural variants from the UKB GWAS results, since CKDGen focused on SNPs^7^. We corrected our UKB association statistics for population stratification using the genomic control inflation factor (*λ* = 1.41)^58^. We also calculated the genomic control inflation factor for the CKDGen results (*λ* = 1.32) but did not apply the correction because the individual studies contributing to the CKDGen meta-analyses were already GC-corrected (see^7^ for details on the study-specific methods).

### Meta-analyses

We conducted fixed-effect inverse-variance weighted meta-analyses of CKDGen and UKB association results using metal^59^. As a primary meta-analysis, we combined log(eGFRcrea) association results from CKDGen and UKB (*n* = 1,201,909). After meta-analysis, we excluded variants with a low minor allele count (MAC < 400) yielding 13,633,840 variants in our final meta-analysis GWAS result for eGFRcrea. The GC lambda inflation factor of the eGFRcrea meta-analysis results was *λ* = 1.28, and the LD-score regression intercept^42^ was 0.90, which reflects conservative study-specific GC correction and indicates the absence of confounding by population stratification. For downstream follow-up analyses, we combined association results from CKDGen European-ancestry individuals with results from UKB individuals for log(eGFRcrea), as well as CKDGen and UKB results for log(eGFRcys) and log(BUN) (*n* = 460,826 and 852,678, respectively).

### Locus definition and variant selection

A variant was defined as genome-wide significant (GWS), when *P* < 5 × 10^−8^. We defined locus borders by adding +/− 250 kb to the first and last GWS variant of a specific region. To achieve independent loci, we selected the variant with the smallest association *P* value genome-wide as a starting point and defined this variant as the lead variant for its locus. Starting at the outermost two GWS variants (*P* < 5 × 10^−8^) in a 1-Mb region centred on the lead variant, areas of another 500kb were checked for GWS variants. If GWS variants were found in this extended region, the region extension step was repeated on the novel outermost GWS variants until no further GWS variants were found. The positions of the two last-found GWS variant in both directions −/+ 250 kb were defined as the locus limits. The locus variants were omitted from the data and the whole process was repeated until no GWS variants remained genome-wide. We defined a locus as novel when none of the 264 known loci discovered by Wuttke et al.^7^ overlapped with our GWS variants. We used the so-defined locus regions (GWS variants +/−250 kb cis window) for the in silico follow-up analyses and defined the genes that overlapped these locus regions as candidate genes.

### Second eGFRcrea meta-analyses in data independent of the GWAS

We evaluated the variants identified for log(eGFRcrea) in the GWAS in independent data. For this, we collected log(eGFRcrea) association estimates for the identified variants from MVP (*n* = 300,680, hospital-based), MGI (*n* = 47,219, hospital-based) and HUNT (*n* = 69,389, population-based) totalling *n* = 417,288 for the second meta-analysis and all of these were from European ancestry. Details on study-specific phenotyping, genotyping and GWAS as well as descriptive phenotype statistics are shown in Supplementary Data 1. We applied QC checks to confirm allele directions and harmonised marker identifiers to “cpaid” using EasyQC^57^. We then conducted fixed-effect inverse-variance weighted meta-analyses of the three studies using metal^59^. We judged a variant as independently associated with eGFRcrea, when the association was nominally significant (*P* < 0.05) and directionally consistent to the primary GWAS result.

### Validation for kidney function based on eGFRcys and BUN

To evaluate the eGFRcrea-associated lead variants for their potential relevance for kidney function, we analysed their genetic association with log(eGFRcys) and log(BUN). Consistency of the eGFRcrea association for a given effect allele with eGFRcys- or BUN association was defined as a nominal significant association (*P* < 0.05) and concordant effect direction for eGFRcys or opposite effect direction for BUN.

### Approximate conditional analyses using GCTA

To identify independent secondary signals at the identified loci, we conducted approximate conditional analyses based on European-only meta-analysis summary statistics using GCTA^24^. The analysis was limited to European-ancestry results due to the lack of an appropriate LD reference panel that reflected the ethnicities in our primary meta-analysis of CKDGen and UKB and due to the fact that European ancestry was, by far, the largest ancestry group in our data. We created a LD reference panel based on 20 K randomly selected unrelated Europeans from UKB. For each identified locus, we applied a stepwise approach to derive the further signals: (i) we first conditioned on the locus lead variant and then selected the most significant variant across all locus variants in this conditional analysis. (ii) If this selected variant showed a genome-wide significant conditional *P* value (*P*_Cond_ < 5 × 10^−8^), this variant was deemed as an independent signal-lead variant and added to the list of variants to condition on. (iii) The procedure was repeated until no more genome-wide significant variant was identified.

### Credible sets of variants

For each variant in each of the identified signals, we calculated approximate Bayes factor (ABF) and PPA using the Wakefield method^25^. We obtained 99% credible variant sets for each independent association signal. We used *W* = 0.005^2^ as prior variance as done previously^7^. PPAs were calculated based on the meta-analysis summary statistics for loci with only one signal and based on conditioned summary statistics for loci with multiple independent signals (each signal conditioning on the other signal-lead variants in the locus). One set of 99% credible variants was obtained for each independent signal. Credible sets with ≤5 variants were defined as “small” and the respective signal as a “signal with high fine-mapping resolution”. We defined a signal as “newly small” in a known locus when the credible set size had been larger in the previous GWAS on eGFRcrea^7^ or the signal has not been fine-mapped before.

### Gene PrioritiSation

To prioritise genes among the list of candidate genes at the discovered eGFRcrea loci, we performed a series of statistical and bioinformatic follow-up analyses based on the secondary signal analysis from the EUR-ancestry meta-analysis. (1) For each credible set variant within a candidate gene, we derived the CADD PHRED-Score^26^ to identify credible set variants with high predicted deleteriousness (CADD ≥15). We chose the threshold of 15, since this represents the 3.2% most deleterious variants of the 8.6 billion variants available in CADD. CADD uses the Ensembl Variant Effect Predictor (VEP)^60^ to obtain gene model annotation and combines this information to 17 possible consequence levels. Based on the CADD internal consequence score (ConsScore), we classified each prioritised variant into three groups: (i) “stop-gained”, “stop-lost”, “non-synonymous” (ConsScore 8 or 7), (ii) “canonical-splice”, “noncoding-change”, “synonymous”, “splice-site” (ConsScore 6 or 5) and (iii) other (ConsScore 4-0). We restricted the application of the CADD information to variants located within genes to avoid major overlap with variants that influence gene expression levels that were analysed in the next steps. (2) We highlighted credible set variants within each locus that were expression quantitative trait loci (eQTL) variants in kidney (and other) tissue for any gene in the respective locus (*cis*-eQLTs). We analysed eQTLs quantified from glomerular and tubule-interstitial tissue in the NEPTUNE study^61^ and from 44 tissues including kidney cortex in the GTEx project^62^ with regard to the significant association (FDR <0.05) on candidate gene expression levels. We used the FDR provided by GTEx and applied a Benjamini–Hochberg FDR correction^63^ to the NEPTUNE association *P* values for glomerular and tubule-interstitial tissue separately (to obtain an FDR for each variant x gene combination). (3) Analogously, we inquired credible variants in a locus for a significant effect (FDR <0.05) on expression levels of exon junctions or variation in the relative abundances of gene transcript isoforms for each gene in the locus (sQTLs), using sQTL summary statistic from the GTEx database^62^. (4) Genes with kidney-relevant phenotypes in mice were selected from the Mouse Genome Informatics (MGI)^27^ hierarchical ontology. All phenotypes subordinate to “abnormal kidney morphology” (MP:0002135) and “abnormal kidney physiology” (MP:0002136) were gradually extracted. A table with all genes occurring in MGI-database and the associated phenotypes was restricted to the kidney-relevant phenotypes and compared to the list of candidate genes. (6) We selected genes known to cause monogenic kidney phenotypes or disease in human based on two resources: the Online Mendelian Inheritance in Man (OMIM) * database^28^ and a recent publication by Groopman et al.^29^. We generated a table of kidney phenotypes and causal genes in the context of human disorders by querying the OMIM database for phenotype entries subordinate to the clinical synopsis class “kidney”. This table was manually curated excluding diseases with “kidney”-phenotype entries being: “normal kidneys”, “normal renal ultrasound at ages 4 and 7 (in two family)”, “no kidney disease”, “no renal disease; normal renal function”, “normal renal function; no kidney disease”, “no renal findings”. We further used a summary table by Groopman et al., which included 625 genes associated with Mendelian forms of kidney and genitourinary disease (http://www.columbiamedicine.org/divisions/gharavi/files/Kidney_Gene_List_625.xlsx). Both tables were combined and checked for concordance with candidate genes.

### Cell-type and tissue-specific enrichment of expression

We were interested in whether the candidate genes were specifically expressed in certain cell types and tissues. We used expression data from 52 GTEx (v8) tissues^64^, 17 human cell types from Wu et al.^40^ and 27 human cell types from Stewart et al.^39^. We applied LDSC-SEG^37^ analyses to obtain the top 10% specifically expressed candidate genes in each cell type. Detailed information on the enrichment analyses has been described previously^65^. In brief, the number of independent variants per gene was computed based on genotypes from the German Chronic Kidney Disease study (GCKD) using PLINK v1.90^66^. We generated a database of 18,215 Entrez gene identifiers using the Bioconductor R database org.Hs.eg.db v3.8.2 that contained, for each gene, the number of independent variants, gene length, as well as membership in the top 10% highly expressed genes in each GTEx (v8) tissue or human cell types from Wu et al.^40^ and or from Stewart et al.^39^. For enrichment testing, the observed number of candidate genes in the top 10% highly expressed genes in each GTEx tissue and cell type was compared to the number obtained from lists of randomly drawn genes that were matched by the number of candidate genes, deciles of gene length and number of independent variants (100 million random draws). Multiple testing correction was performed using the Benjamini–Hochberg FDR approach.

### DEPICT analyses

We conducted DEPICT^38^ analyses of tissue-specific expression enrichment, gene prioritisation and gene set enrichment. We applied DEPICT twice: first, to all significant eGFRcrea loci and, second, restricting to eGFRcys or BUN-validated loci. DEPICT was used with the following settings: association_P-value_cutoff = 5 × 10^−8^, number of_repititions = 50, and number of_permutations = 500. The HLA-region was excluded from all analyses. For these analyses, we utilised the primary GWAS meta-analysis summary statistics for eGFRcrea.

### Colocalization analyses

We were interested in whether our identified eGFRcrea signals co-localised with gene expression signals in tubule-interstitial or glomerular tissue from NEPTUNE^61^. We conducted colocalization analyses using the method described by Giambartolomei et al.^41^. For each signal, colocalization analyses were performed for the respective locus’ genes separately for tubule-interstitial or glomerular tissue. We used the eGFRcrea EUR meta-analysis summary statistics for loci with only one signal and the conditioned summary statistics for loci with multiple independent signals. We used the R package “gtx” and its coloc.compute function with 0.005^2^ as the prior variance for the eGFRcrea association (similar to what was used for the statistical fine-mapping of credible variants) and 0.55^2^ as prior variance for the expression in tubule-interstitial or 0.53^2^ as prior variance for the expression in glomerular tissue. The prior variances for the expression data were obtained from the Wakefield formula (8)^25^, assuming that 95% of significant eQTLs (FDR<0.05) in NEPTUNE fall within the effect size range −1.07 to 1.07 in tubule-interstitial or −1.04 to 1.04 in glomerular tissue.

### Heritability and cell-type-specific enrichment of heritability

We were interested in the general impact of genetics on eGFRcrea. For this, we estimated narrow-sense heritability for log eGFRcrea (i.e. the additive genetic contribution to eGFRcrea for variants throughout the genome) by LD-score regression analyses using LDSC^42^ based on the UKB summary statistics (not GC-corrected, limiting to variants available in the LDSC reference data “w_hm3.snplist”). We were further interested in whether the genetic contribution to eGFRcrea differed between specific cell types. We thus investigated whether the heritability of eGFRcrea was enriched in one of the 17 or 27 cell types from Wu et al.^40^ or Stewart et al.^39^, respectively. The data from Stewart et al.^39^ were independent of the data from Wu et al.^40^ and were additionally analysed here compared to the previous publication^43^. For each cell type, we conducted LDSC^42^ heritability analyses that were restricted to regions surrounding (+/−100 kb to transcribed regions) the 10% most specifically expressed genes within cell type. Details on the cell-type-specific expression and heritability enrichment analyses and for the Wu et al. data^40^ can be found elsewhere^43^. The Stewart et al. dataset including the expression matrix and cell-type annotation was downloaded from http://www.kidneycellatlas.org/.

### Explained variance and genetic risk score analyses

The explained variance was calculated for each of the independent signal-lead variants and then summed up to obtain the variance explained by all identified signal-lead variants. For each variant, we calculated *R*^2^ = b^2^*Var(G)/Var(Y). Here, b is the genetic effect on log(eGFRcrea) from the respective study or from the second independent meta-analysis (for the locus lead variant for loci with only one signal; for the signal-index variant, GCTA-conditioned on other signals in the locus, for loci with multiple signals), Var(G) is the genetic variance calculated from Var(G) = 2*MAF*(1-MAF) and Var(Y) is the phenotypic variance from the respective study or for the second meta-analysis based estimation set to 0.016 (as variance of age- and sex-adjusted log(eGFRcrea) residuals in the population-based study ARIC, 11,827 individuals of European ancestry, as utilised previously^7^) or to 0.078 (as the variance of age- and sex-adjusted log(eGFRcrea) residuals in the hospital-based study MVP, 300,680 individuals of European ancestry). To estimate the cumulative effect of genetic variants on eGFRcrea (not log-transformed), we conducted GRS analyses in unrelated individuals of European ancestry from two studies: The German AugUR study (prospective study in the mobile elderly general population around Regensburg, Germany, age range 70–95 y, mean +/− SD eGFRcrea = 70.0 +/− 15.5 ml/min/1.73 m^2^, *n* = 1,105)^45^, and the Norwegian HUNT study (population-based study, age range 19–99y, mean +/− SD eGFRcrea = 101.1 +/− 18.7 ml/min/1.73 m^2^, *n* = 26,254)^44^. Both studies were independent of the identifying GWAS meta-analysis. To obtain GRS effects that are interpretable as eGFRcrea units, we did not apply a log-transformation to eGFRcrea for the GRS analyses. We calculated an unweighted GRS that is interpretable on the “per allele” scale by adding up the eGFRcrea-decreasing alleles of the identified variants. To account for potential differences in the effect sizes between variants, we also calculated a weighted GRS by adding up the eGFRcrea-decreasing alleles of the signal-index variants, using the genetic effect observed in the identifying GWAS meta-analysis (GCTA-conditioned on other signals in the locus, for loci with multiple signals). We regressed eGFRcrea on the unweighted or the weighted GRS adjusting for age, sex and study-specific PCs (four PCs for AugUR, ten PCs for HUNT). We provided GRS effect sizes per standard deviation of the respective GRS and compare high (95th percentile) versus low (5th percentile) GRS groups.

### Ethics approval and consent to participate

For all studies, study participants obtained informed consent and local ethics committees approved the study protocols.

### Reporting summary

Further information on research design is available in the Nature Research Reporting Summary linked to this article.

## Supplementary information

Supplementary Information

Description of Additional Supplementary Files

Supplementary Data 1

Supplementary Data 2

Supplementary Data 3

Supplementary Data 4

Supplementary Data 5

Supplementary Data 6

Supplementary Data 7

Supplementary Data 8

Supplementary Data 9

Supplementary Data 10

Supplementary Data 11

Supplementary Data 12a

Supplementary Data 12b

Supplementary Data 12c

Supplementary Data 12d

Supplementary Data 13

Supplementary Data 14a

Supplementary Data 14b

Supplementary Data 15

Supplementary Data 16

Supplementary Data 17

Supplementary Data 18

Supplementary Data 19

Supplementary Data 20

Supplementary Data 21

Supplementary Data 22

Reporting Summary

## Data Availability

Summary genetic association results for UKB and the meta-analysis of UKB and CKDGen for log(eGFRcrea), log(eGFRcys) and log(BUN) can be downloaded from www.genepi-regensburg.de/ckd or from https://ckdgen.imbi.uni-freiburg.de/. Previously published association results for eGFRcrea can be found at https://ckdgen.imbi.uni-freiburg.de/. The GPS table is also available from www.genepi-regensburg.de/ckd.
