# Supplementary material for: Discovery and prioritization of variants and genes for kidney function in >1.2 million individuals
Source: Nat Commun. 2021 Jul 16;12:4350. doi: 10.1038/s41467-021-24491-0 (PMC8285412; doi:10.1038/s41467-021-24491-0)
Supplement: Supplementary file 1 — Supplementary Information [file 41467_2021_24491_MOESM1_ESM.pdf]

SUPPLEMENTARY INFO – Stanzick et al.

Contents

SUPPLEMENTARY FIGURES 2

Supplementary Figure 1. Analysis workflow. 2

Supplementary Figure 2. QQ-Plot. 3

Supplementary Figure 3. Comparison of primary and European-only analysis 4

Supplementary Figure 4. Second stage power computation. 5

Supplementary Figure 5. Regional Association Plots (RAPs) 6

Supplementary Figure 6. Regional Association Plots (RAPs) at the *UMOD*/*PDILT*. 17

Supplementary Figure 7. Posterior Probability of Association (PPA) 18

Supplementary Figure 8. Gene PrioritiSation (GPS) based on ALL-ancestry variants. 19

Supplementary Figure 9. Locus based Gene PrioritiSation (GPS) 20

Supplementary Figure 10. Comparison of colocalization and FDR-approach 21

SUPPLEMENTARY NOTE 22

Supplementary Note 1. Power computation for second stage meta-analysis. 22

Supplementary Note 2. 21 genes mapping to small credible sets of size 2-5 variants. 23

Supplementary Note 3. Detailed description of the 23 highlighted genes. 23

Supplementary Note 4. DEPICT gene-set enrichment and gene prioritization 26

Supplementary Note 5. VA Million Veteran Program 27

SUPPLEMENTARY TABLES 31

Supplementary Table 1. Descriptives of genetic risk score (GRS) studies. 31

REFERENCES 32

SUPPLEMENTARY FIGURES

Supplementary Figure 1. Analysis workflow.

General workflow of the analyses with indication of the sample sizes and data used. More details on the specific analyses are presented in the **Methods**. Abbreviations: MAF: minor allele frequency; Info: Imputation quality Info score; MAC: Minor allele count; eGFRcys: glomerular filtration rate estimated from cystatin, BUN: blood urea nitrogen, EUR: European, MVP: Million Veterans Program; MGI: Michigan Genomics Initiative; GCTA: Genome-wide Complex Trait Analysis; CADD: Combined Annotation Dependent Depletion; MGI: Mouse Genome Informatics; OMIM: Online Mendelian Inheritance of Men.

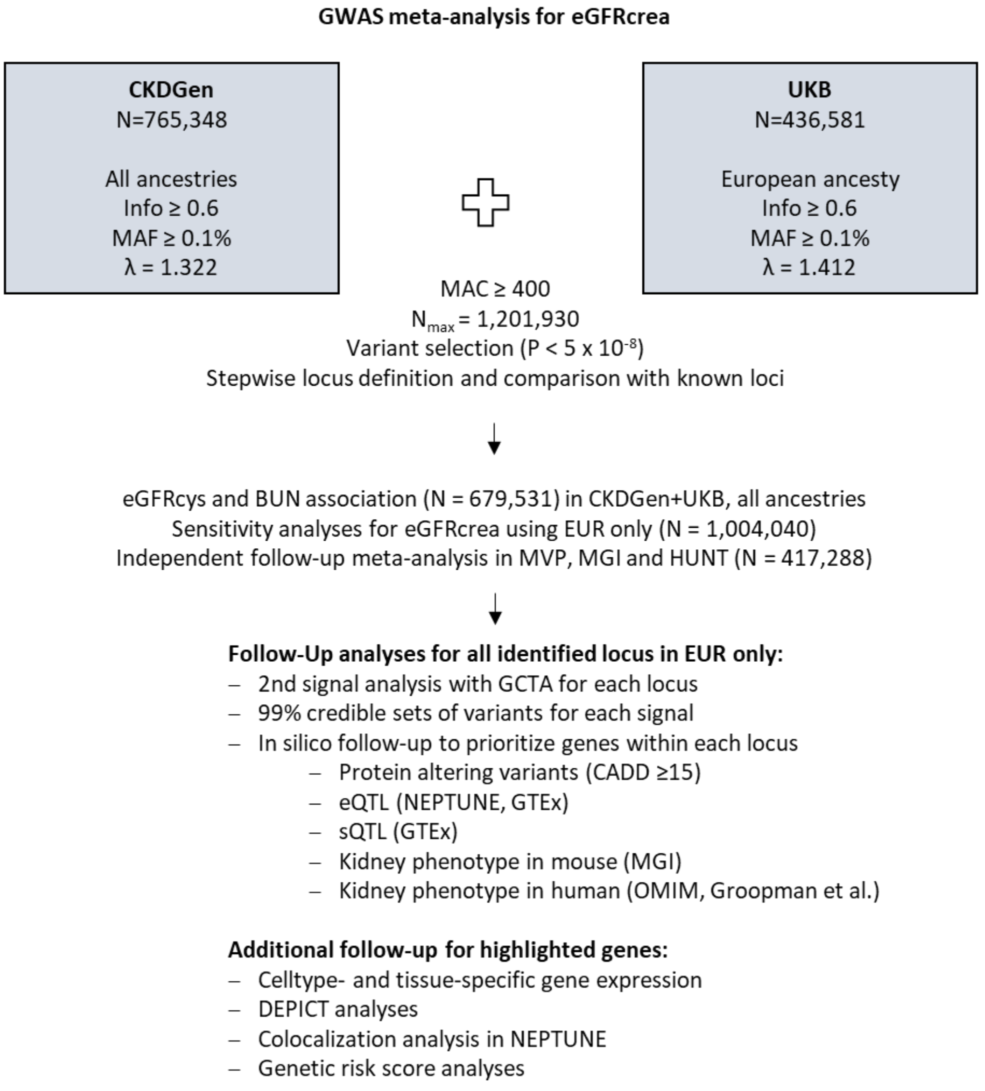

Supplementary Figure 2. QQ-Plot for primary meta-analysis results for eGFRcrea.

The QQ plot compares the expected versus the observed distribution of P-values in the primary meta-analysis for eGFRcrea (n = 1,201,929). Black dots mark association the $-\log_{10}$(P-values) of all variants; red dots show the respective results excluding the 264 loci previously described by Wuttke et al [1].

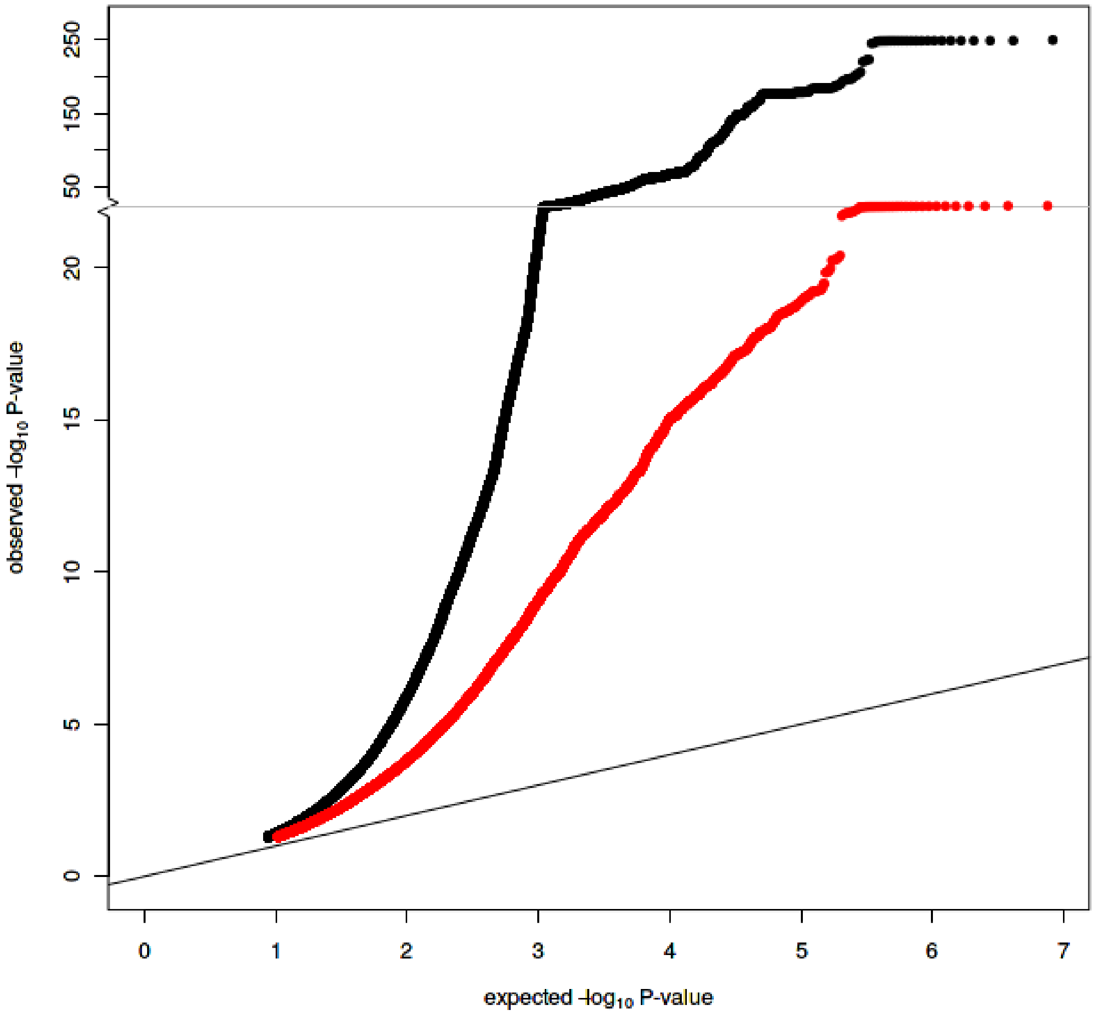

Supplementary Figure 3. Comparison of primary and European-only meta-analysis for eGFRcrea.

For the 424 lead variants identified by the primary meta-analysis (n = 1,201,929, including 197,888 non-European individuals), the scatterplots contrast the genetic effect sizes (aligned to eGFRcrea decreasing alleles) (**Panel A**) and the association P-Values (**Panel B**) between the primary and the European-only meta-analysis (n = 1,004,040).

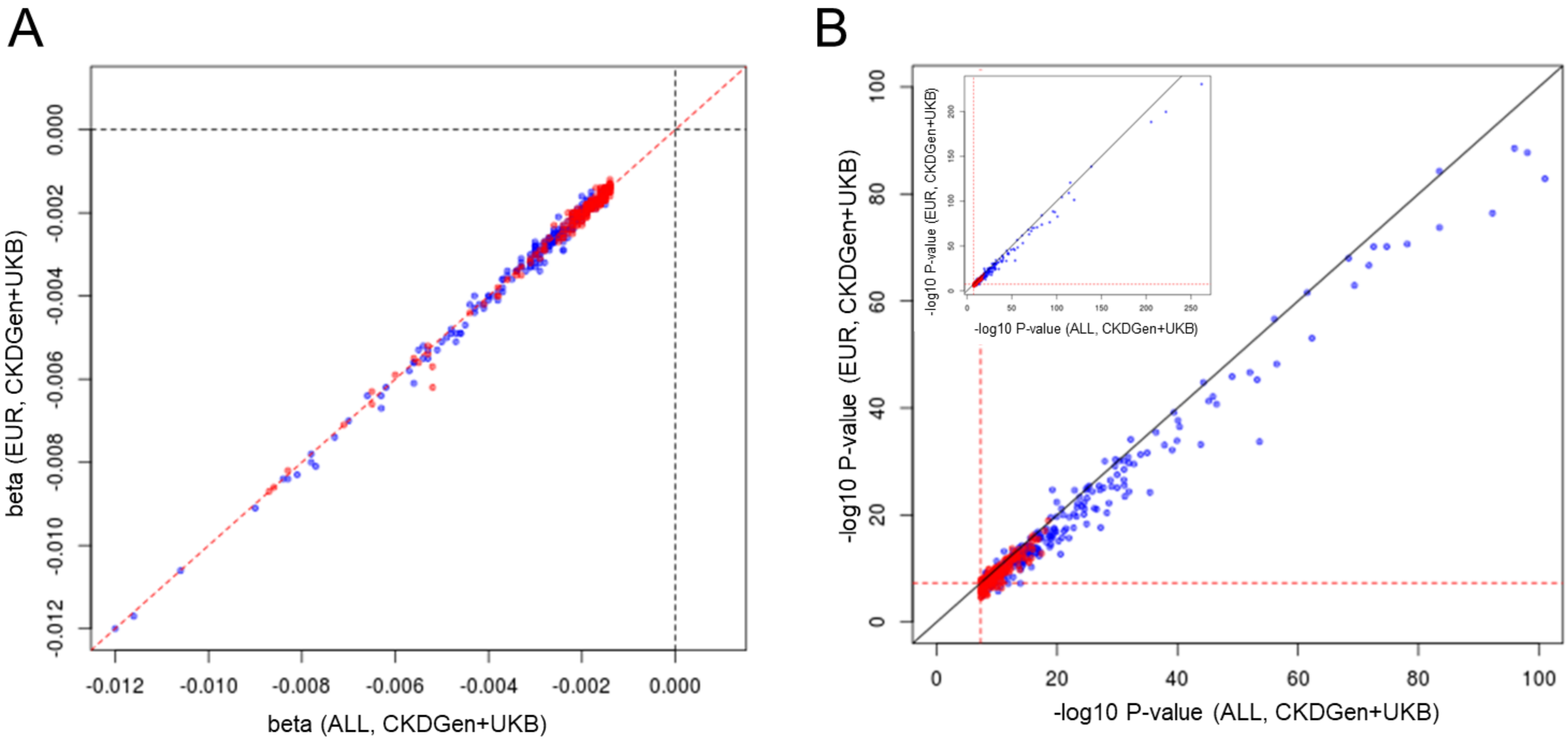

Supplementary Figure 4. Second stage power computation.

The figures show power curves to find a significant association in the independent second stage meta-analysis for varying sample sizes. Each curve represents one of the 424 lead variants identified by the primary meta-analysis (blue: 223 known variants; red: 201 novel variants). To derive power of a one-sided association test for each variant (y axis) depending on the second stage sample size (x axis), we set (i) the true underlying genetic effect size to the one observed in the primary meta-analysis (which may overestimate power due to potential winner's curse), (ii) the outcome variance to 0.28^2 (as observed for age-/sex-adjusted log eGFRcrea in the largest second stage dataset from MVP, hospital-based; 1st column panels: A,C,E) or to 0.13^2 (as observed for age-/sex-adjusted log eGFRcrea in ARIC, population-based, 2nd column panels: B,D,F) and (iii) the alpha-level for the "strict replication" (second stage alpha = 0.05/424, one-sided test, Bonferroni-corrected, 1st row panels: A,B), for the "alternative replication" (second stage alpha = 0.05, combined first and second stage alpha = $5x10^{-8}$, one-sided tests, 2nd row panels: C,D) or for a nominal significance threshold (second stage alpha = 0.05, one-sided, 3rd row panels: E,F).

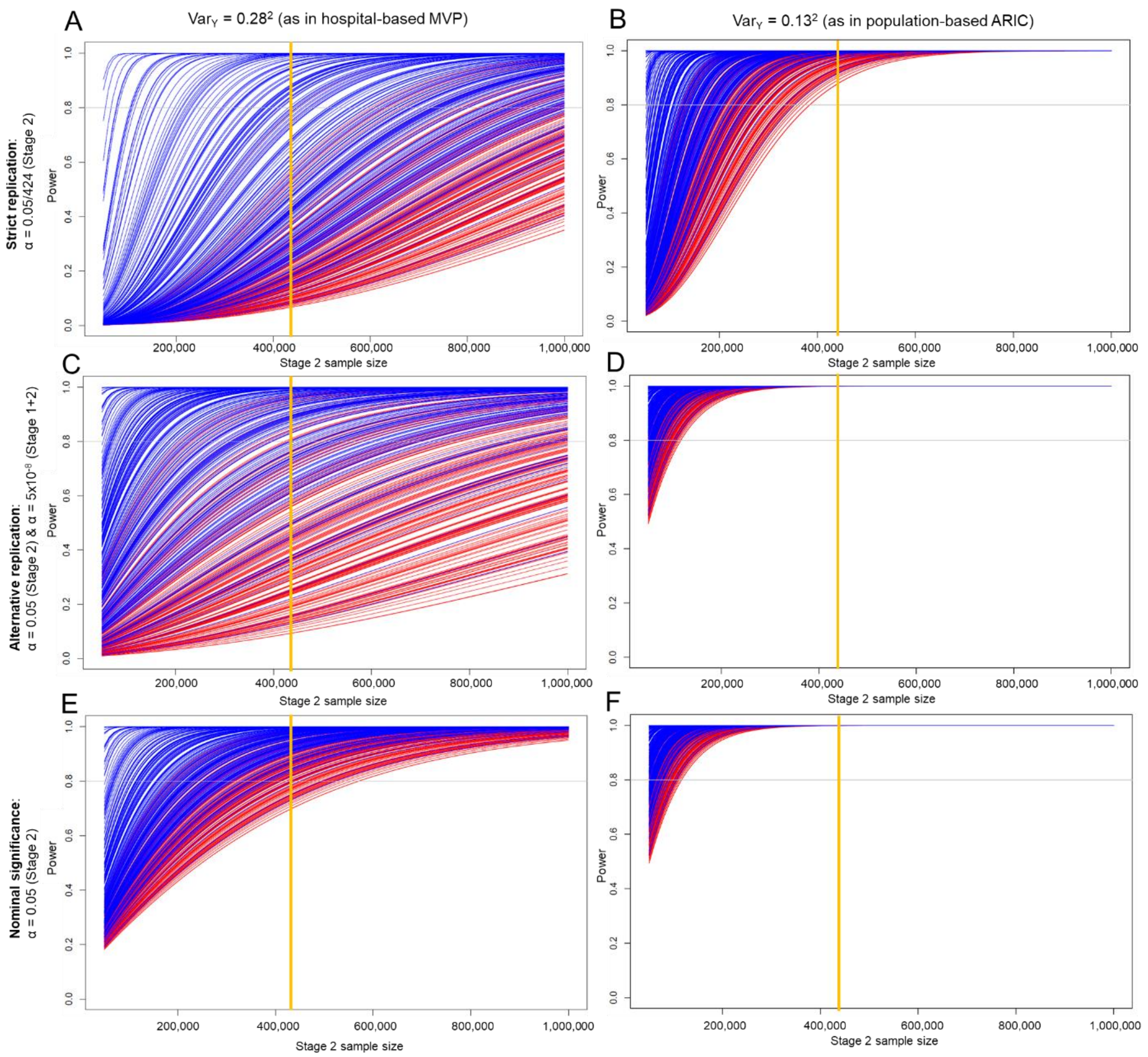

Supplementary Figure 5. Regional Association Plots (RAPs) for eGFRcrea association with variants at the 21 novel loci that included multiple independent signals.

The RAPs obtained with Locuszoom [2] show association results from the primary eGFRcrea meta-analysis (n = 1,201,929). Shown are 21 newly identified loci that included multiple independent signals in the stepwise approximate conditional analyses with GCTA [3]. P-values are reported without adjustment for the other signal(s) in the locus. Coloring denotes independent signals (using different colors based on LD structure to the signal index variants; as implemented in Locuszoom [2]) and correlation to the respective signal index variants (from dark = highly correlated to bright = uncorrelated).

Locus n1:

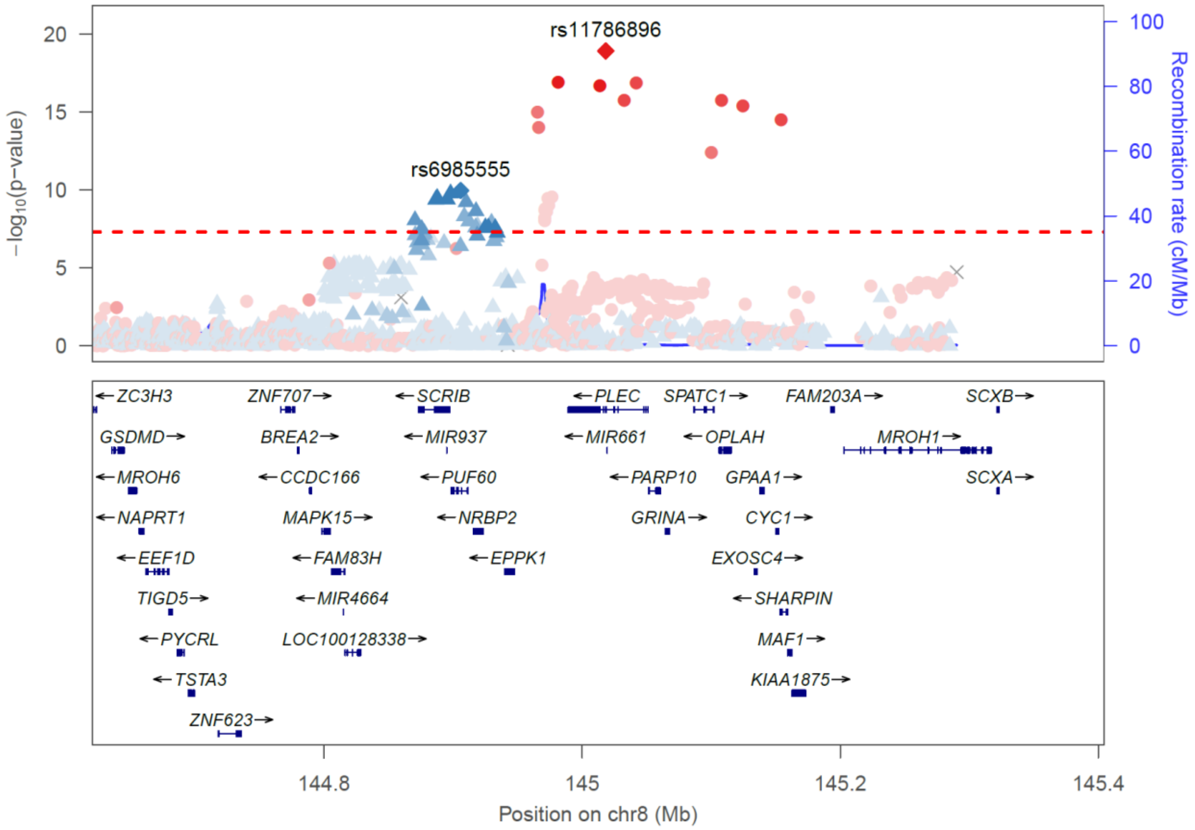

Locus n10:

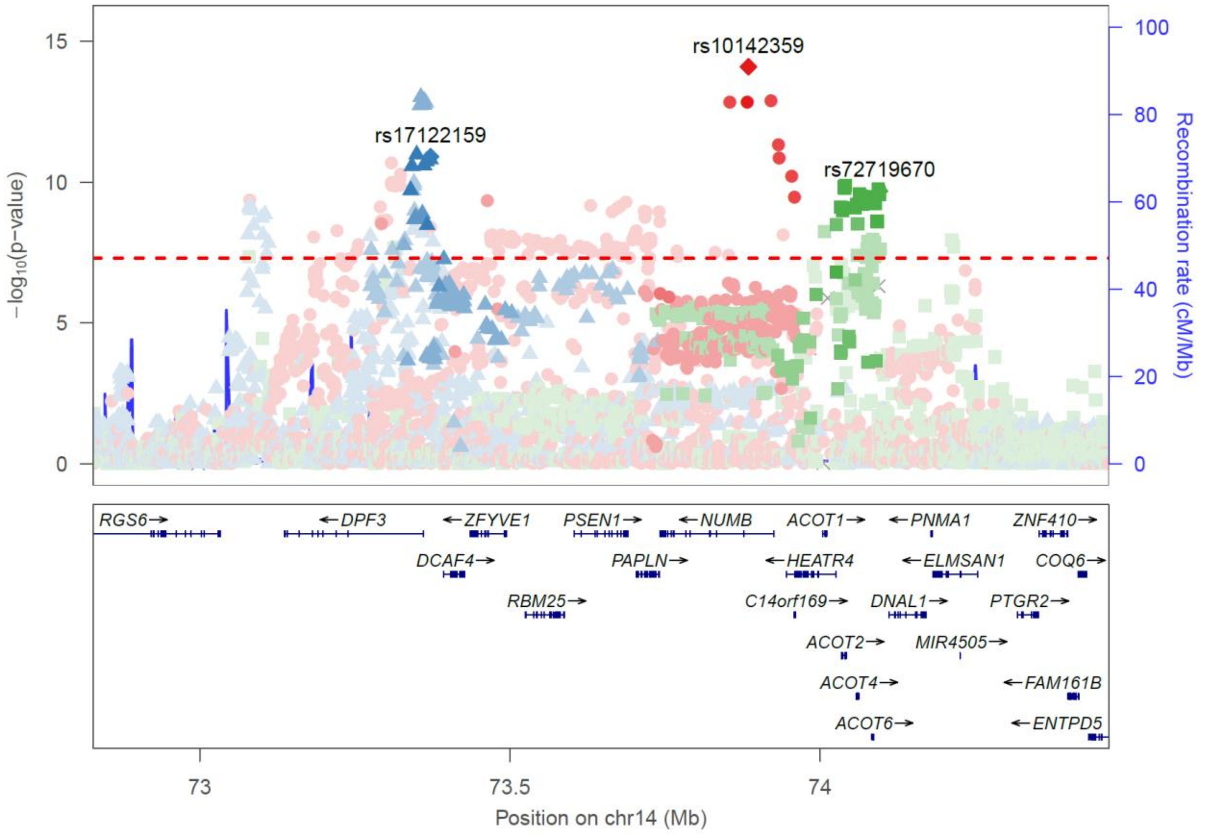

Locus n15

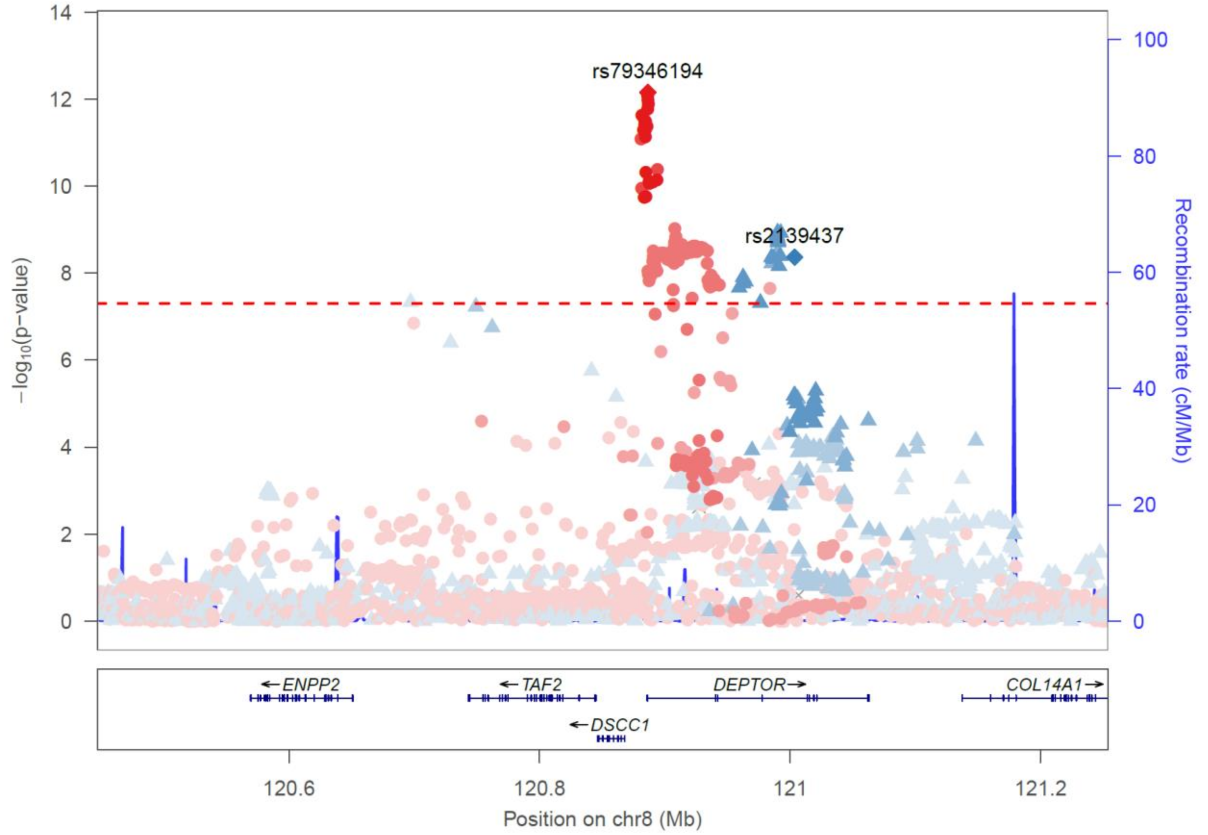

Locus n16

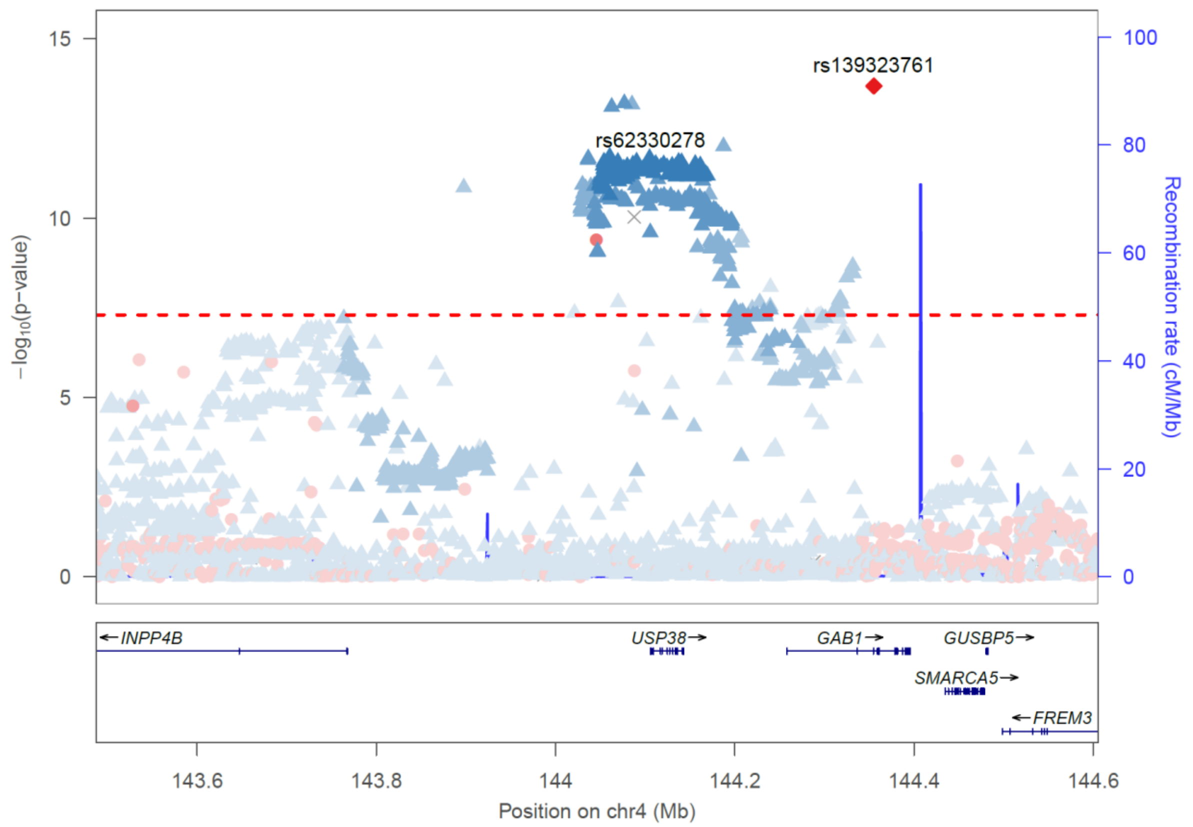

Locus n26

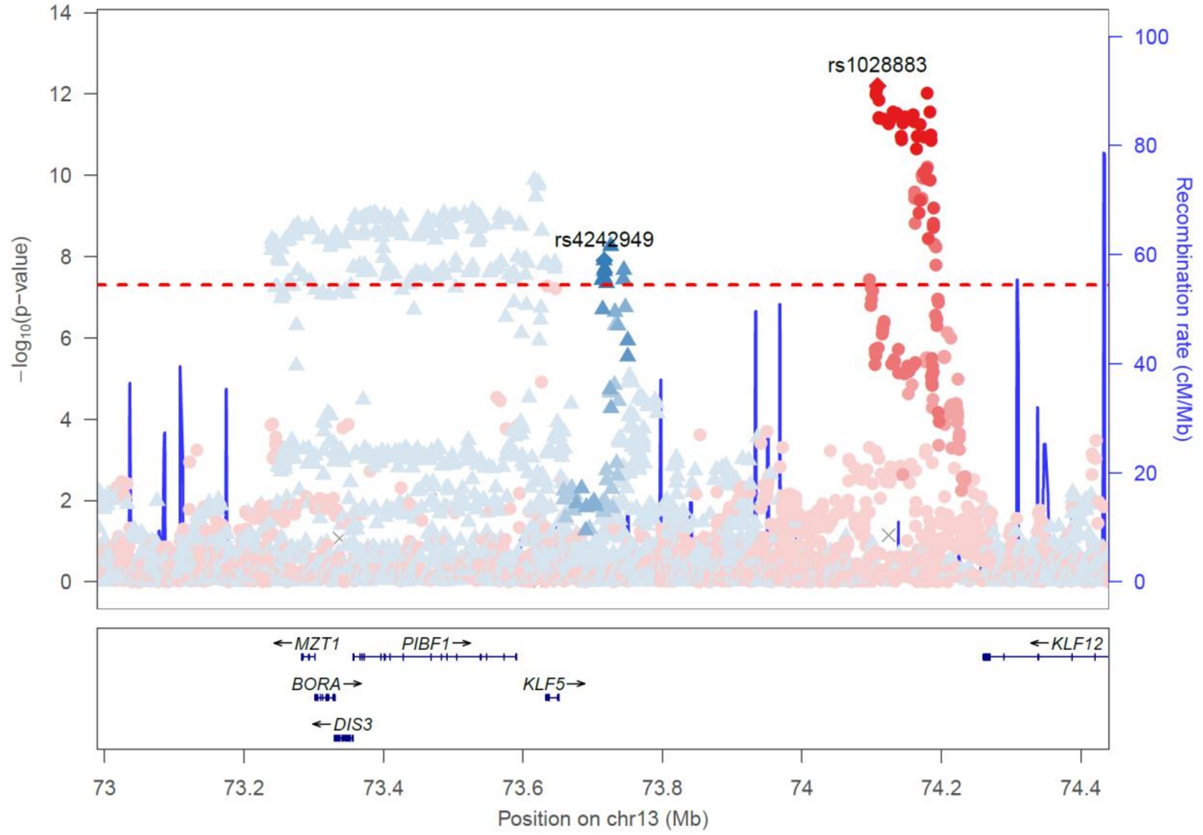

Locus n27

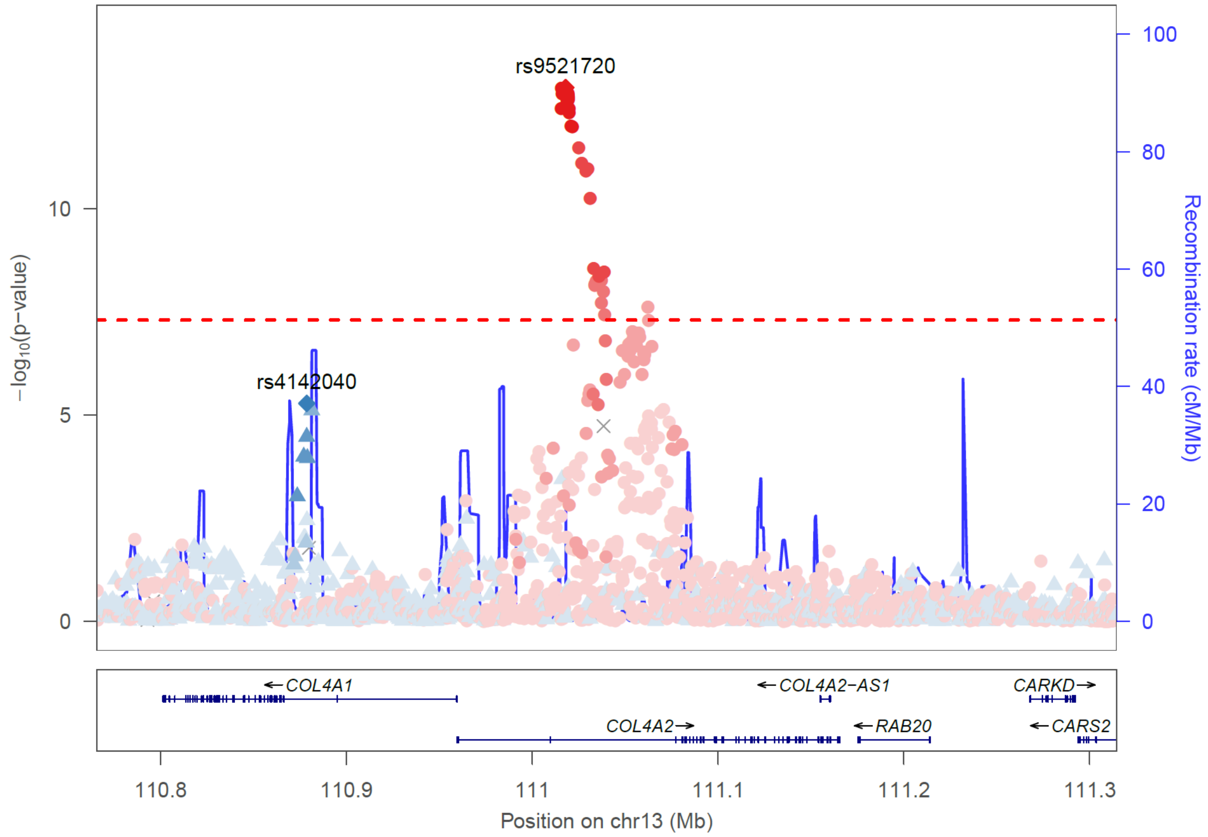

Locus n29

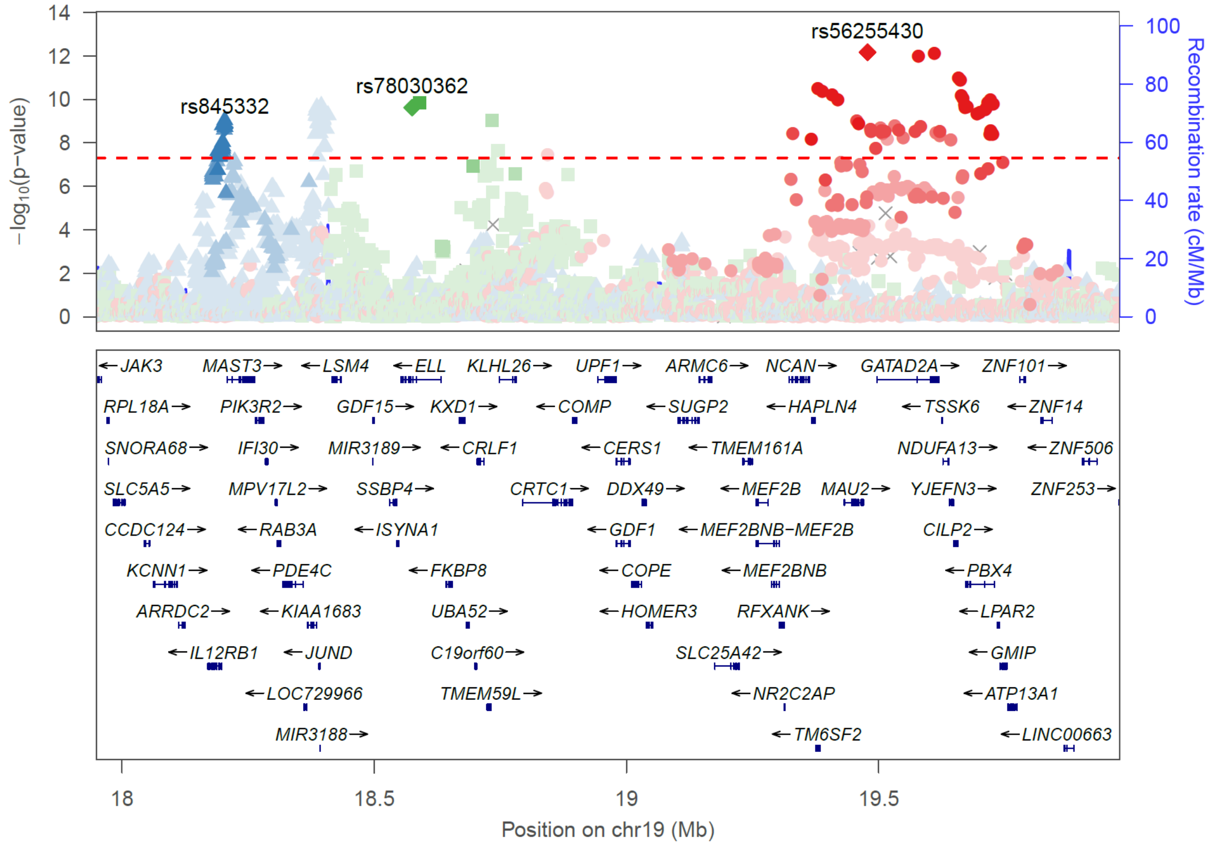

Locus n32

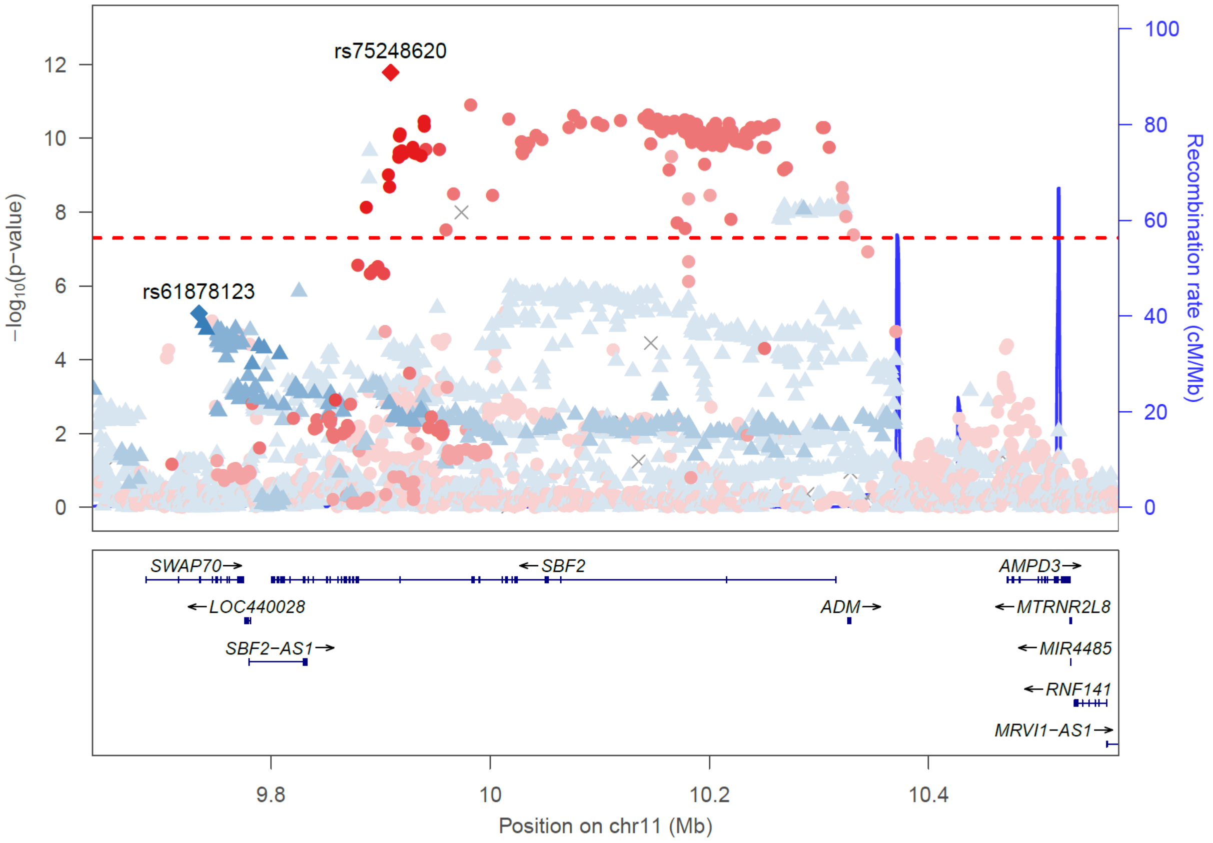

Locus n34

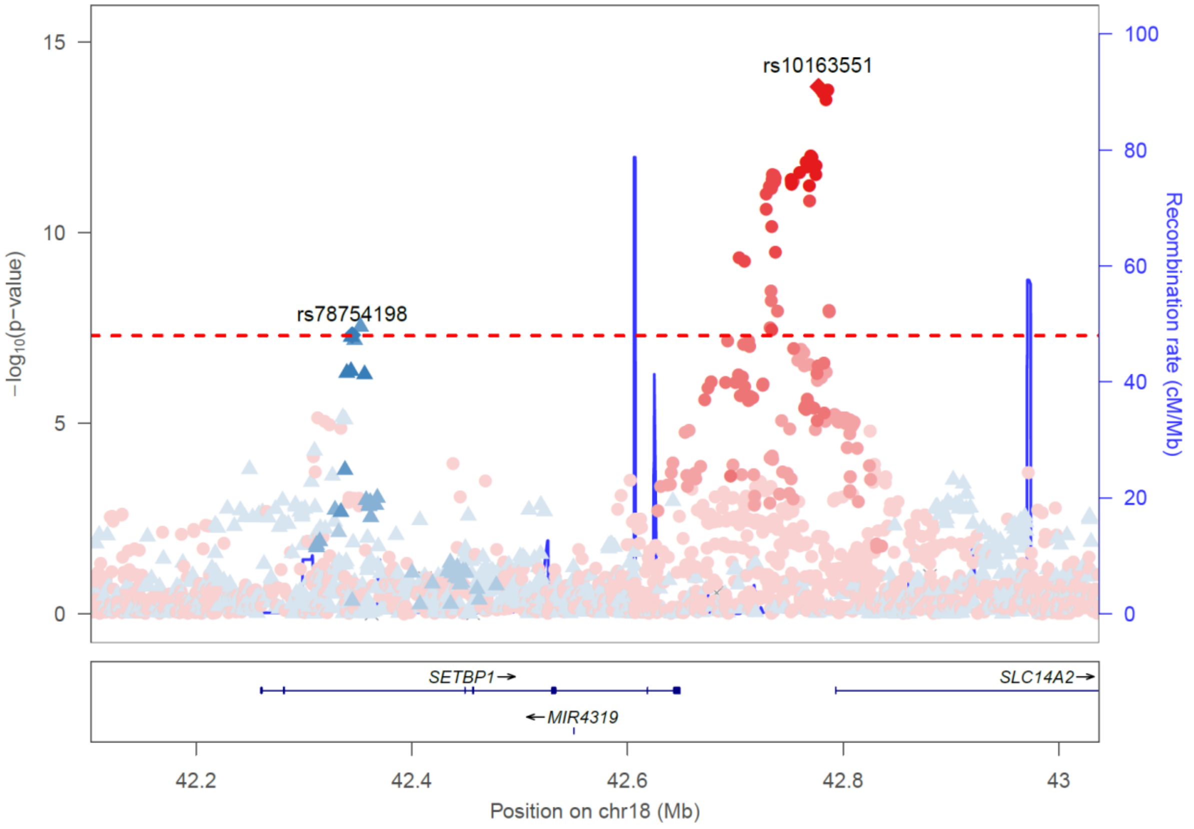

Locus n37

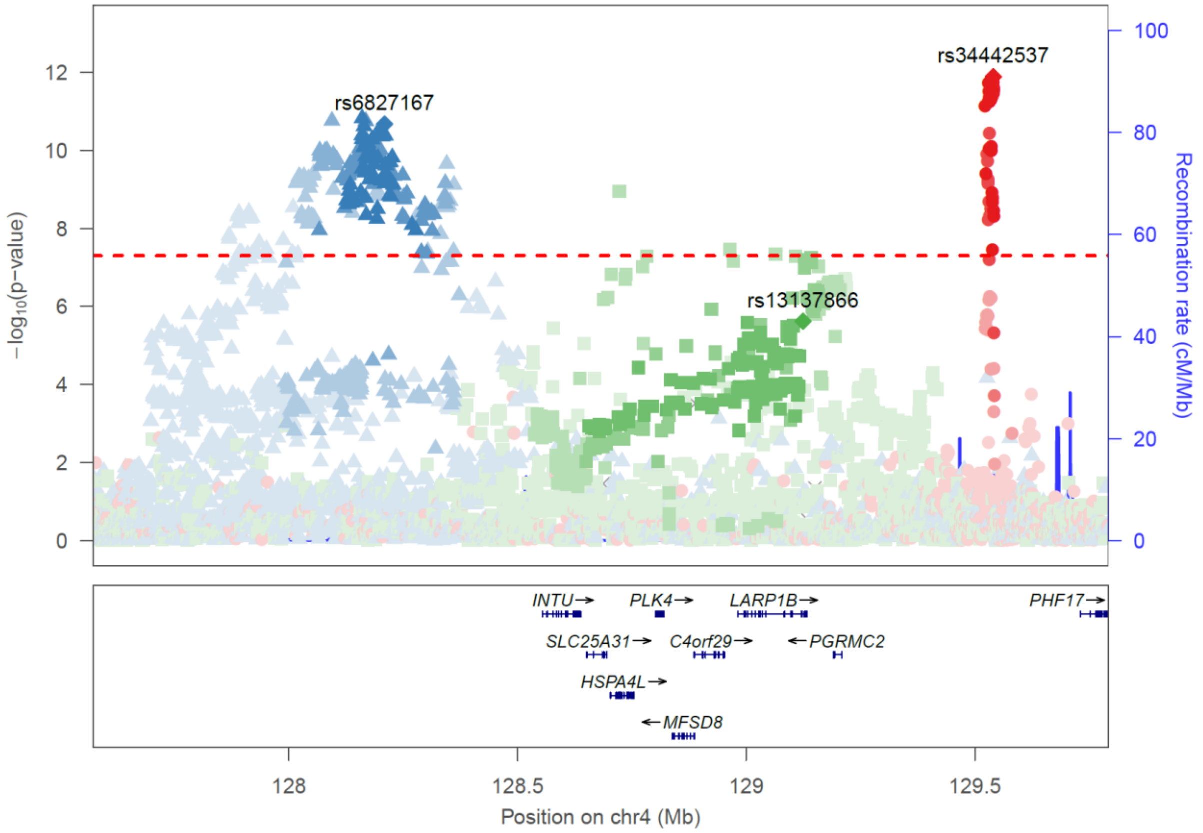

Locus n46

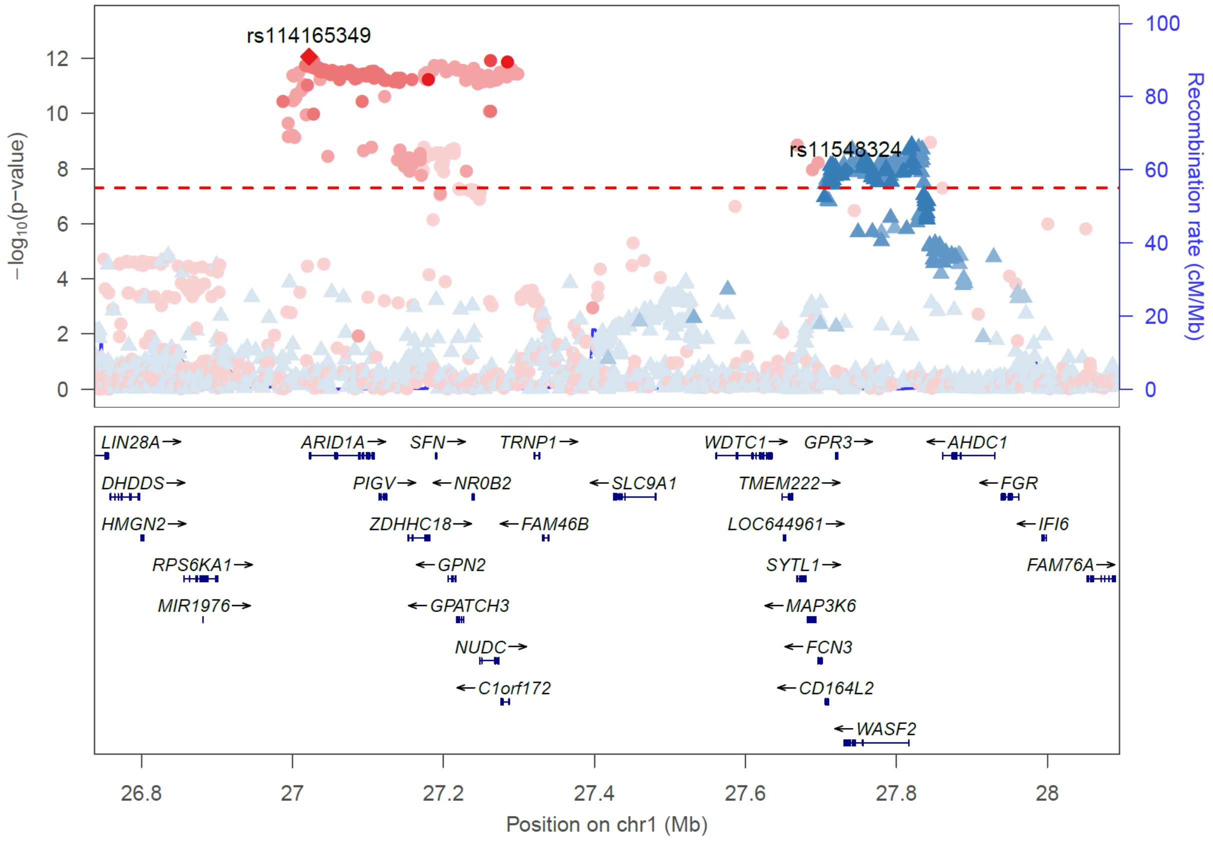

Locus n47

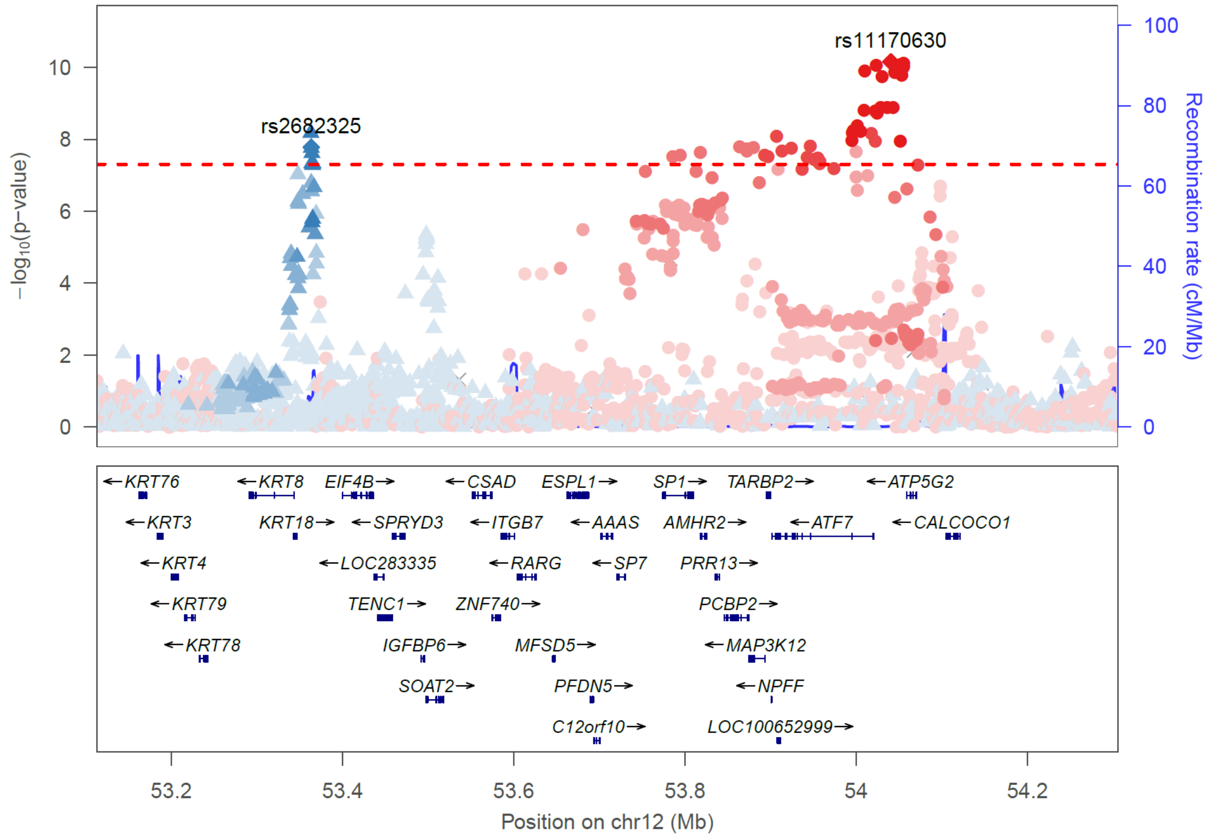

Locus n55

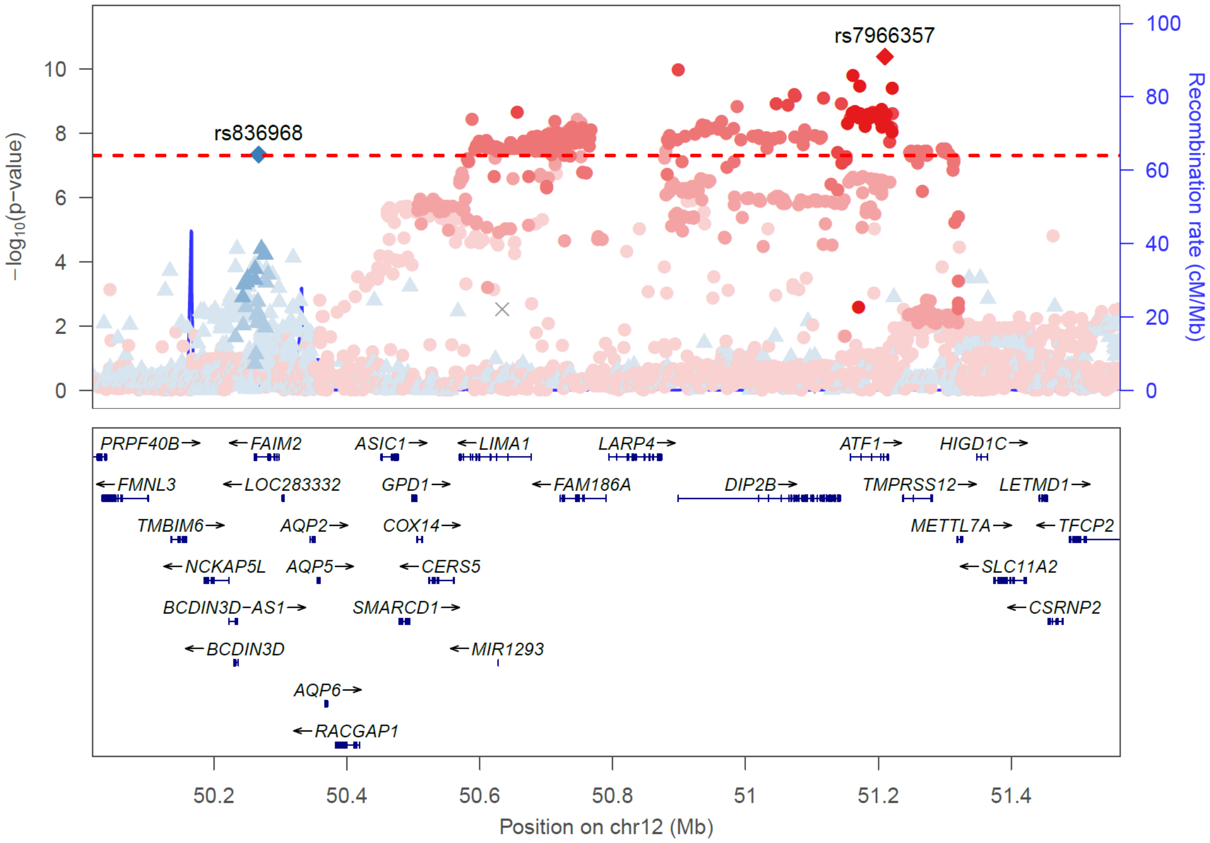

Locus n56

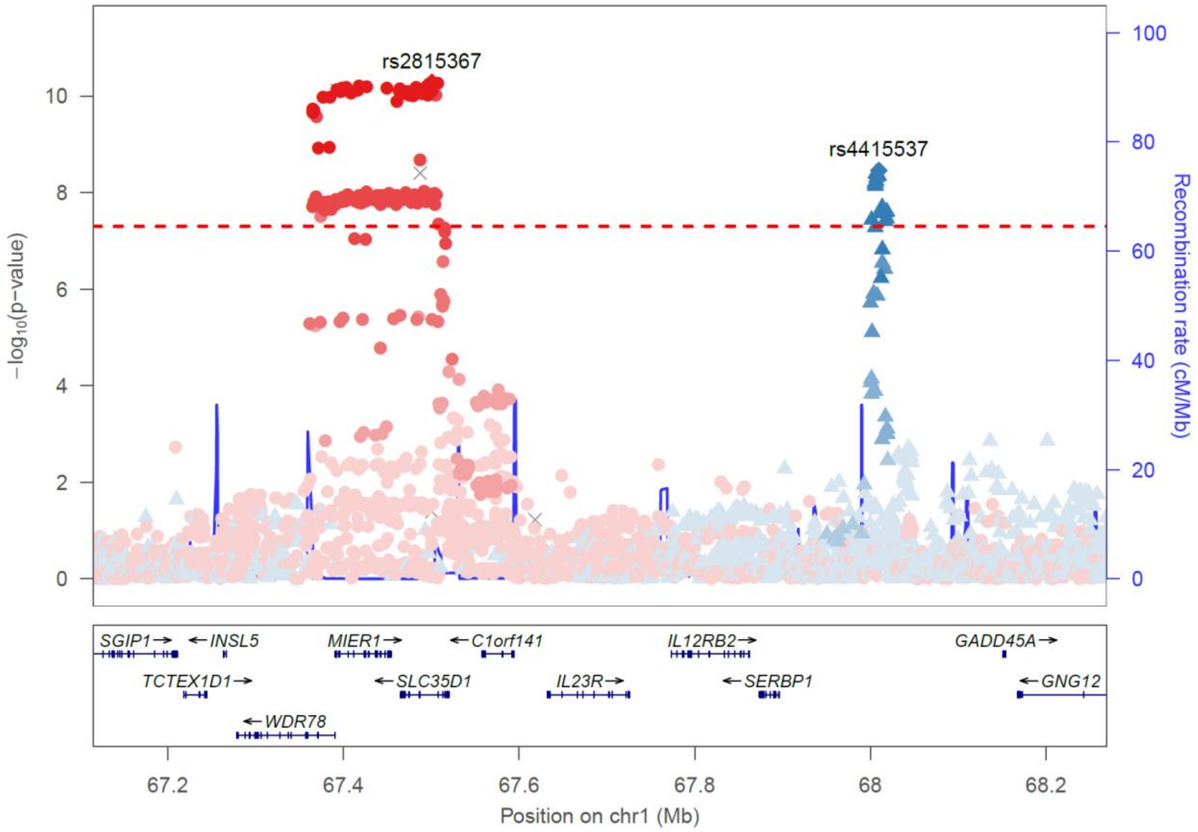

Locus n66

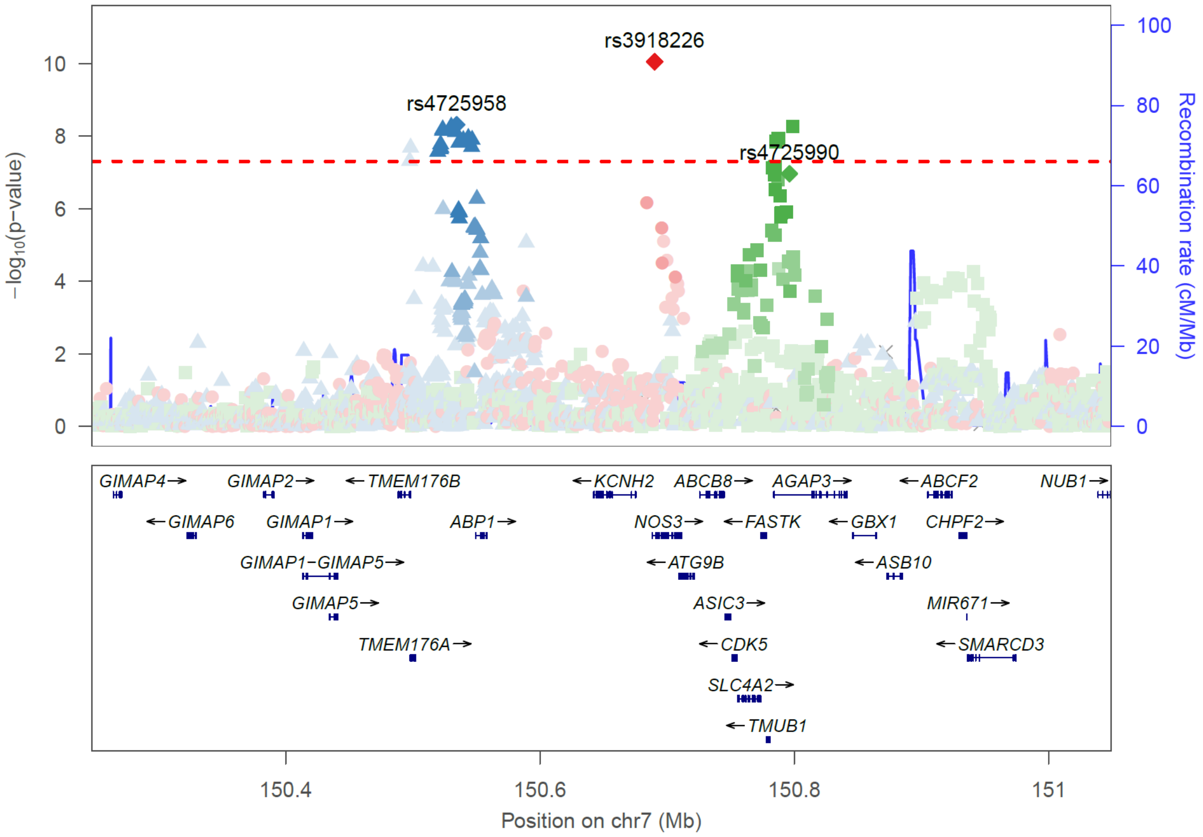

Locus n76

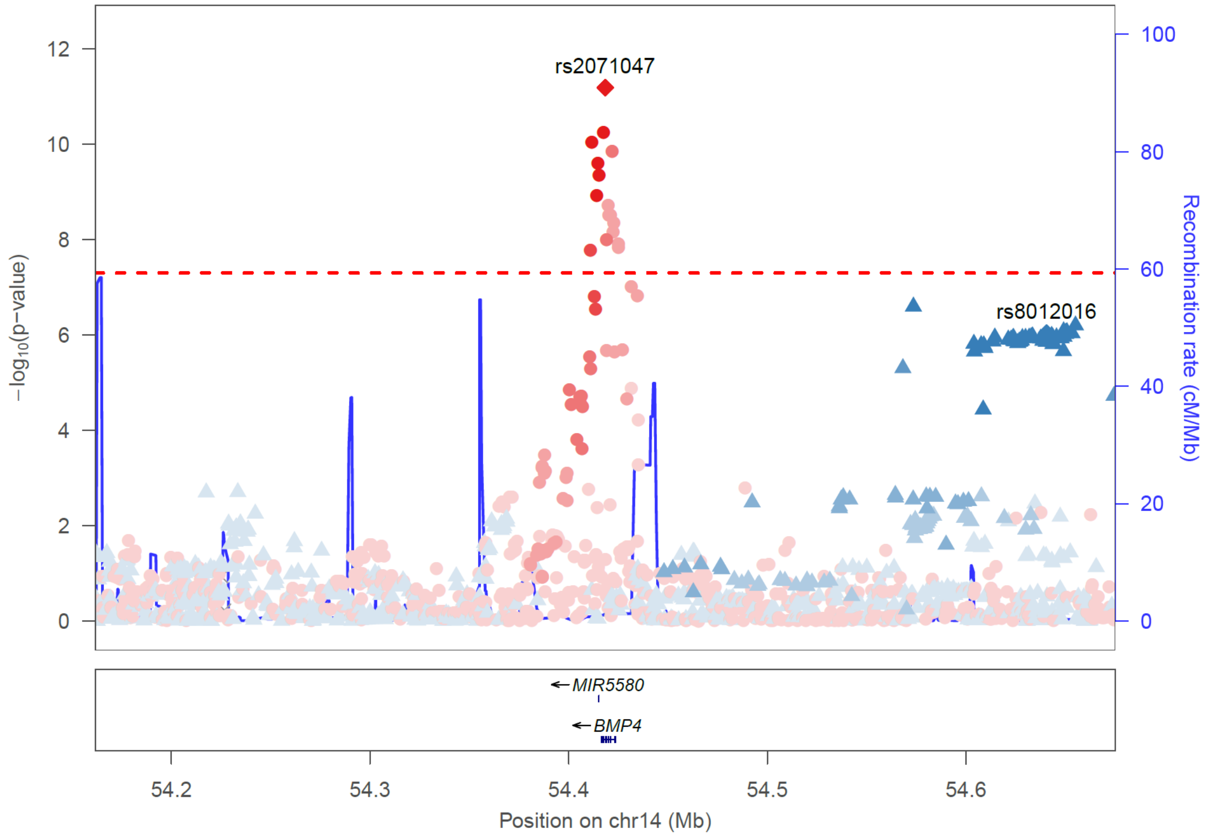

Locus n80

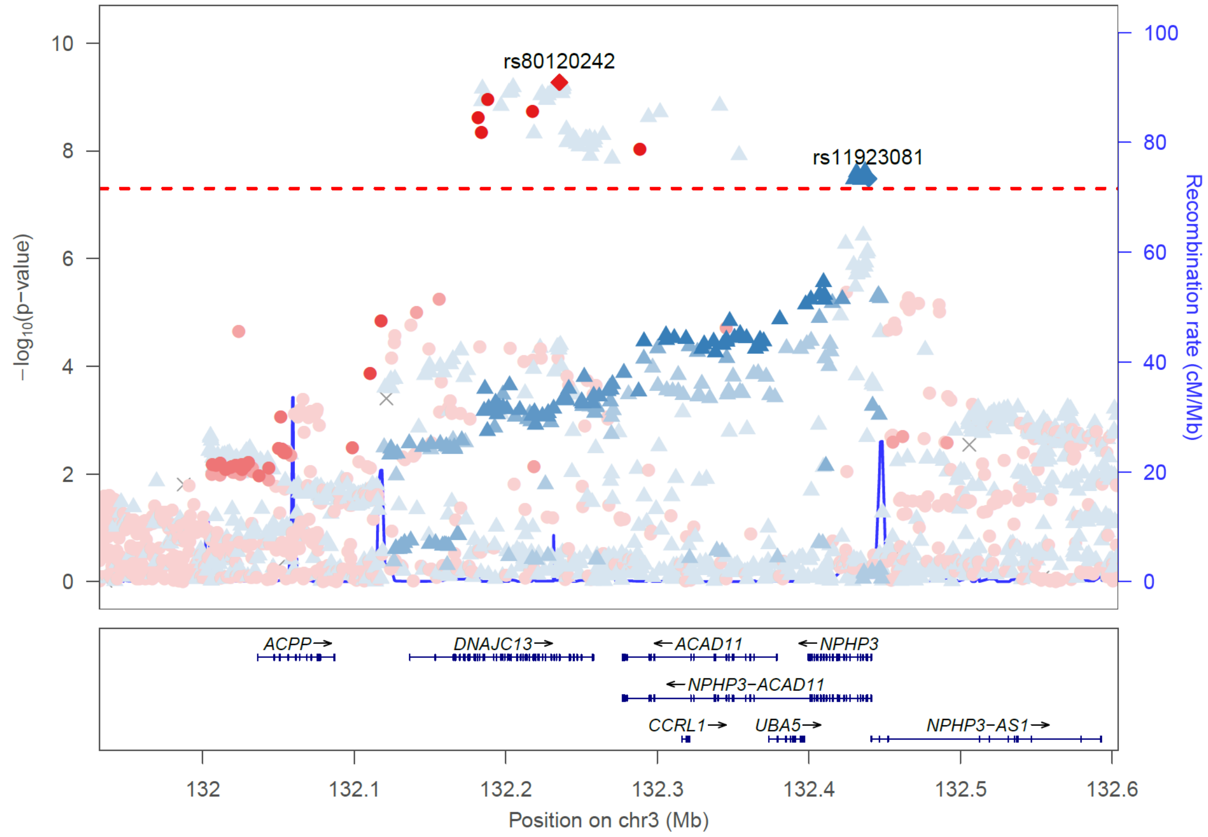

Locus n81

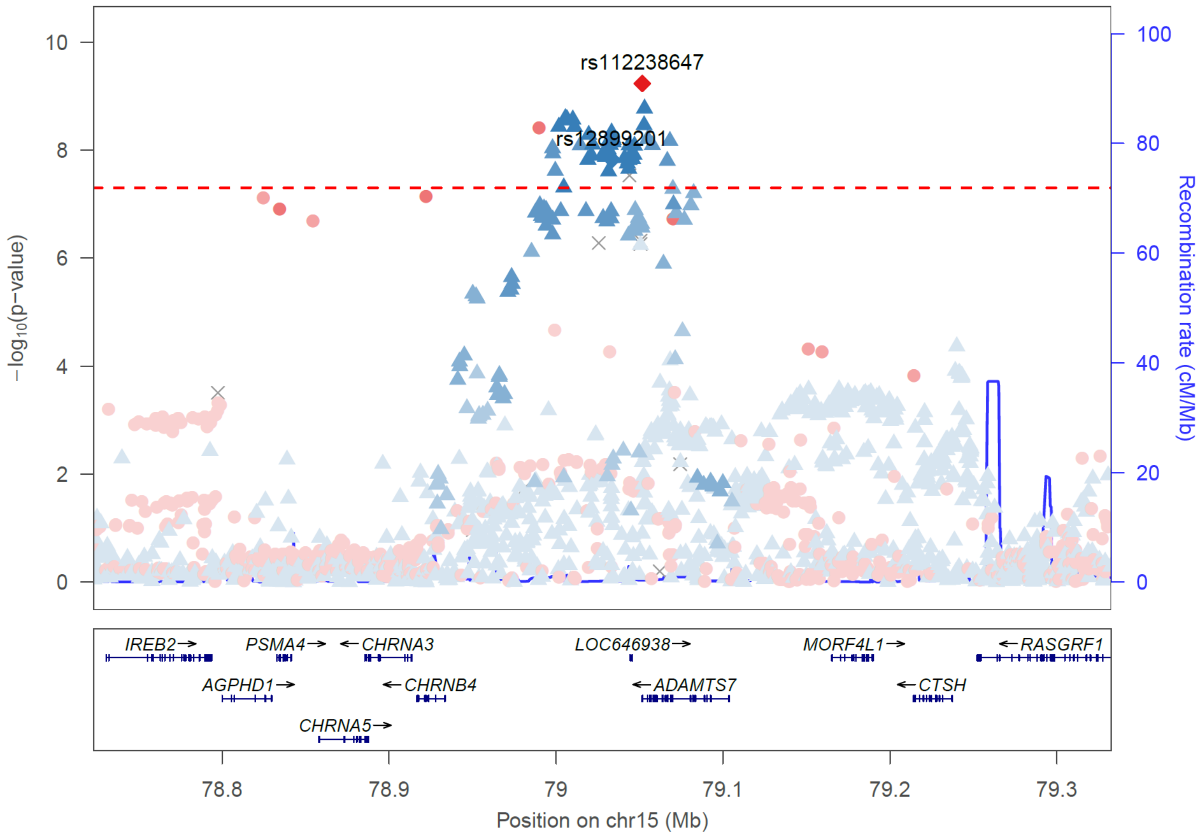

Locus n86

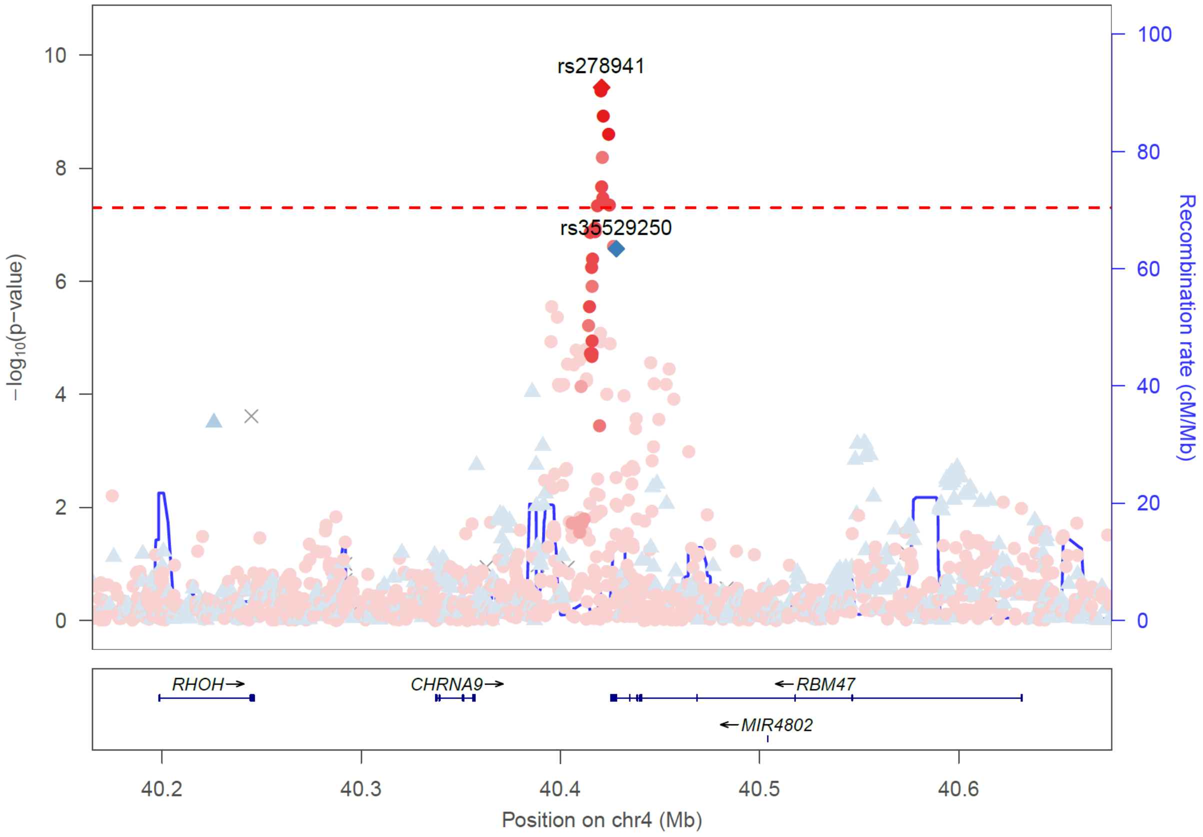

Locus n95

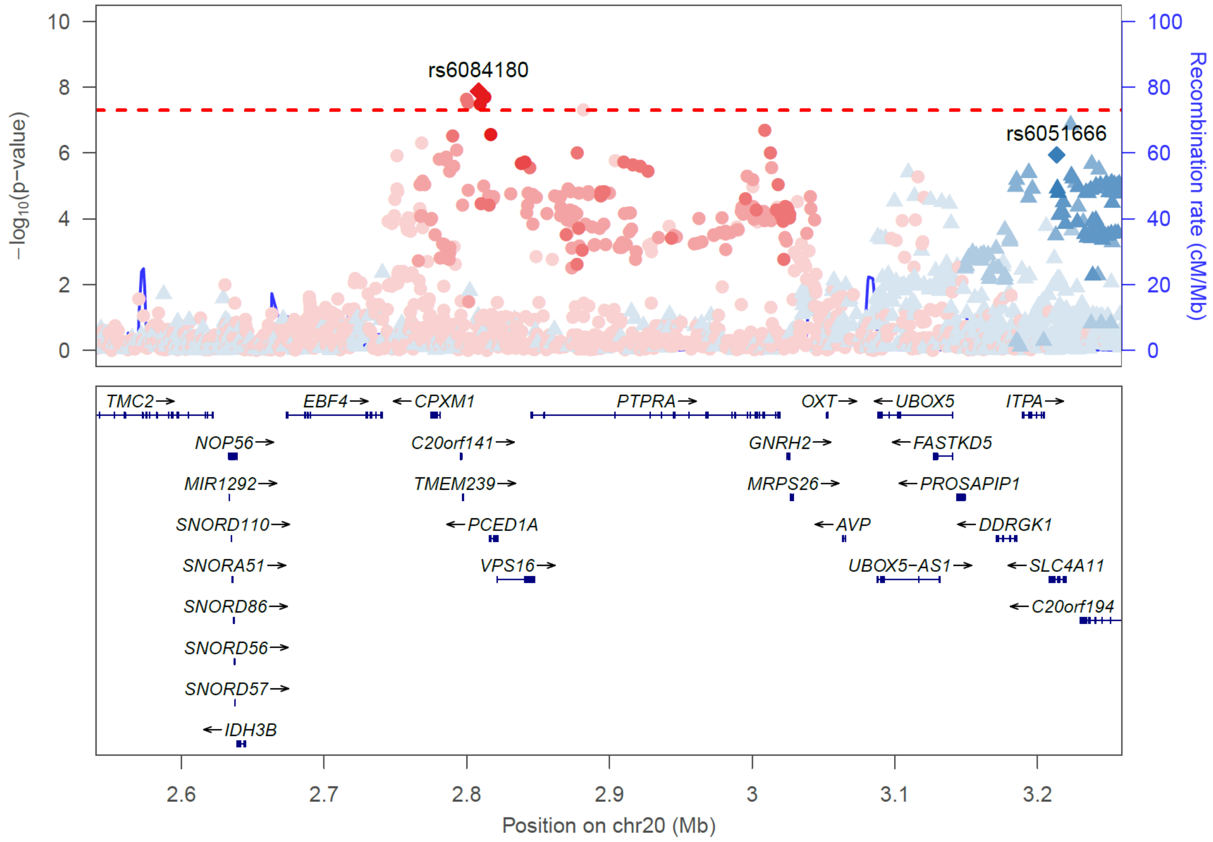

Locus n165

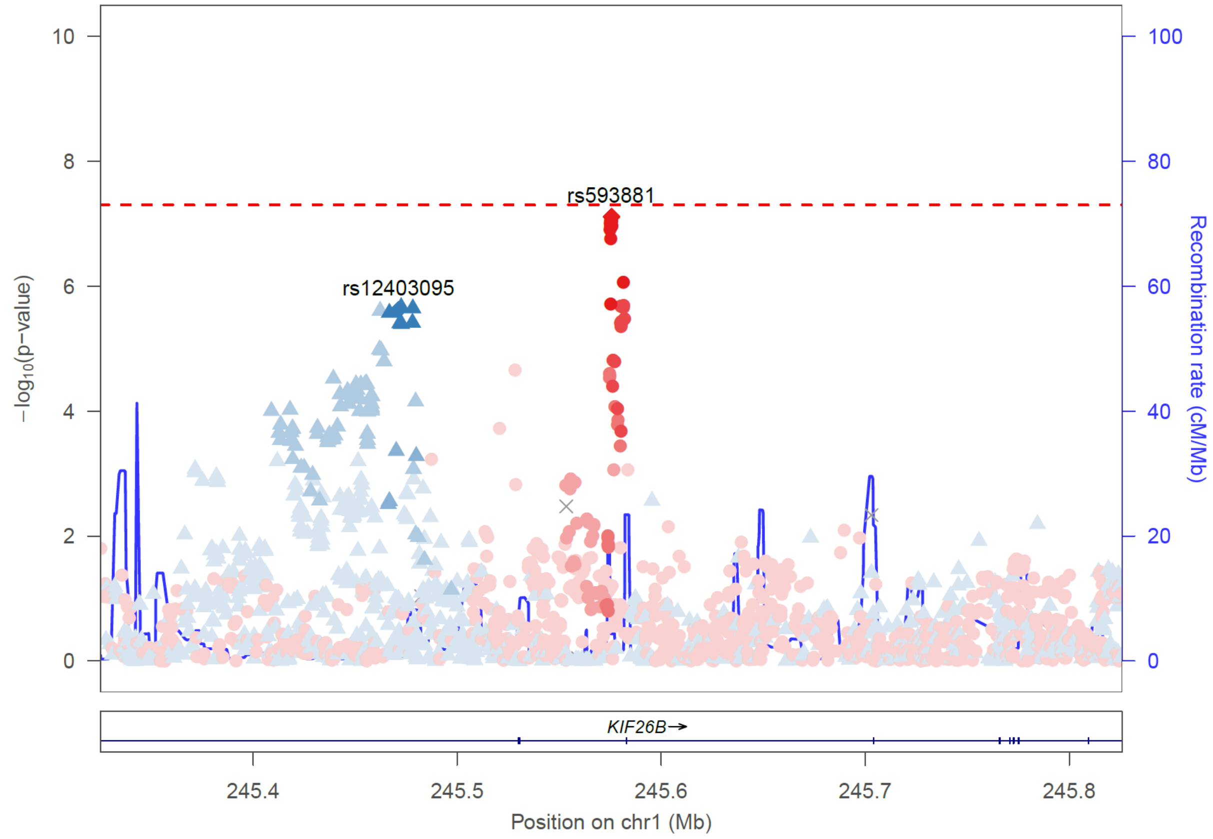

Supplementary Figure 6. Regional Association Plots (RAPs) at the *UMOD*/*PDILT* locus.

The RAPs show association results for eGFRcrea from the approximate conditional analyses with GCTA (n = 1,004,040, European-only meta-analysis). Shown are 4 independent signals observed in the *UMOD/PDILT* locus. For each signal, P-values are conditioned on the other three signal lead variants. Coloring denotes correlation to the respective signal lead variant.

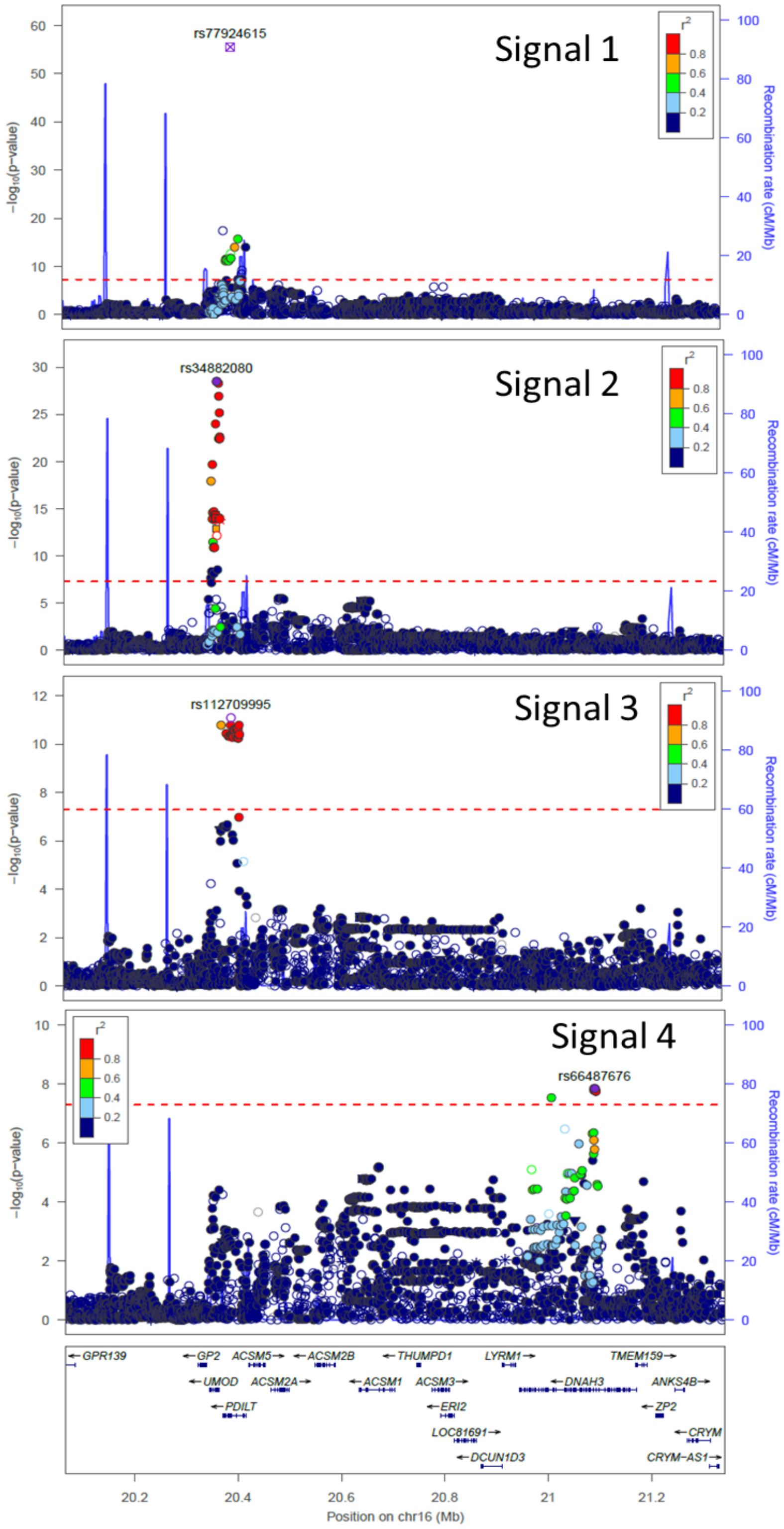

Supplementary Figure 7. Comparison of Posterior Probability of Association (PPA) between primary lead and fine-mapping variants.

The scatter plot compares the PPA of the primary analysis lead variants (all-ancestry meta-analysis, n = 1,201,929) with the maximum PPA observed among all credible set variants of the respective signals (based on EUR-only meta-analysis, n = 1,004,040). The 51 orange dots mark all-ancestry (primary) lead variants that are not contained in the respective 99% credible set. Among the black dots, 215 primary lead variants are precisely the variant with the highest PPA and 158 are contained in the respective 99% credible set.

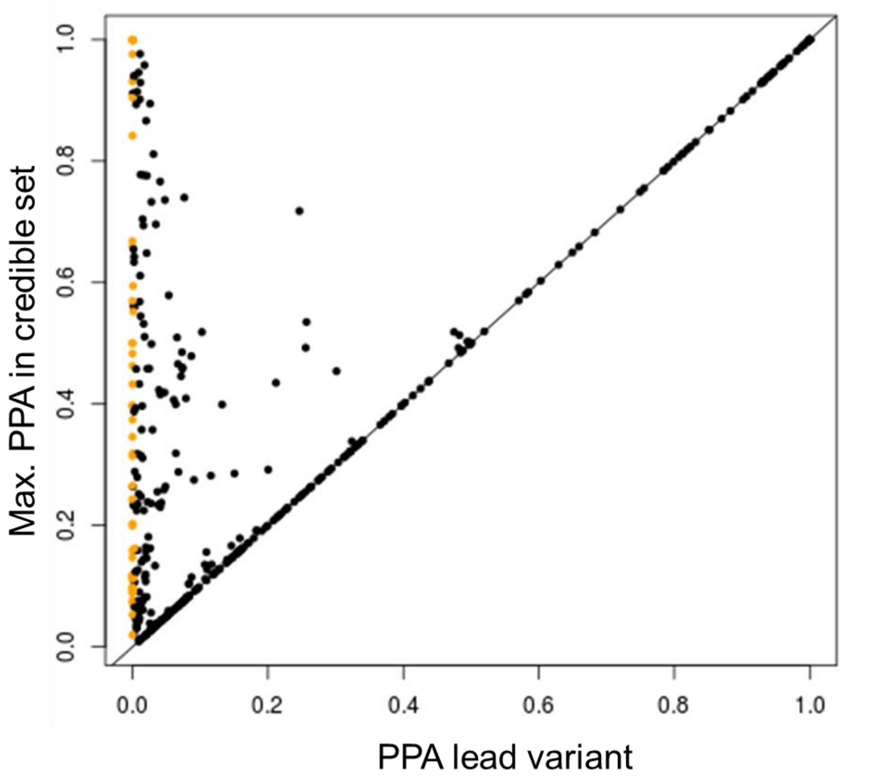

Supplementary Figure 8. Results from Gene PrioritiSation (GPS) based on variants that are available in the mostly-European CKDGen meta-analysis yields 31 genes.

Credible variants were limited to those being present in the CKDGen mostly-European meta-analysis result. By querying our GPS (**Supplementary Data 14**), we identified 31 genes that are mapping to eGFRcys/BUN validated loci and to a small credible set (≤ 5 variants) that contains a protein-relevant variant within the gene (CADD≥15) or a kidney-tissue regulatory variant (eQTL in NEPTUNE glomerulus or tubule-interstitial tissue; eQTL or sQTL in GTEx kidney tissue). Shown is the locus information (locus id, signal id, number of signals in the locus and the number of credible variants in the signal), variant information for credible variants within the gene (functional annotation, blue), for regulatory credible variants (regulatory annotation, orange) and gene information for kidney-related phenotypes (in mouse or human, green). Genes are grey if the PPA of the relevant variant is <10% or if the gene was previously highlighted by Wuttke et al without additional evidence [7]. Compared to our main result (**Figure 4**), *TPPP* is missing because its missense variant is not available in the mostly-European CKDGen meta-analysis result. The variant is however present in the European-only result that was used for our fine mapping analyses.

						99% credible variants within the gene with CADD ≥ 15			99% credible variants within the locus: eQTL				99% credible variants within the locus: sQTL		Evidenced kidney phenotype for gene				
						1	1	1	1	1	1	0	1	0	0	0			
Locus id	Signal id	num signals	Gene	credible variants in the signal	Score	stop-gained/ stop-lost/ non-synonymus	canonical-splice/ noncoding-change/ synonymous/ splice-site	other	NEPTUNE glomerulus	NEPTUNE tubulointerstitium	GTEx kidney	GTEx other	GTEx kidney	GTEx other	MGI Mouse	OMIM Human	Small PPA <10%	Highlighted in Wuttke et al.	Set comparison vs. Wuttke et al.
Single-sets with protein-relevant variants:																			
k24	1	1	*CERS2*	1	1	1	0	0	0	0	0	1	0	0	0	0	-	yes	newly single
k38	2	2	*SLC25A45*	1	1	1	0	0	0	0	0	0	0	0	0	0	-	yes	known single
k48	1	1	*CACNA1S*	1	1	1	0	0	0	0	0	0	0	0	0	1	-	yes	known single
k57	1	1	*EDEM3*	1	1	1	0	0	0	0	0	1	0	1	0	0	-	yes	known single
k128	3	4	*PKHD1*	1	1	1	0	0	0	0	0	0	0	0	22	3	-	-	newly single
k218	1	2	*RPL3L*	1	1	1	0	0	0	0	0	0	0	0	0	0	-	yes	known single
n30	1	1	*PDE7A*	1	1	1	0	0	0	0	0	0	0	0	0	0	-	-	new locus
n86	2	2	*RBM47*	1	1	1	0	0	0	0	0	0	0	0	0	0	-	-	new locus
k37	3	6	*HOXD11*	1	1	0	0	1	0	0	0	1	0	0	17	0	-	-	known single
k52	2	2	*SPEG*	1	1	0	0	1	0	0	0	0	0	1	0	0	-	-	newly single
n16	1	2	*GAB1*	1	1	0	0	1	0	0	0	0	0	0	0	0	-	-	new locus
Small-sets with protein-relevant variants:																			
k52	1	2	*SPEG*	2	1	1	0	0	0	0	0	2	0	2	0	0	-	-	newly small
k149	2	2	*ZC3HC1*	3	1	1	0	0	0	0	0	2	0	2	0	0	-	-	newly small
k179	1	1	*SOS2*	3	1	1	0	0	0	0	0	0	0	0	0	0	yes	-	known small
k191	2	2	*EFNA3*	4	1	1	0	0	0	0	0	0	0	3	0	0	yes	-	newly small
n14	1	1	*HNF1A*	4	1	1	0	0	0	0	0	0	0	0	3	1	-	-	new locus
n24	1	1	*NPHS1*	2	1	1	0	0	0	0	0	0	0	0	46	2	-	-	new locus
n69	1	1	*ANO9*	3	1	1	0	0	0	0	0	0	0	0	0	0	yes	-	new locus
n69	1	1	*SIGIRR*	3	1	1	0	0	0	0	0	0	0	0	0	0	-	-	new locus
n90	1	1	*AMPD1*	2	1	2	0	0	0	0	0	2	0	0	0	0	-	-	new locus
k7	2	4	*BCAS3*	3	1	0	0	1	0	0	0	0	0	0	0	0	-	-	newly small
k102	1	1	*CDC14A*	2	1	0	0	1	0	0	0	2	0	0	0	0	-	-	newly small
k134	2	2	*RRAGD*	2	1	0	0	1	0	0	0	2	0	0	0	0	-	-	newly small
n35	1	1	*IKZF2*	2	1	0	0	1	0	0	0	0	0	0	0	0	-	-	new locus
Small-sets with kidney regulatory variant:																			
k4	1	1	*GALNTL5*	2	1	0	0	0	0	2	0	0	0	0	0	0	-	yes	newly small
k21	2	4	*TFDP2*	4	1	0	0	0	0	4	0	4	0	0	0	0	-	-	newly small
k27	1	2	*SLC6A13*	2	1	0	0	0	0	2	0	2	0	2	0	0	-	-	newly small
k62	2	3	*CYP2D6*	4	2	0	0	0	0	0	2	4	2	4	0	0	yes	-	newly small
k62	2	3	*CYP2D7*	4	1	0	0	0	0	0	0	4	2	4	0	0	yes	-	newly small
k99	1	3	*PPDPF*	4	2	0	0	0	4	4	0	4	0	4	0	0	-	-	newly small
k191	2	2	*YY1AP1*	4	1	0	0	0	0	1	0	0	0	0	0	0	-	-	newly small
n95	1	2	*CPXM1*	4	1	0	0	0	4	0	0	3	0	0	0	0	-	-	new locus

Supplementary Figure 9. Locus based Gene PrioritiSation (GPS) of genes at novel loci causing Mendelian diseases with kidney involvement.

The GPS was filtered for genes at novel eGFRcys/BUN validated loci with at least one kidney related OMIM/Groopman et al. entry with any relevant variant (CADD >15 or eQTL/sQTL at any tissue). This yielded 29 genes including 6 genes with protein-altering variants (*BMP4, NPHS1, HNF1A, HOXA13, COL18A1, EVC*), 3 genes with another CADD>15 variant (*ACVRL1, ALG9, MAPRE2*) and one gene with eQTLs in GTEx kidney tissue (*NPHP3*). The other 19 genes showed regulatory variants in other GTEx tissues.

				credible variants within the gene			99% credible variants within the locus						Evidenced phenotype for gene	
				variants CADD>15			eQTL				sQTL			
Weight:				1	1	1	1	1	1	1	1	1	0	0
Locus id	Gene	credible variants per locus signal	Score	stop-gained/ stop-lost/ non-synonymus	canonical-splice/ noncoding-change/ synonymous/ splice-site	other	NEPTUNE glomerulus	NEPTUNE tubulointerstitium	GTEx kidney tissue	GTEx any tissue	GTEx sQTL kidney tissue	GTEx sQTL any other tissue	MGI Mouse kidney phenotype	OMIM Human kidney phenotype
n44	*ACTN4*	34	2	0	0	0	0	0	0	31	0	31	15	1
n62	*ACVRL1*	8	2	0	0	1	0	0	0	8	0	0	0	1
n132	*AHI1*	100	1	0	0	0	0	0	0	97	0	0	9	1
n51	*ALG9*	137	2	0	0	2	0	0	0	75	0	0	0	4
n39	*BCS1L*	13	2	0	0	0	0	0	0	12	0	12	1	2
n76	*BMP4*	10|52	4	1|0	0	1|0	0	0	0	10|0	0	1|0	32	2
n81	*CHRNA3*	7|69	1	0	0	0	0	0	0	4|68	0	0	3	1
n160	*COA8*	178	2	0	0	0	0	0	0	48	0	53	0	1
n138	*COL18A1*	58	3	1	0	0	0	0	0	48	0	47	5	2
n27	*COL4A1*	29|2	1	0	0	0	0	0	0	7|0	0	0	48	1
n55	*COX14*	261|20	2	0	0	0	0	0	0	237|0	0	199|0	0	2
n25	*DPH1*	37	2	0	0	0	0	0	0	33	0	1	0	2
n148	*ENG*	72	2	0	0	0	0	0	0	3	0	12	0	1
n141	*EVC*	38	3	2	0	0	0	0	0	9	0	32	0	1
n141	*EVC2*	38	1	0	0	0	0	0	0	4	0	0	0	1
n14	*HNF1A*	4	1	1	0	0	0	0	0	0	0	0	3	1
n102	*HOXA13*	9	1	1	0	0	0	0	0	0	0	0	3	2
n37	*INTU*	47|106|231	2	0	0	0	0	0	0	0|0|210	0	0|0|18	1	1
n36	*LAMC2*	56	2	0	0	0	0	0	0	53	0	2	2	1
n186	*MAPRE2*	22	3	0	0	1	0	0	0	22	0	14	0	1
n125	*NDUFAF6*	49	2	0	0	0	0	0	0	23	0	23	0	1
n80	*NPHP3*	12|47	3	0	0	0	0	0	0|12	12|45	0	0|37	14	6
n24	*NPHS1*	2	1	1	0	0	0	0	0	0	0	0	46	2
n182	*PIK3C2A*	72	1	0	0	0	0	0	0	1	0	0	11	1
n79	*PTPN11*	622	1	0	0	0	0	0	0	192	0	0	0	2
n44	*SARS2*	34	1	0	0	0	0	0	0	1	0	0	0	1
n111	*SLC26A1*	91	1	0	0	0	0	0	0	11	0	0	2	1
n194	*STK11*	9	1	0	0	0	0	0	0	2	0	0	1	1
n17	*SUCLA2*	26	2	0	0	0	0	0	0	20	0	11	0	1

Supplementary Figure 10. Comparison of colocalization and FDR-based expression analyses.

The scatter plots compare results from two approaches to evaluate the identified 634 eGFRcrea signals for gene expression effects in two kidney tissues from NEPTUNE [4] (A: tubule-interstitium, B: glomerulus). The first approach is two conduct locus-based colocalization analyses of the effects of variants on eGFRcrea (European-only, n = 1,004,040) and gene expression (two kidney tissues from NEPTUNE). Posterior probability of positive colocalization (PP_{H4}) is shown on the x-axis with $PP_{H4}\geq80\%$ denoting 'positive' colocalization between the eGFRcrea signal and the gene expression effects. The other approach is to evaluate the signal's 99% credible variants for significant expression effects. The minimum false-discover-rate (FDR) for gene expression effects observed among the 99% credible variants of the respective signal/gene combination is shown on the y axis, with FDR<5% denoting significant gene expression effects for the variant. Coloring denotes the maximum posterior probability of association (PPA) observed among the respective signals's 99% credible set variants.

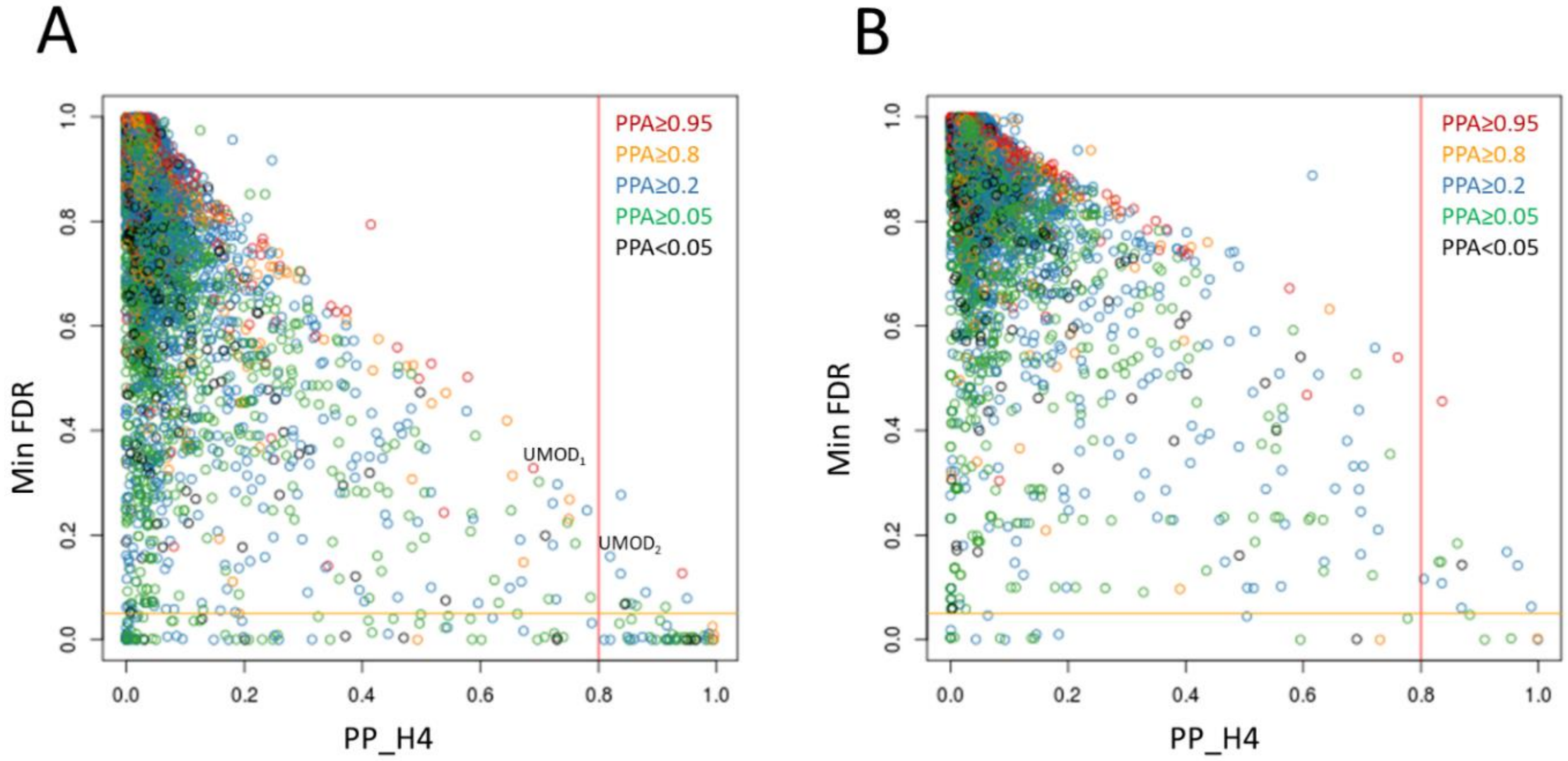

SUPPLEMENTARY NOTE

Supplementary Note 1. Power computation for second stage meta-analysis.

To verify our identified variants, we evaluated their association with log(eGFRcrea) in an independent second meta-analysis comprising up to 417,288 individuals from the Million Veterans Program (MVP, n = 300,680, hospital-based), the Michigan Genomics Initiative (MGI, n = 47,219, , hospital-based) and HUNT (n = 69,389, population-based, **Supplementary Data 1**). Our power computations show that, despite the large sample size, power in this second meta-analysis is limited to replicate the novel loci (**Supplementary Figure 4**): For a 1-sided alpha-level of 0.05/424 = 1.2×10^{-4}, with the second meta-analysis sample size (n = 417,288) and an outcome variance = 0.28^2 (variance of age-/sex-adjusted log eGFRcrea in MVP and MGI), we had >80% power to detect the 70 of the 223 known loci but for none of the 201 novel loci (assuming the stage 1 observed effect sizes and allele frequencies were the truth, **Supplementary Figure 4A**). This strongly depends on the outcome variance: for the outcome variance assumed at 0.13^2 (variance of age-/sex-adjusted log eGFRcrea in ARIC[1]), we would have had >80% power for all 424 variants (**Supplementary Figure 4B**). Even with a less-conservative alpha-level of 1-sided 0.05 in the second meta-analysis combined with a 1-sided genome-wide alpha=5×10^{-8} in the combined primary + secondary meta-analysis, we would have had > 80% power only for 153 of the 223 known and 24 of the 201 novel loci for an outcome variance of 0.28^2 (compared to 424 of the 424 loci for outcome variance =0.13^2, **Supplementary Figure 4C+D**). Even with a semi-formal criterion for replication, when judging variants in the second meta-analysis at one-sided alpha=0.05, only 135 of the 201 novel loci would have been detectable with 80% power for an outcome variance of 0.28^2 (again compared to 424 of the 424 loci for outcome variance =0.13^2, **Supplementary Figure 4E+F**). The limited power hampered the full account of this second data as a strict replication stage, where identified variants are restricted to those replicated for further follow-up. We considered it as additional independent evidence, which can be taken-into-account, when limiting the false positive rate is a primary concern.

Supplementary Note 2. 21 genes mapping to small credible sets of size 2-5 variants.

Among the 32 genes mapping to 99% credible sets with 5 or less variants, 11 mapped to a single-variant 99% credible set (predicted function of genes with novel evidence described in the main text) and 21 additional mapped to a set of size 2-5. The *SPEG* gene mapped to a single credible variant set and to an independent 2-5 credible variant set. Among the 2-5 variant genes: (i) 8 genes plus *SPEG* mapped to protein-altering variants including 4 at known loci [1] (known-small credible set: *SOS2*; newly small credible set: *EFNA3, SPEG,* and *ZC3HC1*) and 5 at novel loci (*AMPD1, ANO9, HNF1A, NPHS1* and *SIGIRR*). While *ANO9*, *SOS2* and *EFNA3* were less convincing with the variant PPA < 10%, all others had a PPA of 39.7% to 98.0% implicating a high probability of the variant and the thus the gene being causal. (ii) 4 genes mapped to 'other' CADD≥15 variants including 3 at known loci (newly small: *BCAS3*, *CDC14A* and *RRAGD*) and 1 at a novel locus (*IKZF2*). All had a PPA of 36.9% to 93.2% suggesting the variants and genes as causal. (iii) 9 genes mapped to eQTL/sQTL variants in kidney tissue including 8 at known loci (newly small: *CYP2D6*, *CYP2D7, GALNTL5*, *PPDPF, SLC6A13*, *TFDP2*, *TPPP* and *YY1AP1*) and 1 at a novel locus (*CPXM1*). While the variants mapping to *CYP2D6-D7* were less convincing with PPA<10%, the other eQTL/sQTL variants were the ones with the best statistical evidence for driving the association signal (PPA 49.98% to 93.2%).

Supplementary Note 3. Detailed description of the 23 highlighted genes with novel evidence.

The following list shows a detailed description for the 23 highlighted genes with novel evidence (**Table 2**):

AMPD1: Encodes for the AMP deaminase 1 that catalyzes deamination of AMP to IMP in skeletal muscle [58], autosomal recessive mutations (including rs17602729) cause Myopathy due to myoadenylate deaminase deficiency (MIM: 615511).

BCAS3: Encodes for Breast carcinoma-amplified sequence 3, plays a role in angiogenesis and tumor metastasis [46].

CDC14A: Encodes for the dual specificity protein phosphatase CDC14A, plays major role in centrosome separation and chromosome segregation [47], rare homozygous mutations cause Deafness, autosomal recessive 32, with or without immotile sperm (MIM: 608653).

CERS2: Encodes for the Ceramide synthase 2, uses very long acyl-chain-CoAs and ceramide to form sphinganine [38], reduced ceramide levels in kidney and renal parenchyma abnormalities in k.-o. mice [39].

CPXM1: Encodes for Probable carboxypeptidase X1, a secreted collagen-binding glycoprotein without carboxypeptidase activity [59].

GAB1: Encodes for GRB2-associated-binding protein 1, involved in regulating cell polarity[43].

GALNTL5: Encodes for N-acetylgalactosaminyltransferase-like protein 5, necessary for mammalian sperm formation [48].

HNF1A: Encodes for the transcription factor Hepatocyte nuclear factor 1-alpha, regulates expression of multiple genes [60], mutations cause diabetes mellitus insulin-dependent (MIM:222100, MIM: 612520) and noninsulin-dependent (MIM:125853), MODY type 3 (MIM: 600496), somatic hepatic adenoma (MIM:142330) and renal cell carcinoma (MIM:144700), rs1800574 is associated with higher risk of diabetes type 2 [34].

HOXD11: Encodes for the transcription factor Homeobox protein Hox-D11, important for normal ureteric bud branching[31].

IKZF2: Encodes for Zinc finger protein Helios, important for differentiation of fetal naïve to regulatory T-cells [61] and functional maturation of cochlear outer hair cells [62].

NPHS1: Encodes for Nephrin, member of immunoglobulin family of cell adhesion molecules, with function in glomerular filtration barrier in kidney [63], coding mutations cause Nephrotic syndrome, type 1 (MIM: 256300), which is characterized by proteinuria, hypoalbuminemia, hyperlipidemia, and edema.

PDE7A: Encodes for Phosphodiesterase 7A, a member of the cyclic nucleotide phosphodiesterase family, significantly higher expressed in cystic than in wild-type mouse kidneys [44].

PKHD1: Encodes for Fibrocystin, a membrane-associated receptor like protein, co-localizes with polycystin-2, a receptor-operated, nonselective cation channel; modulates renal tubular formation [40] and mitotic spindle assembly [41]; mutations cause Polycystic kidney disease 4 (MIM: 263200) .

PPDPF: Encodes for Pancreatic progenitor cell differentiation and proliferation factor, involved in exocrine pancreas development [49], prognostic marker for hepatocellular carcinoma [50].

RBM47: Encodes for RNA-binding protein 47, homolog in fish regulates innate antiviral immune response by negatively regulating interferone production [45].

RRAGD: Encodes for the Ras-related GTP-binding protein D, builds heterodimers with RRAGA or RRAGB, promotes mTORC1 activation[51,52].

SIGIRR: Encodes for transmembrane protein Single immunglobulin IL-1-related receptor, negatively regulates Interleukin 1 signaling [64].

SLC6A13: Encodes for the Sodium- and chloride-dependent GABA transporter 2, described as a high-affinity GABA and low-affinity taurine transporter, highly expressed in periportal hepatocytes and in proximal tubules in renal cortex in mouse, moderately expressed at blood-brain-barrier [53].

SPEG: Encodes for Striated muscle preferentially expressed protein kinase, important for maintenance of junctional membrane complexes integrity and sarcoplasmic reticulum Ca2+ handling in adult mammalian hearts [42], mutations cause Centronuclear myopathy 5 (MIM: 615959).

***TFDP2*:** Encodes for the transcription factor DP-2, builds heterodimer with E2F for DNA binding [54].

TPPP: Encodes for Tubulin polymerization-promoting protein, a regulator of microtubule dynamics [55].

YY1AP1: Encodes for Yin Yang 1 (YY1) associated protein 1, plays a role in transcriptional regulation, DNA repair and replication [56], rare mutations linked to Grange syndrome (MIM: 602531), which includes vascular disease similar to Fibromuscular dysplasia (arterial disease affecting renal arteries, MIM: 135580).

ZC3HC1: Encodes for Nuclear-interacting partner of ALK in SCF-type E3 ligase complex, which is regulated by cell-cycle-dependent phosphorylation and plays a role in mitotic entry by mediating ubiquitination and subsequent degradation of cyclin B1 [57].

Supplementary Note 4. DEPICT gene-set enrichment and gene prioritization.

We applied DEPICT to the genome-wide significant ($P<5x10^{-8}$) eGFRcrea variants mapping to 424 identified loci (using the primary meta-analysis GWAS), including tissue enrichment analysis, gene-set enrichment analysis and gene prioritisation based on co-functionality of gene pairs [5]. We found significant enrichment in 39 tissues (FDR≤5%, **Supplementary Data 18**), significant enrichment of 1,473 gene sets (FDR≤5%, **Supplementary Data 19**) and prioritization of 694 of our candidate genes (FDR≤0.05, **Supplementary Data 14**). We were interested whether enrichment in muscle tissue was reduced, when focussing on the 343 loci that were eGFRcys/BUN-validated. Now we found enrichment in 36 tissues, which still included kidney, but did not show enrichment in muscle (FDR≤5%, **Supplementary Data 18**). Furthermore, we found 1,173 enriched gene sets (FDR≤5%, **Supplementary Data 19**) and 559 prioritized candidate genes (FDR≤0.05, **Supplementary Data 14**). Among the 559 genes, 35 were not prioritized in the first DEPICT analysis. Both prioritization results were added to the GPS to enable individual restriction to eGFRcys/BUN validated-loci (**Supplementary Data 14**). Among the 22 highlighted genes (from **Table 2**), we found the following 17 prioritized also by DEPICT*: AMPD1, BCAS3, CDC14A, CERS2, CPXM1, GAB1, HNF1A, HOXD11, NPHS1,*

PDE7A, PKHD1, PPDPF, RBM47, SIGIRR, SPEG, TFDP2 and *YY1AP1* (**Supplementary Data 14**).

Supplementary Note 5. VA Million Veteran Program: Core Acknowledgement for Publications (Updated December 10, 2020).

MVP Executive Committee

- Co-Chair: J. Michael Gaziano, M.D., M.P.H.
  VA Boston Healthcare System, 150 S. Huntington Avenue, Boston, MA 02130
- Co-Chair: Sumitra Muralidhar, Ph.D.
  US Department of Veterans Affairs, 810 Vermont Avenue NW, Washington, DC 20420
- Rachel Ramoni, D.M.D., Sc.D., Chief VA Research and Development Officer
  US Department of Veterans Affairs, 810 Vermont Avenue NW, Washington, DC 20420
- Jean Beckham, Ph.D.
  Durham VA Medical Center, 508 Fulton Street, Durham, NC 27705
- Kyong-Mi Chang, M.D.
  Philadelphia VA Medical Center, 3900 Woodland Avenue, Philadelphia, PA 19104
- Christopher J. O'Donnell, M.D., M.P.H.
  VA Boston Healthcare System, 150 S. Huntington Avenue, Boston, MA 02130
- Philip S. Tsao, Ph.D.
  VA Palo Alto Health Care System, 3801 Miranda Avenue, Palo Alto, CA 94304
- James Breeling, M.D., Ex-Officio
  US Department of Veterans Affairs, 810 Vermont Avenue NW, Washington, DC 20420
- Grant Huang, Ph.D., Ex-Officio
  US Department of Veterans Affairs, 810 Vermont Avenue NW, Washington, DC 20420
- Juan P. Casas, M.D., Ph.D., Ex-Officio
  VA Boston Healthcare System, 150 S. Huntington Avenue, Boston, MA 02130

MVP Program Office

- Sumitra Muralidhar, Ph.D.
  US Department of Veterans Affairs, 810 Vermont Avenue NW, Washington, DC 20420
- Jennifer Moser, Ph.D.
  US Department of Veterans Affairs, 810 Vermont Avenue NW, Washington, DC 20420

MVP Recruitment/Enrollment

- Recruitment/Enrollment Director/Deputy Director, Boston – Stacey B. Whitbourne, Ph.D.; Jessica V. Brewer, M.P.H.
  VA Boston Healthcare System, 150 S. Huntington Avenue, Boston, MA 02130
- MVP Coordinating Centers
  - Clinical Epidemiology Research Center (CERC), West Haven – Mihaela Aslan, Ph.D.
    West Haven VA Medical Center, 950 Campbell Avenue, West Haven, CT 06516
  - Cooperative Studies Program Clinical Research Pharmacy Coordinating Center, Albuquerque – Todd Connor, Pharm.D.; Dean P. Argyres, B.S., M.S.
    New Mexico VA Health Care System, 1501 San Pedro Drive SE, Albuquerque, NM 87108
  - Genomics Coordinating Center, Palo Alto – Philip S. Tsao, Ph.D.
    VA Palo Alto Health Care System, 3801 Miranda Avenue, Palo Alto, CA 94304
  - MVP Boston Coordinating Center, Boston - J. Michael Gaziano, M.D., M.P.H.
    VA Boston Healthcare System, 150 S. Huntington Avenue, Boston, MA 02130
  - MVP Information Center, Canandaigua – Brady Stephens, M.S.

Canandaigua VA Medical Center, 400 Fort Hill Avenue, Canandaigua, NY 14424

- VA Central Biorepository, Boston – Mary T. Brophy M.D., M.P.H.; Donald E. Humphries, Ph.D.; Luis E. Selva, Ph.D.
  VA Boston Healthcare System, 150 S. Huntington Avenue, Boston, MA 02130
- MVP Informatics, Boston – Nhan Do, M.D.; Shahpoor (Alex) Shayan, M.S.
  VA Boston Healthcare System, 150 S. Huntington Avenue, Boston, MA 02130
- MVP Data Operations/Analytics, Boston – Kelly Cho, M.P.H., Ph.D.
  VA Boston Healthcare System, 150 S. Huntington Avenue, Boston, MA 02130
- Director of Regulatory Affairs – Lori Churby, B.S.
  VA Palo Alto Health Care System, 3801 Miranda Avenue, Palo Alto, CA 94304

MVP Science

- Science Operations – Christopher J. O'Donnell, M.D., M.P.H.
  VA Boston Healthcare System, 150 S. Huntington Avenue, Boston, MA 02130
- Genomics Core – Christopher J. O'Donnell, M.D., M.P.H.; Saiju Pyarajan Ph.D.
  VA Boston Healthcare System, 150 S. Huntington Avenue, Boston, MA 02130
  Philip S. Tsao, Ph.D.
  VA Palo Alto Health Care System, 3801 Miranda Avenue, Palo Alto, CA 94304
- Data Core – Kelly Cho, M.P.H, Ph.D.
  VA Boston Healthcare System, 150 S. Huntington Avenue, Boston, MA 02130
- VA Informatics and Computing Infrastructure (VINCI) – Scott L. DuVall, Ph.D.
  VA Salt Lake City Health Care System, 500 Foothill Drive, Salt Lake City, UT 84148
- Data and Computational Sciences – Saiju Pyarajan, Ph.D.
  VA Boston Healthcare System, 150 S. Huntington Avenue, Boston, MA 02130
- Statistical Genetics – Elizabeth Hauser, Ph.D.
  Durham VA Medical Center, 508 Fulton Street, Durham, NC 27705
  Yan Sun, Ph.D.
  Atlanta VA Medical Center, 1670 Clairmont Road, Decatur, GA 30033
  Hongyu Zhao, Ph.D.
  West Haven VA Medical Center, 950 Campbell Avenue, West Haven, CT 06516

Current MVP Local Site Investigators

- Atlanta VA Medical Center (Peter Wilson, M.D.)
  1670 Clairmont Road, Decatur, GA 30033
- Bay Pines VA Healthcare System (Rachel McArdle, Ph.D.)
  10,000 Bay Pines Blvd Bay Pines, FL 33744
- Birmingham VA Medical Center (Louis Dellitalia, M.D.)
  700 S. 19th Street, Birmingham AL 35233
- Central Western Massachusetts Healthcare System (Kristin Mattocks, Ph.D., M.P.H.)
  421 North Main Street, Leeds, MA 01053
- Cincinnati VA Medical Center (John Harley, M.D., Ph.D.)
  3200 Vine Street, Cincinnati, OH 45220
- Clement J. Zablocki VA Medical Center (Jeffrey Whittle, M.D., M.P.H.)
  5000 West National Avenue, Milwaukee, WI 53295
- VA Northeast Ohio Healthcare System (Frank Jacono, M.D.)
  10701 East Boulevard, Cleveland, OH 44106
- Durham VA Medical Center (Jean Beckham, Ph.D.)
  508 Fulton Street, Durham, NC 27705
- Edith Nourse Rogers Memorial Veterans Hospital (John Wells., Ph.D.)
  200 Springs Road, Bedford, MA 01730
- Edward Hines, Jr. VA Medical Center (Salvador Gutierrez, M.D.)
  5000 South 5th Avenue, Hines, IL 60141
- Veterans Health Care System of the Ozarks (Gretchen Gibson, D.D.S., M.P.H.)
  1100 North College Avenue, Fayetteville, AR 72703
- Fargo VA Health Care System (Kimberly Hammer, Ph.D.)
  2101 N. Elm, Fargo, ND 58102
- VA Health Care Upstate New York (Laurence Kaminsky, Ph.D.)

113 Holland Avenue, Albany, NY 12208

- New Mexico VA Health Care System (Gerardo Villareal, M.D.)
  1501 San Pedro Drive, S.E. Albuquerque, NM 87108
- VA Boston Healthcare System (Scott Kinlay, M.B.B.S., Ph.D.)
  150 S. Huntington Avenue, Boston, MA 02130
- VA Western New York Healthcare System (Junzhe Xu, M.D.)
  3495 Bailey Avenue, Buffalo, NY 14215-1199
- Ralph H. Johnson VA Medical Center (Mark Hamner, M.D.)
  109 Bee Street, Mental Health Research, Charleston, SC 29401
- Columbia VA Health Care System (Roy Mathew, M.D.)
  6439 Garners Ferry Road, Columbia, SC 29209
- VA North Texas Health Care System (Sujata Bhushan, M.D.)
  4500 S. Lancaster Road, Dallas, TX 75216
- Hampton VA Medical Center (Pran Iruvanti, D.O., Ph.D.)
  100 Emancipation Drive, Hampton, VA 23667
- Richmond VA Medical Center (Michael Godschalk, M.D.)
  1201 Broad Rock Blvd., Richmond, VA 23249
- Iowa City VA Health Care System (Zuhair Ballas, M.D.)
  601 Highway 6 West, Iowa City, IA 52246-2208
- Eastern Oklahoma VA Health Care System (Douglas Ivins, M.D.)
  1011 Honor Heights Drive, Muskogee, OK 74401
- James A. Haley Veterans' Hospital (Stephen Mastorides, M.D.)
  13000 Bruce B. Downs Blvd, Tampa, FL 33612
- James H. Quillen VA Medical Center (Jonathan Moorman, M.D., Ph.D.)
  Corner of Lamont & Veterans Way, Mountain Home, TN 37684
- John D. Dingell VA Medical Center (Saib Gappy, M.D.)
  4646 John R Street, Detroit, MI 48201
- Louisville VA Medical Center (Jon Klein, M.D., Ph.D.)
  800 Zorn Avenue, Louisville, KY 40206
- Manchester VA Medical Center (Nora Ratcliffe, M.D.)
  718 Smyth Road, Manchester, NH 03104
- Miami VA Health Care System (Hermes Florez, M.D., Ph.D.)
  1201 NW 16th Street, 11 GRC, Miami FL 33125
- Michael E. DeBakey VA Medical Center (Olaoluwa Okusaga, M.D.)
  2002 Holcombe Blvd, Houston, TX 77030
- Minneapolis VA Health Care System (Maureen Murdoch, M.D., M.P.H.)
  One Veterans Drive, Minneapolis, MN 55417
- N. FL/S. GA Veterans Health System (Peruvemba Sriram, M.D.)
  1601 SW Archer Road, Gainesville, FL 32608
- Northport VA Medical Center (Shing Shing Yeh, Ph.D., M.D.)
  79 Middleville Road, Northport, NY 11768
- Overton Brooks VA Medical Center (Neeraj Tandon, M.D.)
  510 East Stoner Ave, Shreveport, LA 71101
- Philadelphia VA Medical Center (Darshana Jhala, M.D.)
  3900 Woodland Avenue, Philadelphia, PA 19104
- Phoenix VA Health Care System (Samuel Aguayo, M.D.)
  650 E. Indian School Road, Phoenix, AZ 85012
- Portland VA Medical Center (David Cohen, M.D.)
  3710 SW U.S. Veterans Hospital Road, Portland, OR 97239
- Providence VA Medical Center (Satish Sharma, M.D.)
  830 Chalkstone Avenue, Providence, RI 02908
- Richard Roudebush VA Medical Center (Suthat Liangpunsakul, M.D., M.P.H.)
  1481 West 10th Street, Indianapolis, IN 46202
- Salem VA Medical Center (Kris Ann Oursler, M.D.)
  1970 Roanoke Blvd, Salem, VA 24153

- San Francisco VA Health Care System (Mary Whooley, M.D.)
  4150 Clement Street, San Francisco, CA 94121
- South Texas Veterans Health Care System (Sunil Ahuja, M.D.)
  7400 Merton Minter Boulevard, San Antonio, TX 78229
- Southeast Louisiana Veterans Health Care System (Joseph Constans, Ph.D.)
  2400 Canal Street, New Orleans, LA 70119
- Southern Arizona VA Health Care System (Paul Meyer, M.D., Ph.D.)
  3601 S 6th Avenue, Tucson, AZ 85723
- Sioux Falls VA Health Care System (Jennifer Greco, M.D.)
  2501 W 22nd Street, Sioux Falls, SD 57105
- St. Louis VA Health Care System (Michael Rauchman, M.D.)
  915 North Grand Blvd, St. Louis, MO 63106
- Syracuse VA Medical Center (Richard Servatius, Ph.D.)
  800 Irving Avenue, Syracuse, NY 13210
- VA Eastern Kansas Health Care System (Melinda Gaddy, Ph.D.)
  4101 S 4th Street Trafficway, Leavenworth, KS 66048
- VA Greater Los Angeles Health Care System (Agnes Wallbom, M.D., M.S.)
  11301 Wilshire Blvd, Los Angeles, CA 90073
- VA Long Beach Healthcare System (Timothy Morgan, M.D.)
  5901 East 7th Street Long Beach, CA 90822
- VA Maine Healthcare System (Todd Stapley, D.O.)
  1 VA Center, Augusta, ME 04330
- VA New York Harbor Healthcare System (Scott Sherman, M.D., M.P.H.)
  423 East 23rd Street, New York, NY 10010
- VA Pacific Islands Health Care System (George Ross, M.D.)
  459 Patterson Rd, Honolulu, HI 96819
- VA Palo Alto Health Care System (Philip Tsao, Ph.D.)
  3801 Miranda Avenue, Palo Alto, CA 94304-1290
- VA Pittsburgh Health Care System (Patrick Strollo, Jr., M.D.)
  University Drive, Pittsburgh, PA 15240
- VA Puget Sound Health Care System (Edward Boyko, M.D.)
  1660 S. Columbian Way, Seattle, WA 98108-1597
- VA Salt Lake City Health Care System (Laurence Meyer, M.D., Ph.D.)
  500 Foothill Drive, Salt Lake City, UT 84148
- VA San Diego Healthcare System (Samir Gupta, M.D., M.S.C.S.)
  3350 La Jolla Village Drive, San Diego, CA 92161
- VA Sierra Nevada Health Care System (Mostaqul Huq, Pharm.D., Ph.D.)
  975 Kirman Avenue, Reno, NV 89502
- VA Southern Nevada Healthcare System (Joseph Fayad, M.D.)
  6900 North Pecos Road, North Las Vegas, NV 89086
- VA Tennessee Valley Healthcare System (Adriana Hung, M.D., M.P.H.)
  1310 24th Avenue, South Nashville, TN 37212
- Washington DC VA Medical Center (Jack Lichy, M.D., Ph.D.)
  50 Irving St, Washington, D. C. 20422
- W.G. (Bill) Hefner VA Medical Center (Robin Hurley, M.D.)
  1601 Brenner Ave, Salisbury, NC 28144
- White River Junction VA Medical Center (Brooks Robey, M.D.)
  163 Veterans Drive, White River Junction, VT 05009
- William S. Middleton Memorial Veterans Hospital (Robert Striker, M.D., Ph.D.)
  2500 Overlook Terrace, Madison, WI 53705

SUPPLEMENTARY TABLES

Supplementary Table 1. Descriptives of genetic risk score (GRS) studies AugUR and HUNT.

Study	Variable	n	mean	sd	min	max	5th percentile	median	95th percentile
AugUR	unweighted GRS [# risk alleles]	1,105	626.5	14.6	584.1	674.9	602.4	626.5	650.7
	weighted GRS		1.686	0.037	1.564	1.803	1.628	1.685	1.749
	eGFR [ml/min/1.73m2]		67.0	15.5	18.0	104.4	37.0	68.1	88.0
	eGFR residuals (age-/sex-adjusted)		0.00	14.59	-50.76	31.36	-26.40	0.86	19.53
	eGFR residuals (age-/sex-/PC-adjusted)		0.00	14.60	-50.80	32.09	-26.40	0.91	19.53
HUNT	unweighted GRS [# risk alleles]	26,254	614.6	15.4	554.0	683.0	589.0	615.0	640.0
	weighted GRS		1.652	0.039	1.478	1.806	1.587	1.652	1.717
	eGFR [ml/min/1.73m2]		100.1	18.7	15.0	172.2	66.3	101.9	127.2
	eGFR residuals (age-/sex-adjusted)		0.08	11.95	-82.66	50.74	-22.83	2.80	15.15
	eGFR residuals (age-/sex-/PC-adjusted)		0.00	11.93	-83.43	50.48	-22.89	2.66	15.07

REFERENCES

1. Wuttke, M. *et al.* A catalog of genetic loci associated with kidney function from analyses of a million individuals. *Nat. Genet.* **51**, 957–972 (2019).

2. Pruim, R. J. *et al.* LocusZoom: Regional visualization of genome-wide association scan results. in *Bioinformatics* (2011). doi:10.1093/bioinformatics/btq419

3. Yang, J. *et al.* Conditional and joint multiple-SNP analysis of GWAS summary statistics identifies additional variants influencing complex traits. *Nat. Genet.* **44**, 369–375 (2012).

4. Gillies, C. E. *et al.* An eQTL Landscape of Kidney Tissue in Human Nephrotic Syndrome. *Am. J. Hum. Genet.* (2018). doi:10.1016/j.ajhg.2018.07.004

5. Pers, T. H. *et al.* Biological interpretation of genome-wide association studies using predicted gene functions. *Nat. Commun.* **6**, 5890 (2015).
