# Supplementary material for: Discovery and prioritization of variants and genes for kidney function in >1.2 million individuals
Source: Nat Commun. 2021 Jul 16;12:4350. doi: 10.1038/s41467-021-24491-0 (PMC8285412; doi:10.1038/s41467-021-24491-0)
Supplement: Supplementary file 2 — Description of Additional Supplementary Files [file 41467_2021_24491_MOESM2_ESM.pdf]

Description of Additional Supplementary Files

File Name: Supplementary Data 1
Description: Details on the UK biobank and the second meta-analyis studies

File Name: Supplementary Data 2
Description: Association statistics for lead variants of 424 genome-wide significant (P<5x10e-8) eGFRcrea loci

File Name: Supplementary Data 3
Description: Results for 264 lead variants from Wuttke et al

File Name: Supplementary Data 4
Description: Secondary meta-analysis and combined primary + secondary meta-analysis association results for lead variants of 424 genome-wide significant (P<5x10e-8) eGFRcrea loci

File Name: Supplementary Data 5
Description: Meta-analysis results for eGFRcys and BUN

File Name: Supplementary Data 6
Description: Multiple independent signals and explained variance

File Name: Supplementary Data 7
Description: Statistical fine mapping results

File Name: Supplementary Data 8
Description: Signal table of 634 independent signals at 424 eGFRcrea associated loci

File Name: Supplementary Data 9
Description: CADD results and annotations

File Name: Supplementary Data 10
Description: Significant eQTLs in NEPTUNE glomerular or tubulo-interstitial tissue

File Name: Supplementary Data 11
Description: Significant eQTLs or sQTLs in GTEx kidney tissue

File Name: Supplementary Data 12
Description:

a. Significant eQTLs in GTEx tissues (except kidney) at known loci.
b. Significant eQTLs in GTEx tissues (except kidney) at novel loci.
c. Significant sQTLs in GTEx tissues (except kidney) at known loci.
d. Significant sQTLs in GTEx tissues (except kidney) at novel loci.

File Name: Supplementary Data 13
Description: Annotation of the 138 small credible sets

File Name: Supplementary Data 14

Description:

a. Genepriotisation (GPS) for the 424 eGFRcrea loci in a per signal view
b. Genepriotisation (GPS) for the 424 eGFRcrea loci in a per locus view

File Name: Supplementary Data 15
Description: Mouse Genome Informatics (MGI) kidney entries

File Name: Supplementary Data 16
Description: Monogenic kidney-related diseases or phenotypes in human

File Name: Supplementary Data 17
Description: Tissue and cell-type specific enrichment analyses in data by GTEx, Wu et al and Stewart et. al

File Name: Supplementary Data 18
Description: Tissue enrichment analyses by DEPICT

File Name: Supplementary Data 19
Description: DEPICT gene set enrichment analyses

File Name: Supplementary Data 20
Description: Celltype-specific gene-expression (Wu et al and Stewart et al data)

File Name: Supplementary Data 21
Description: Colocalization results in NEPTUNE tissues

File Name: Supplementary Data 22
Description: Celltype specific heriatbility based on LDSC and LDSC-seg
