# Supplementary material for: Discovery and prioritization of variants and genes for kidney function in >1.2 million individuals
Source: Nat Commun. 2021 Jul 16;12:4350. doi: 10.1038/s41467-021-24491-0 (PMC8285412; doi:10.1038/s41467-021-24491-0)
Supplement: Supplementary file 29 — Reporting Summary [file 41467_2021_24491_MOESM29_ESM.pdf]

Corresponding author(s): Thomas Winkler

Last updated by author(s): Jun 3, 2021

Reporting Summary

Nature Research wishes to improve the reproducibility of the work that we publish. This form provides structure for consistency and transparency in reporting. For further information on Nature Research policies, see our Editorial Policies and the Editorial Policy Checklist.

Statistics

For all statistical analyses, confirm that the following items are present in the figure legend, table legend, main text, or Methods section.

n/a	Confirmed	
☐	☒	The exact sample size (*n*) for each experimental group/condition, given as a discrete number and unit of measurement
☐	☒	A statement on whether measurements were taken from distinct samples or whether the same sample was measured repeatedly
☐	☒	The statistical test(s) used AND whether they are one- or two-sided<br>*Only common tests should be described solely by name; describe more complex techniques in the Methods section.*
☐	☒	A description of all covariates tested
☐	☒	A description of any assumptions or corrections, such as tests of normality and adjustment for multiple comparisons
☐	☒	A full description of the statistical parameters including central tendency (e.g. means) or other basic estimates (e.g. regression coefficient) AND variation (e.g. standard deviation) or associated estimates of uncertainty (e.g. confidence intervals)
☐	☒	For null hypothesis testing, the test statistic (e.g. *F*, *t*, *r*) with confidence intervals, effect sizes, degrees of freedom and *P* value noted<br>*Give P values as exact values whenever suitable.*
☐	☒	For Bayesian analysis, information on the choice of priors and Markov chain Monte Carlo settings
☐	☒	For hierarchical and complex designs, identification of the appropriate level for tests and full reporting of outcomes
☐	☒	Estimates of effect sizes (e.g. Cohen's *d*, Pearson's *r*), indicating how they were calculated

Our web collection on statistics for biologists contains articles on many of the points above.

Software and code

Policy information about availability of computer code

Data collection	EasyQC (v 20.8) was used to clean the GWAS data collected.
Data analysis	fastGWAS was used for the GWAS of UK Biobank.

For manuscripts utilizing custom algorithms or software that are central to the research but not yet described in published literature, software must be made available to editors and reviewers. We strongly encourage code deposition in a community repository (e.g. GitHub). See the Nature Research guidelines for submitting code & software for further information.

Data

Policy information about availability of data

All manuscripts must include a data availability statement. This statement should provide the following information, where applicable:

- Accession codes, unique identifiers, or web links for publicly available datasets
- A list of figures that have associated raw data
- A description of any restrictions on data availability

Summary genetic association results for UKB and the meta-analysis of UKB and CKDGen for log(eGFRcrea), log(eGFRcys) and log(BUN) can be downloaded from www.genepi-regensburg.de/ckd or from https://ckdgen.imbi.uni-freiburg.de/. Previously published association results for eGFRcrea can be found at https://ckdgen.imbi.uni-freiburg.de/. The GPS table is also available from www.genepi-regensburg.de/ckd.

Field-specific reporting

Please select the one below that is the best fit for your research. If you are not sure, read the appropriate sections before making your selection.

[x] Life sciences [ ] Behavioural & social sciences [ ] Ecological, evolutionary & environmental sciences

For a reference copy of the document with all sections, see nature.com/documents/nr-reporting-summary-flat.pdf

Life sciences study design

All studies must disclose on these points even when the disclosure is negative.

Sample size	n = 765,348 individuals were used for the CKDGen GWAS meta-analysis; 436,581 individuals from UK Biobank
Data exclusions	We excluded variants with a low imputation quality (Info<0.6), very rare variants with MAF<0.1%, variants that were exclusively available in CKDGen or UKB. This led to the exclusion of insertions, deletions and structural variants from the UKB GWAS because CKDGen focused on SNPs previously, Wuttke et al 2019.
Replication	Second stage meta-analysis of three studies (n=400,000).
Randomization	Main analyses included UKB GWAS; UKB is a prospective cohort study of 500K; we included related individuals and accounted for relationship by using a mixed model approach.
Blinding	No blinding needed for the GWAS .

Reporting for specific materials, systems and methods

We require information from authors about some types of materials, experimental systems and methods used in many studies. Here, indicate whether each material, system or method listed is relevant to your study. If you are not sure if a list item applies to your research, read the appropriate section before selecting a response.

Materials & experimental systems

n/a	Involved in the study	
[x]	[ ]	Antibodies
[x]	[ ]	Eukaryotic cell lines
[x]	[ ]	Palaeontology and archaeology
[x]	[ ]	Animals and other organisms
[x]	[ ]	Human research participants
[x]	[ ]	Clinical data
[x]	[ ]	Dual use research of concern

Methods

n/a	Involved in the study	
[x]	[ ]	ChIP-seq
[x]	[ ]	Flow cytometry
[x]	[ ]	MRI-based neuroimaging
